# Supplementary figures and images for: JAK-STAT1 as therapeutic target for EGFR deficiency-associated inflammation and scarring alopecia
Source: EMBO Mol Med. 2024 Nov 9;16(12):7. doi: 10.1038/s44321-024-00166-3 (PMC11628629; doi:10.1038/s44321-024-00166-3)

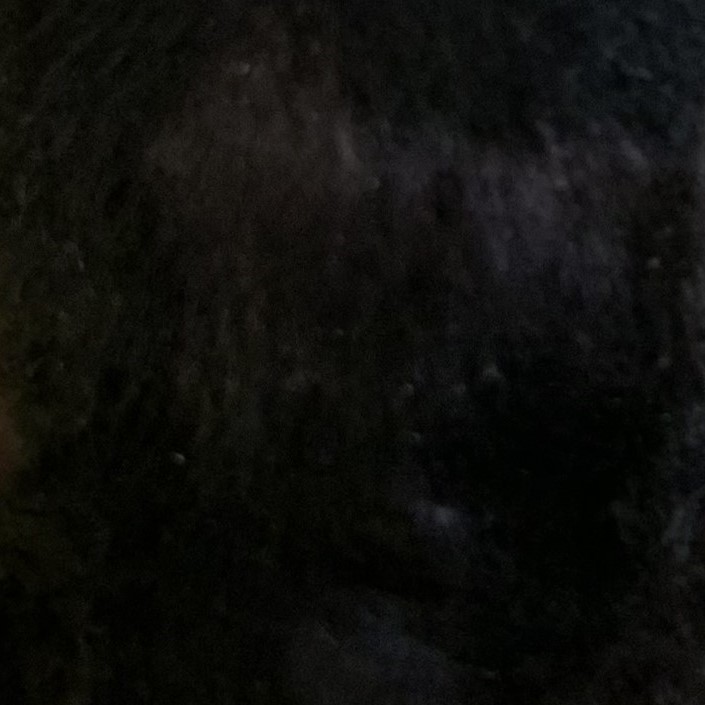

Supplement: Supplementary file 2 — Source data Fig. 1 [file 44321_2024_166_MOESM2_ESM.zip › EMM-2024-20141-V2_Source data for Figure 1/Figure 1A Image data/2M Dep ABX.jpeg]

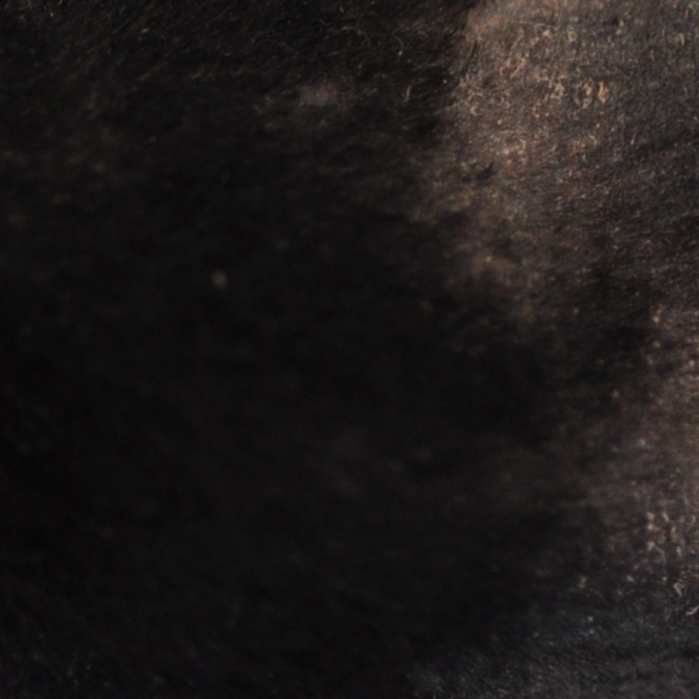

Supplement: Supplementary file 2 — Source data Fig. 1 [file 44321_2024_166_MOESM2_ESM.zip › EMM-2024-20141-V2_Source data for Figure 1/Figure 1A Image data/2M Dep SOS.JPG]

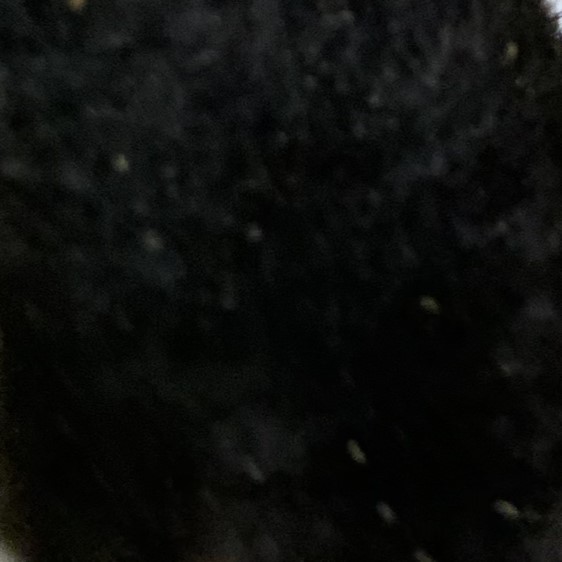

Supplement: Supplementary file 2 — Source data Fig. 1 [file 44321_2024_166_MOESM2_ESM.zip › EMM-2024-20141-V2_Source data for Figure 1/Figure 1A Image data/2M Dep.jpg]

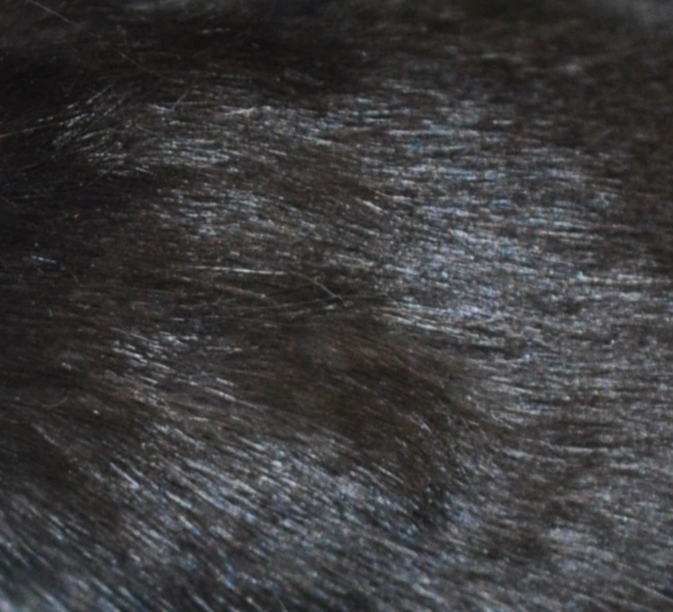

Supplement: Supplementary file 2 — Source data Fig. 1 [file 44321_2024_166_MOESM2_ESM.zip › EMM-2024-20141-V2_Source data for Figure 1/Figure 1A Image data/2M WT.JPG]

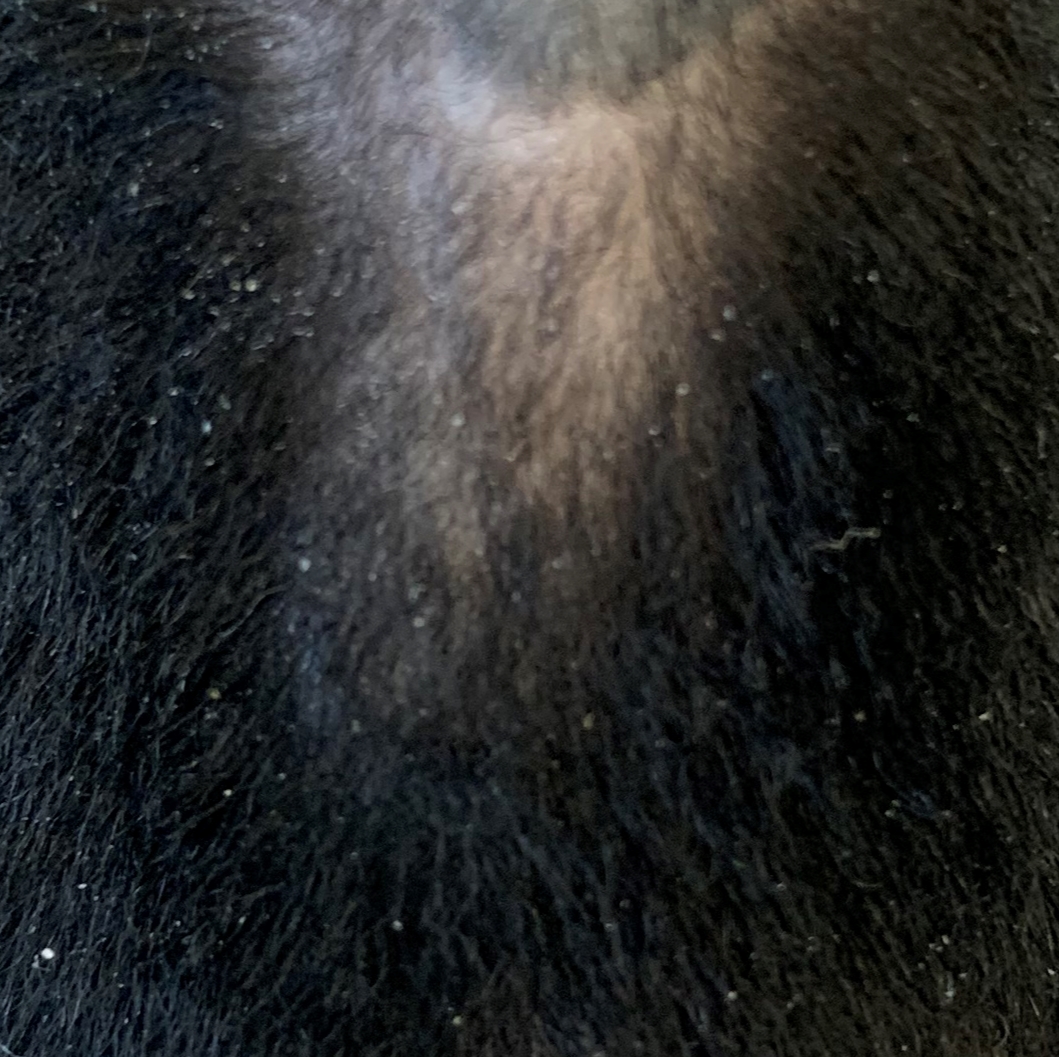

Supplement: Supplementary file 2 — Source data Fig. 1 [file 44321_2024_166_MOESM2_ESM.zip › EMM-2024-20141-V2_Source data for Figure 1/Figure 1A Image data/5M Dep ABX.jpg]

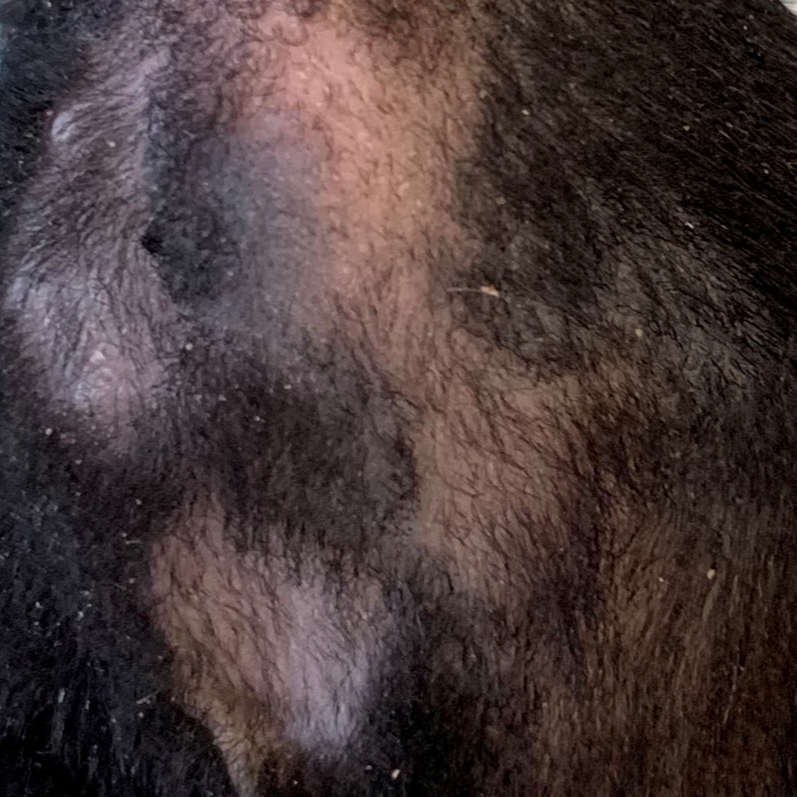

Supplement: Supplementary file 2 — Source data Fig. 1 [file 44321_2024_166_MOESM2_ESM.zip › EMM-2024-20141-V2_Source data for Figure 1/Figure 1A Image data/5M Dep SOS.jpg]

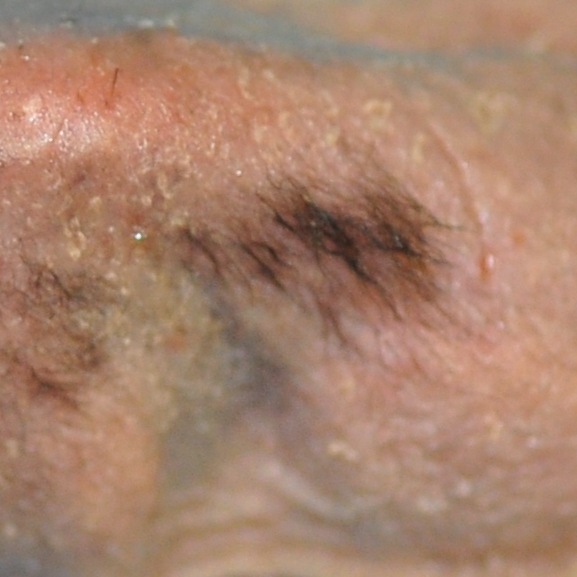

Supplement: Supplementary file 2 — Source data Fig. 1 [file 44321_2024_166_MOESM2_ESM.zip › EMM-2024-20141-V2_Source data for Figure 1/Figure 1A Image data/5M Dep.JPG]

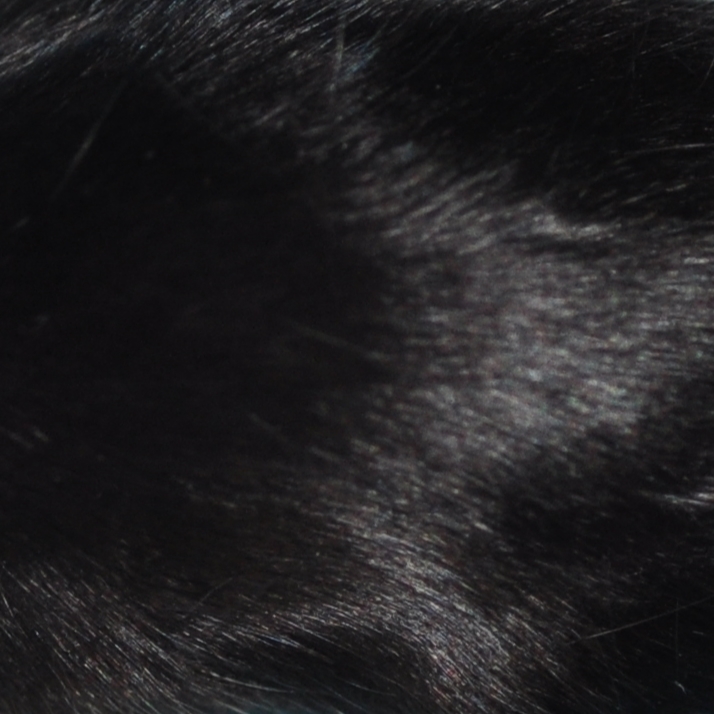

Supplement: Supplementary file 2 — Source data Fig. 1 [file 44321_2024_166_MOESM2_ESM.zip › EMM-2024-20141-V2_Source data for Figure 1/Figure 1A Image data/5M WT.jpg]

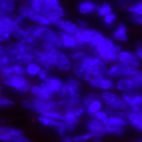

Supplement: Supplementary file 2 — Source data Fig. 1 [file 44321_2024_166_MOESM2_ESM.zip › EMM-2024-20141-V2_Source data for Figure 1/Figure 1C Image data/Cut EGR2 blue.tif]

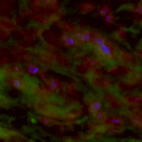

Supplement: Supplementary file 2 — Source data Fig. 1 [file 44321_2024_166_MOESM2_ESM.zip › EMM-2024-20141-V2_Source data for Figure 1/Figure 1C Image data/Cut EGR2 green.tif]

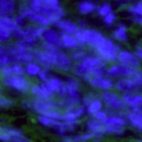

Supplement: Supplementary file 2 — Source data Fig. 1 [file 44321_2024_166_MOESM2_ESM.zip › EMM-2024-20141-V2_Source data for Figure 1/Figure 1C Image data/Cut EGR2.tif]

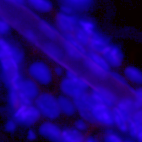

Supplement: Supplementary file 2 — Source data Fig. 1 [file 44321_2024_166_MOESM2_ESM.zip › EMM-2024-20141-V2_Source data for Figure 1/Figure 1C Image data/Cut WT blue.tif]

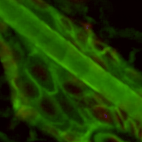

Supplement: Supplementary file 2 — Source data Fig. 1 [file 44321_2024_166_MOESM2_ESM.zip › EMM-2024-20141-V2_Source data for Figure 1/Figure 1C Image data/Cut WT green.tif]

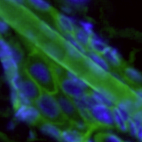

Supplement: Supplementary file 2 — Source data Fig. 1 [file 44321_2024_166_MOESM2_ESM.zip › EMM-2024-20141-V2_Source data for Figure 1/Figure 1C Image data/Cut WT.tif]

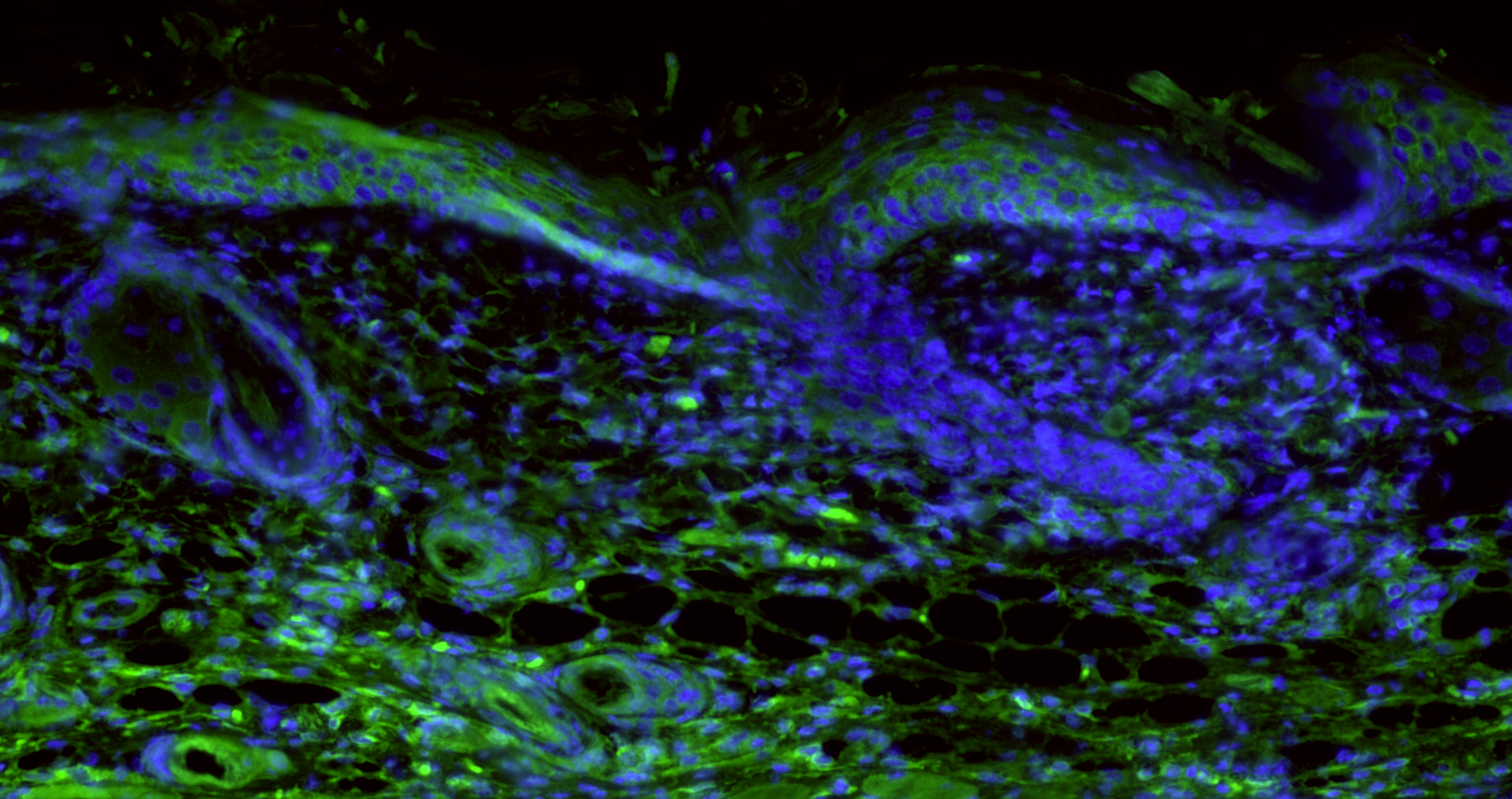

Supplement: Supplementary file 2 — Source data Fig. 1 [file 44321_2024_166_MOESM2_ESM.zip › EMM-2024-20141-V2_Source data for Figure 1/Figure 1C Image data/EGR2 20x1.tif]

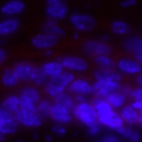

Supplement: Supplementary file 2 — Source data Fig. 1 [file 44321_2024_166_MOESM2_ESM.zip › EMM-2024-20141-V2_Source data for Figure 1/Figure 1C Image data/IFEblue.tif]

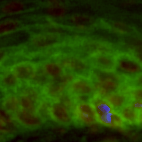

Supplement: Supplementary file 2 — Source data Fig. 1 [file 44321_2024_166_MOESM2_ESM.zip › EMM-2024-20141-V2_Source data for Figure 1/Figure 1C Image data/IFEgreen.tif]

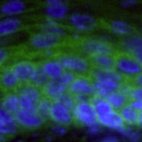

Supplement: Supplementary file 2 — Source data Fig. 1 [file 44321_2024_166_MOESM2_ESM.zip › EMM-2024-20141-V2_Source data for Figure 1/Figure 1C Image data/IFEMerged.tif]

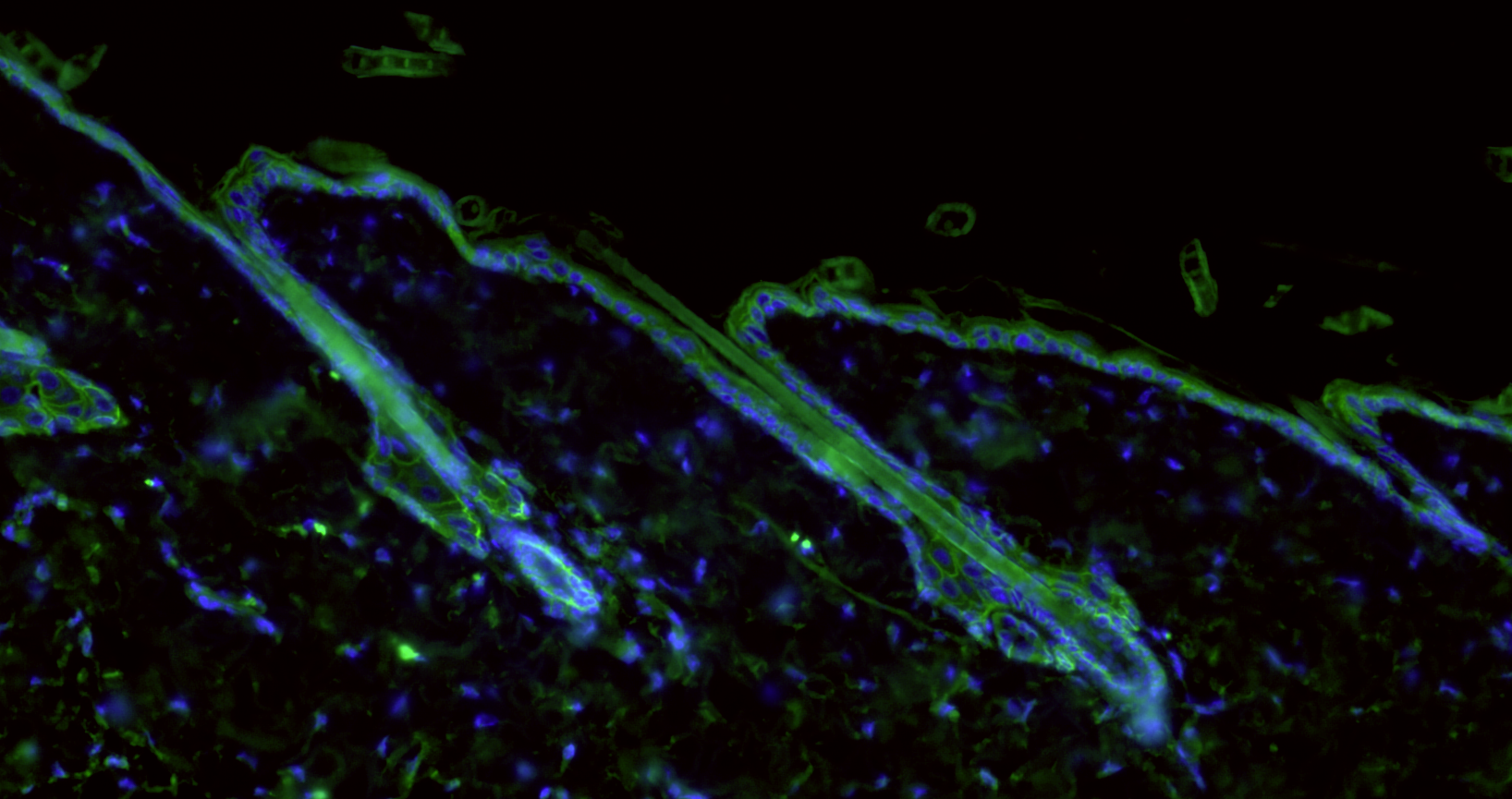

Supplement: Supplementary file 2 — Source data Fig. 1 [file 44321_2024_166_MOESM2_ESM.zip › EMM-2024-20141-V2_Source data for Figure 1/Figure 1C Image data/WT 20x1.tif]

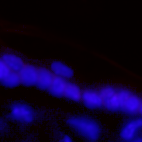

Supplement: Supplementary file 2 — Source data Fig. 1 [file 44321_2024_166_MOESM2_ESM.zip › EMM-2024-20141-V2_Source data for Figure 1/Figure 1C Image data/WTIFEblue.tif]

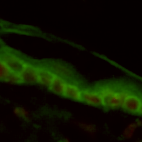

Supplement: Supplementary file 2 — Source data Fig. 1 [file 44321_2024_166_MOESM2_ESM.zip › EMM-2024-20141-V2_Source data for Figure 1/Figure 1C Image data/WTIFEgreen.tif]

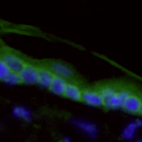

Supplement: Supplementary file 2 — Source data Fig. 1 [file 44321_2024_166_MOESM2_ESM.zip › EMM-2024-20141-V2_Source data for Figure 1/Figure 1C Image data/WTIFEmerged.tif]

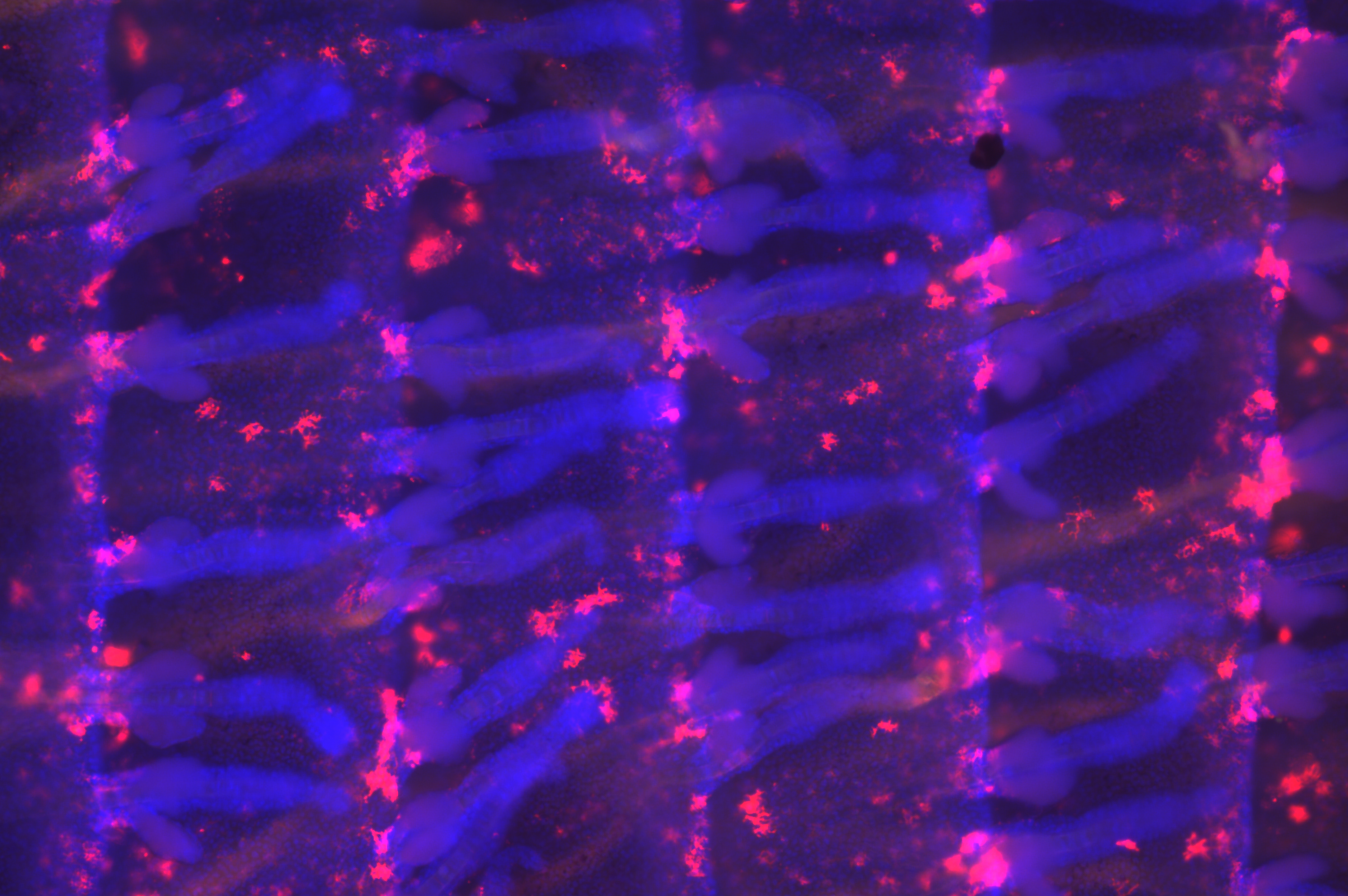

Supplement: Supplementary file 2 — Source data Fig. 1 [file 44321_2024_166_MOESM2_ESM.zip › EMM-2024-20141-V2_Source data for Figure 1/Figure 1D Image data/Fig 1D KO 20x 5.tif]

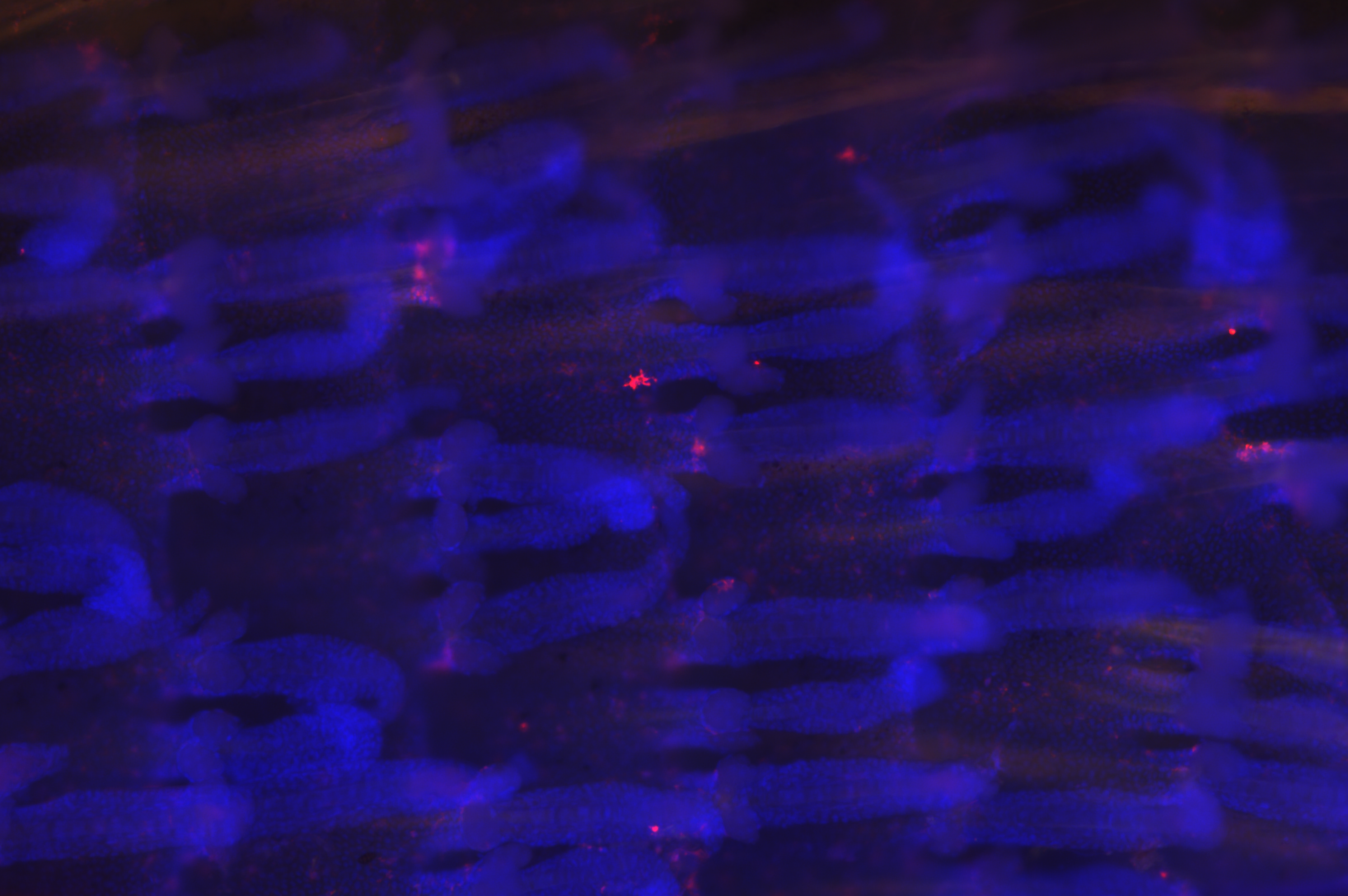

Supplement: Supplementary file 2 — Source data Fig. 1 [file 44321_2024_166_MOESM2_ESM.zip › EMM-2024-20141-V2_Source data for Figure 1/Figure 1D Image data/Fig 1D WT 20x 3.tif]

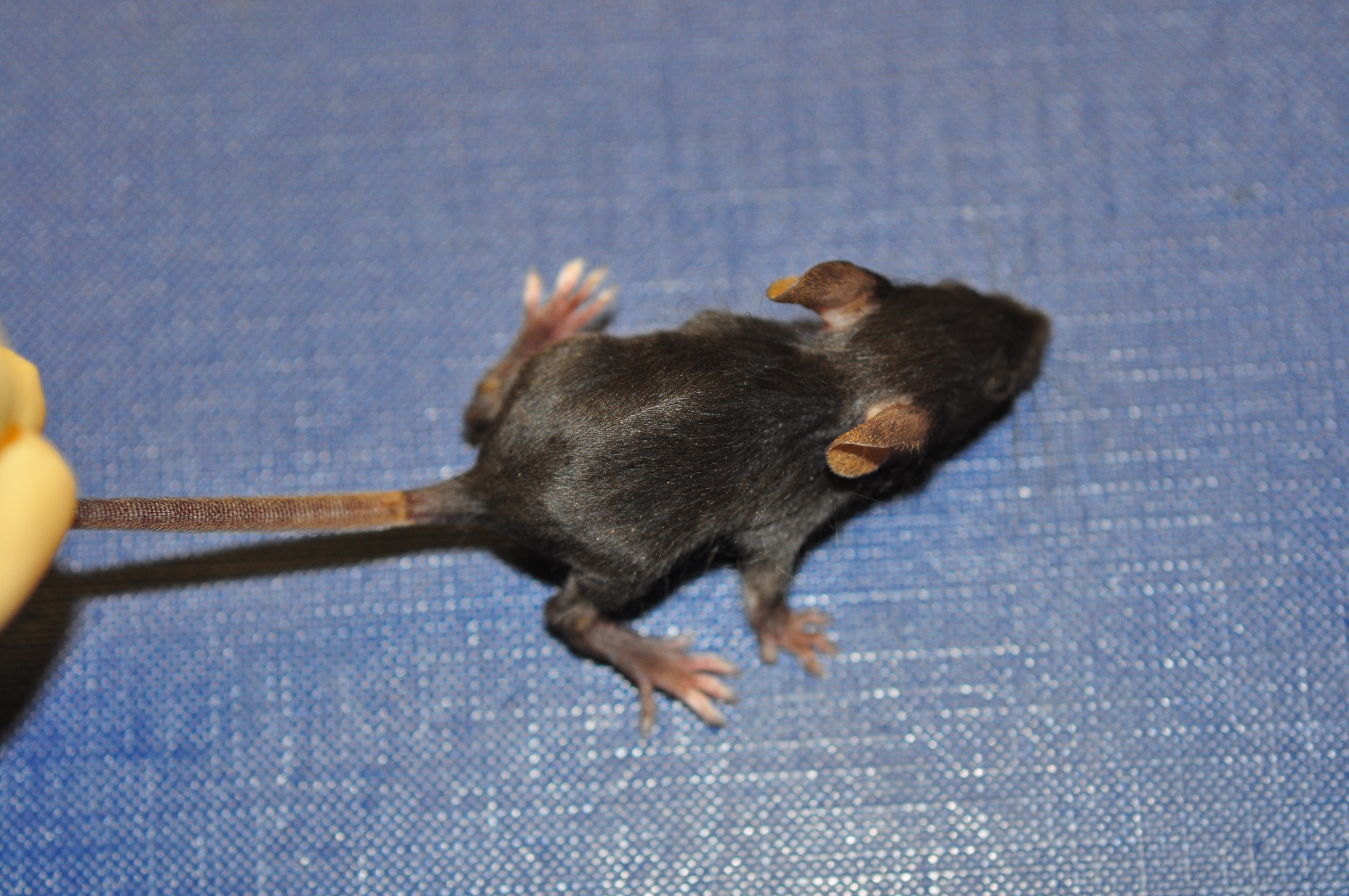

Supplement: Supplementary file 2 — Source data Fig. 1 [file 44321_2024_166_MOESM2_ESM.zip › EMM-2024-20141-V2_Source data for Figure 1/Figure 1E Image data/Fig 1E DEgr2 1M.JPG]

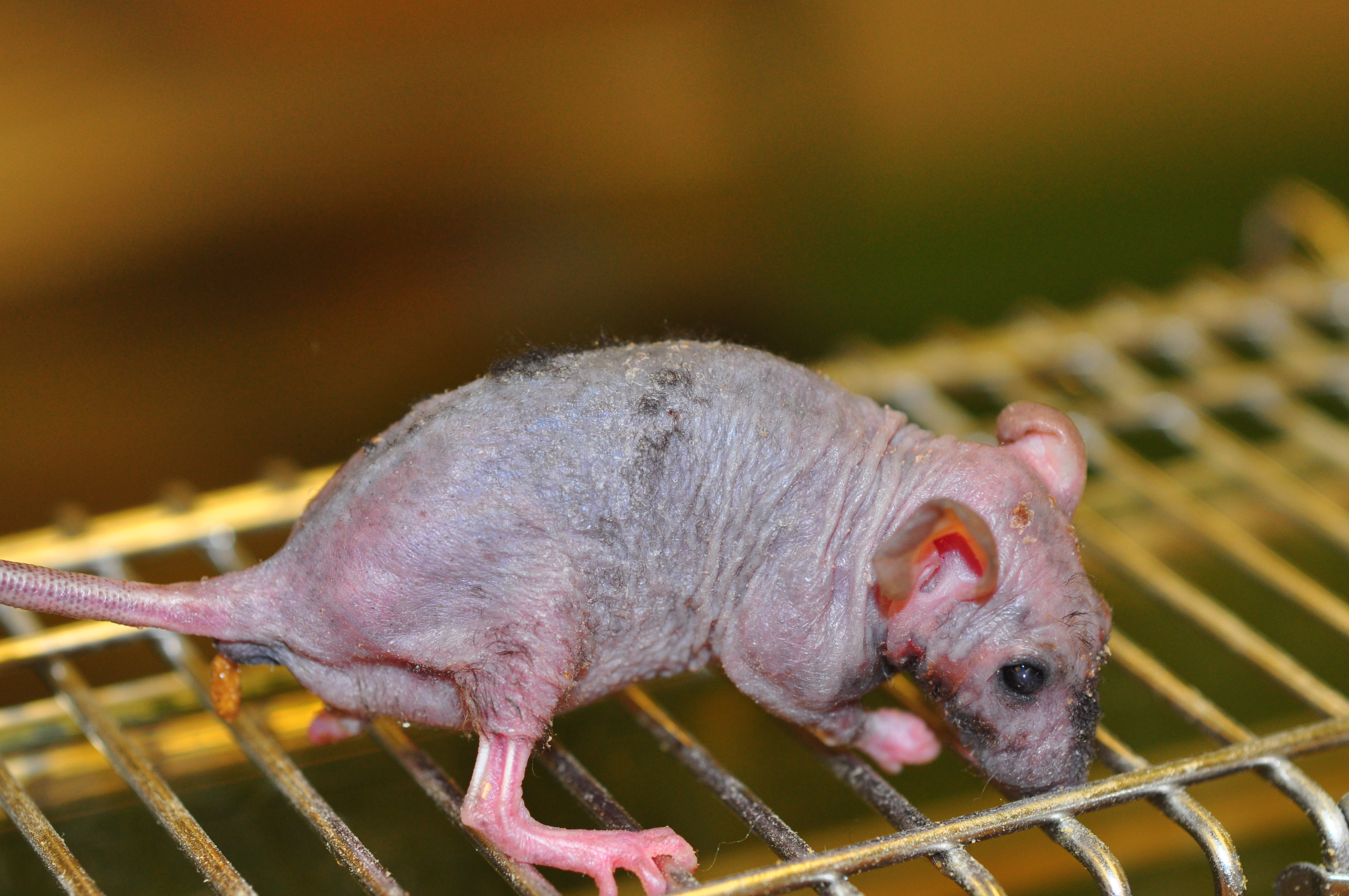

Supplement: Supplementary file 2 — Source data Fig. 1 [file 44321_2024_166_MOESM2_ESM.zip › EMM-2024-20141-V2_Source data for Figure 1/Figure 1E Image data/Fig 1E DEgr2 5M.JPG]

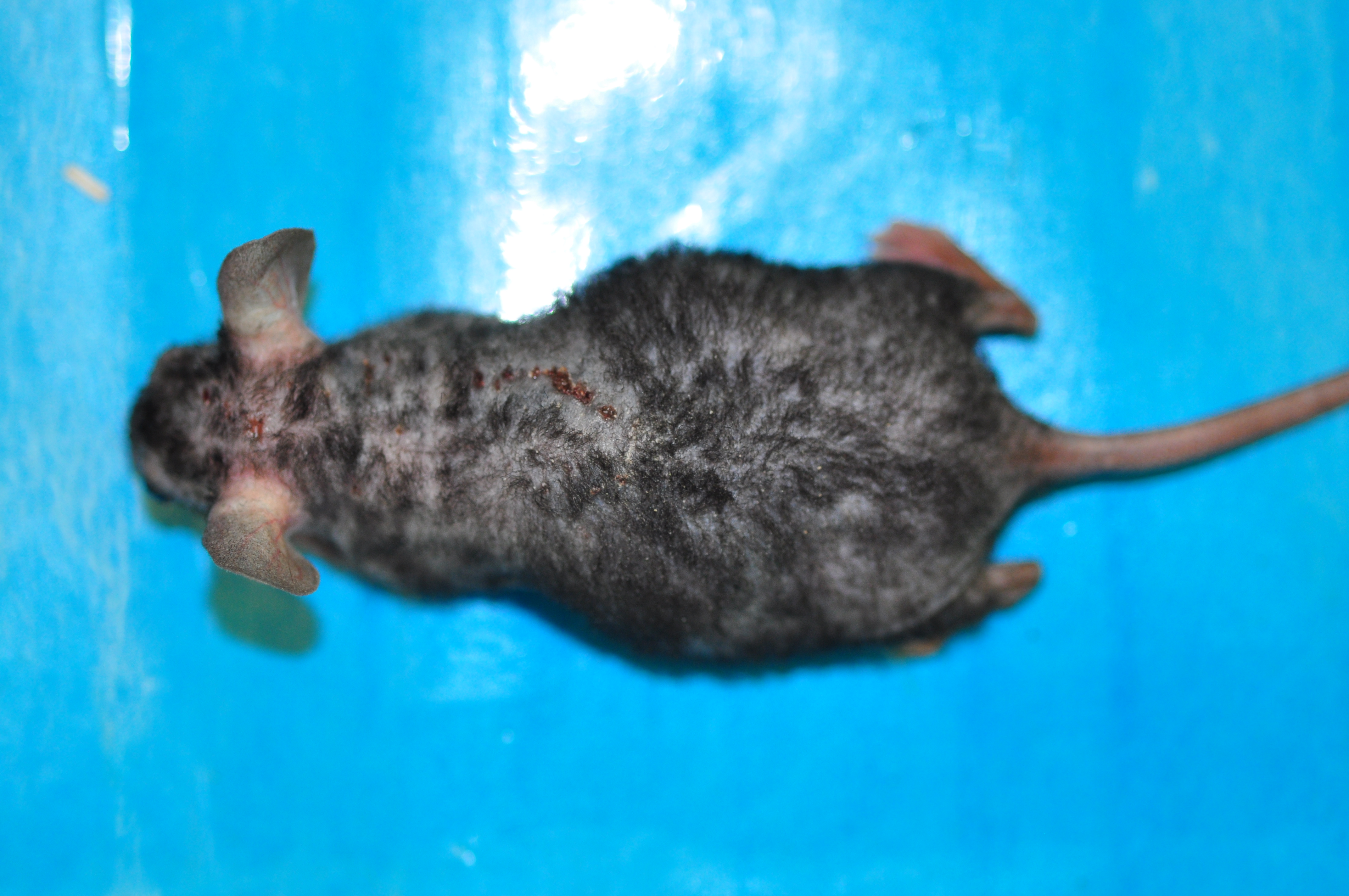

Supplement: Supplementary file 2 — Source data Fig. 1 [file 44321_2024_166_MOESM2_ESM.zip › EMM-2024-20141-V2_Source data for Figure 1/Figure 1E Image data/Fig 1E DEgr2 Abx.JPG]

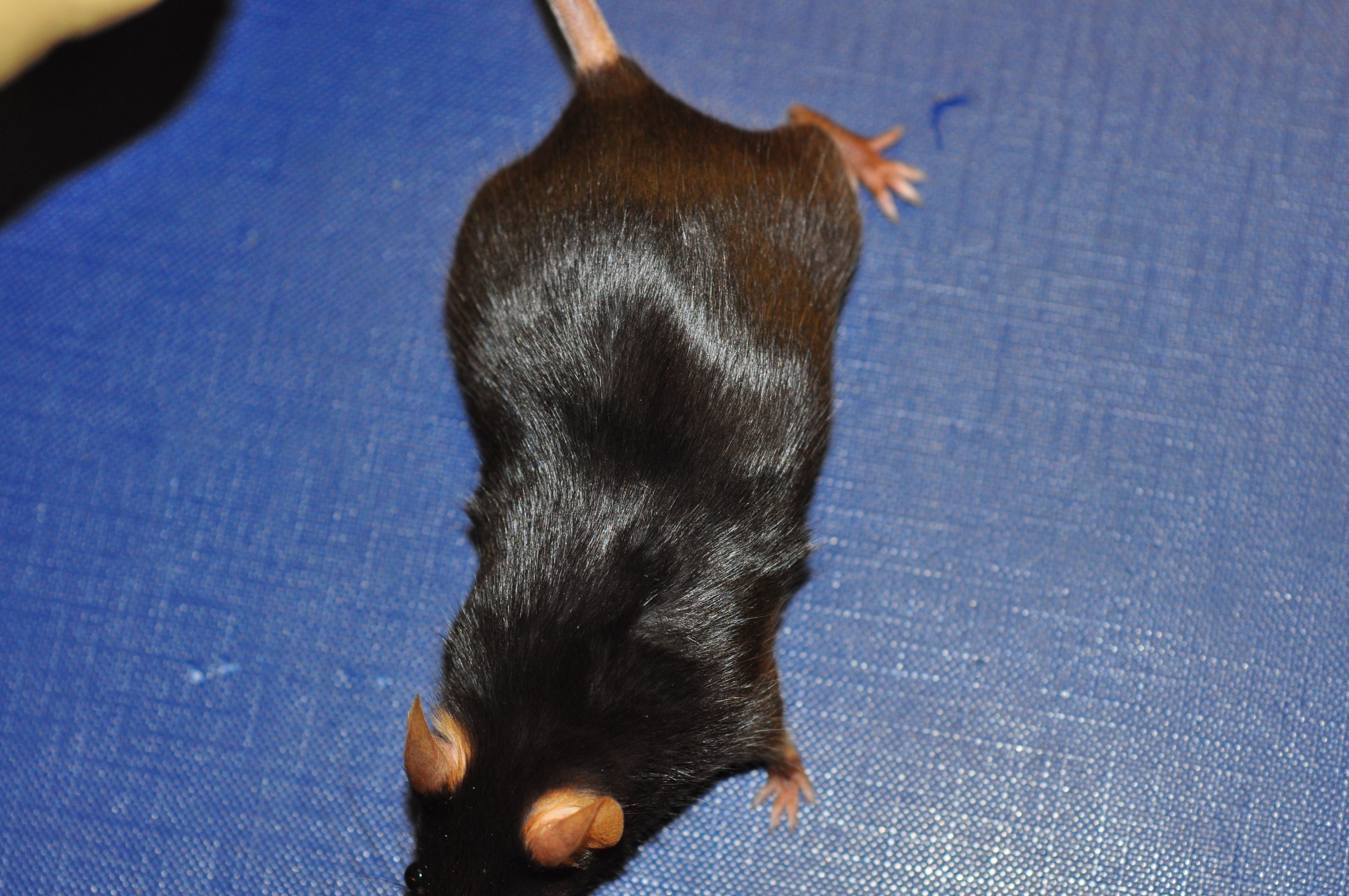

Supplement: Supplementary file 2 — Source data Fig. 1 [file 44321_2024_166_MOESM2_ESM.zip › EMM-2024-20141-V2_Source data for Figure 1/Figure 1E Image data/Fig 1E WT 5M.JPG]

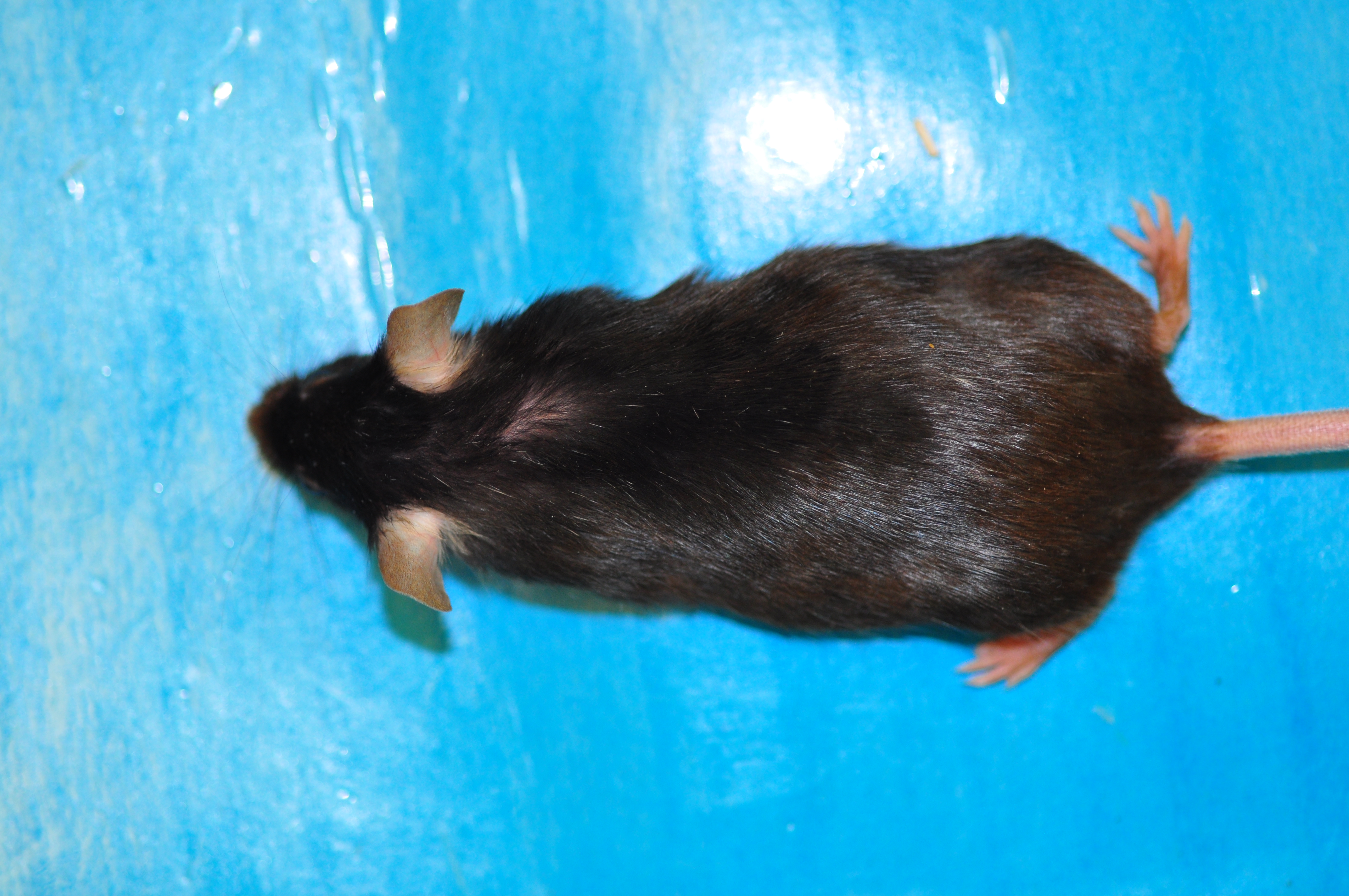

Supplement: Supplementary file 2 — Source data Fig. 1 [file 44321_2024_166_MOESM2_ESM.zip › EMM-2024-20141-V2_Source data for Figure 1/Figure 1E Image data/Fig 1E WT Abx.JPG]

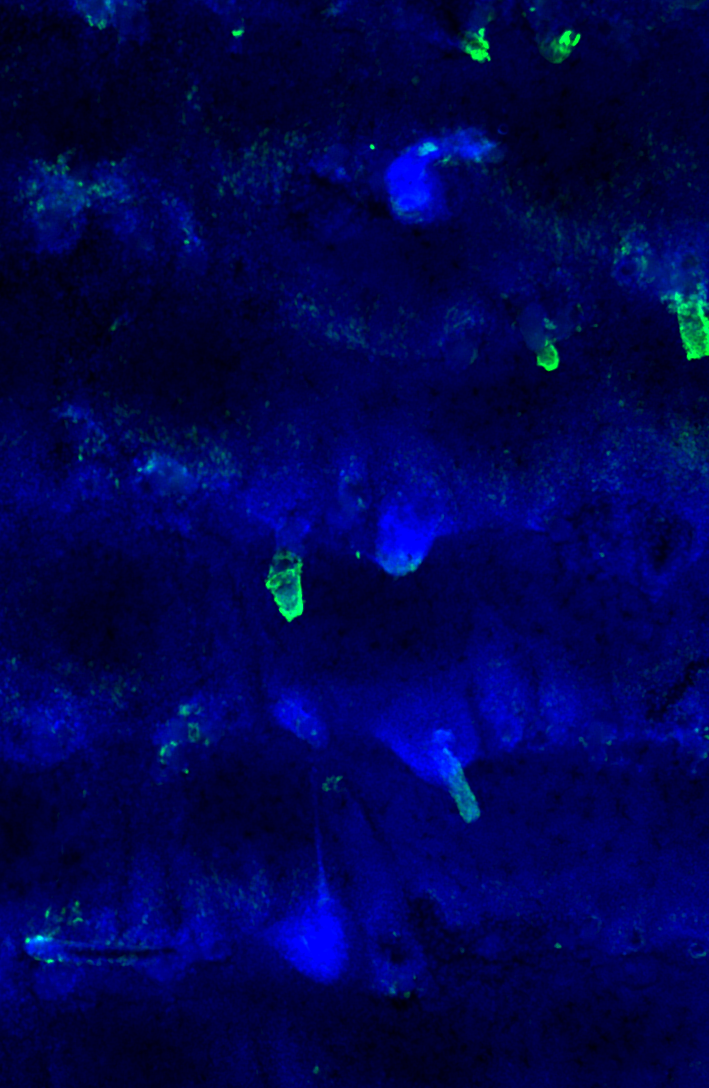

Supplement: Supplementary file 2 — Source data Fig. 1 [file 44321_2024_166_MOESM2_ESM.zip › EMM-2024-20141-V2_Source data for Figure 1/Figure 1I Image data/KO4 4x 7 25.tif]

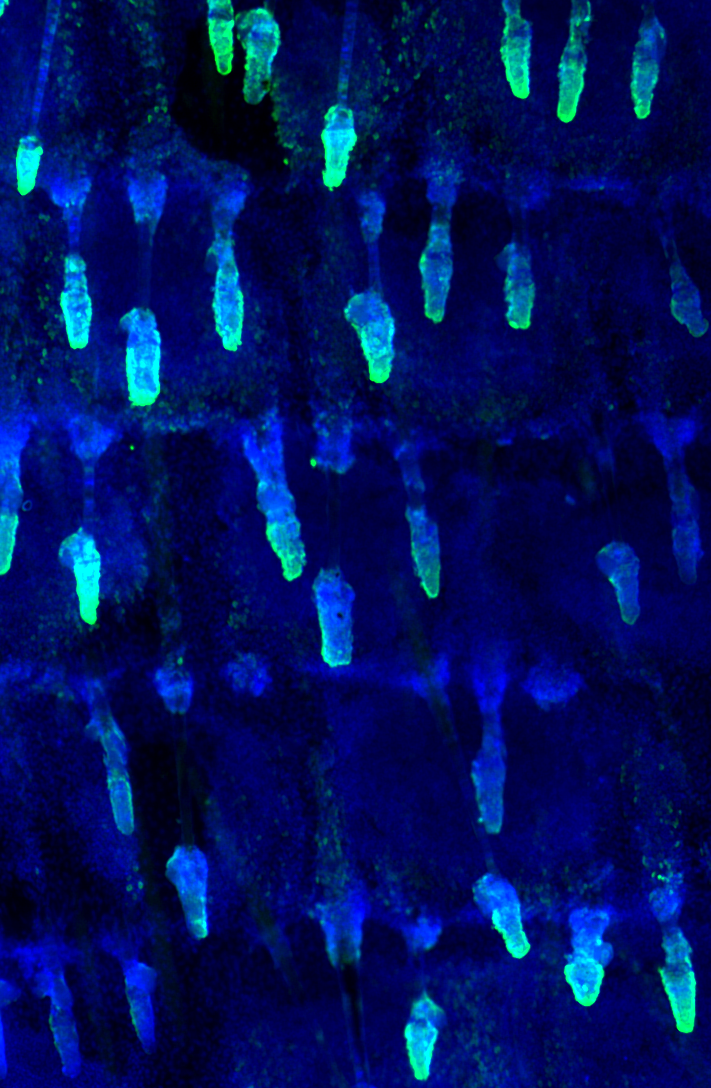

Supplement: Supplementary file 2 — Source data Fig. 1 [file 44321_2024_166_MOESM2_ESM.zip › EMM-2024-20141-V2_Source data for Figure 1/Figure 1I Image data/WT4 4x 1 25.tif]

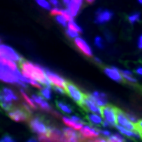

Supplement: Supplementary file 3 — Source data Fig. 2 [file 44321_2024_166_MOESM3_ESM.zip › EMM-2024-20141-V3_Source data for Figure 2/Figure 2D Image data/KO 20x 4 5x5 merged.tif]

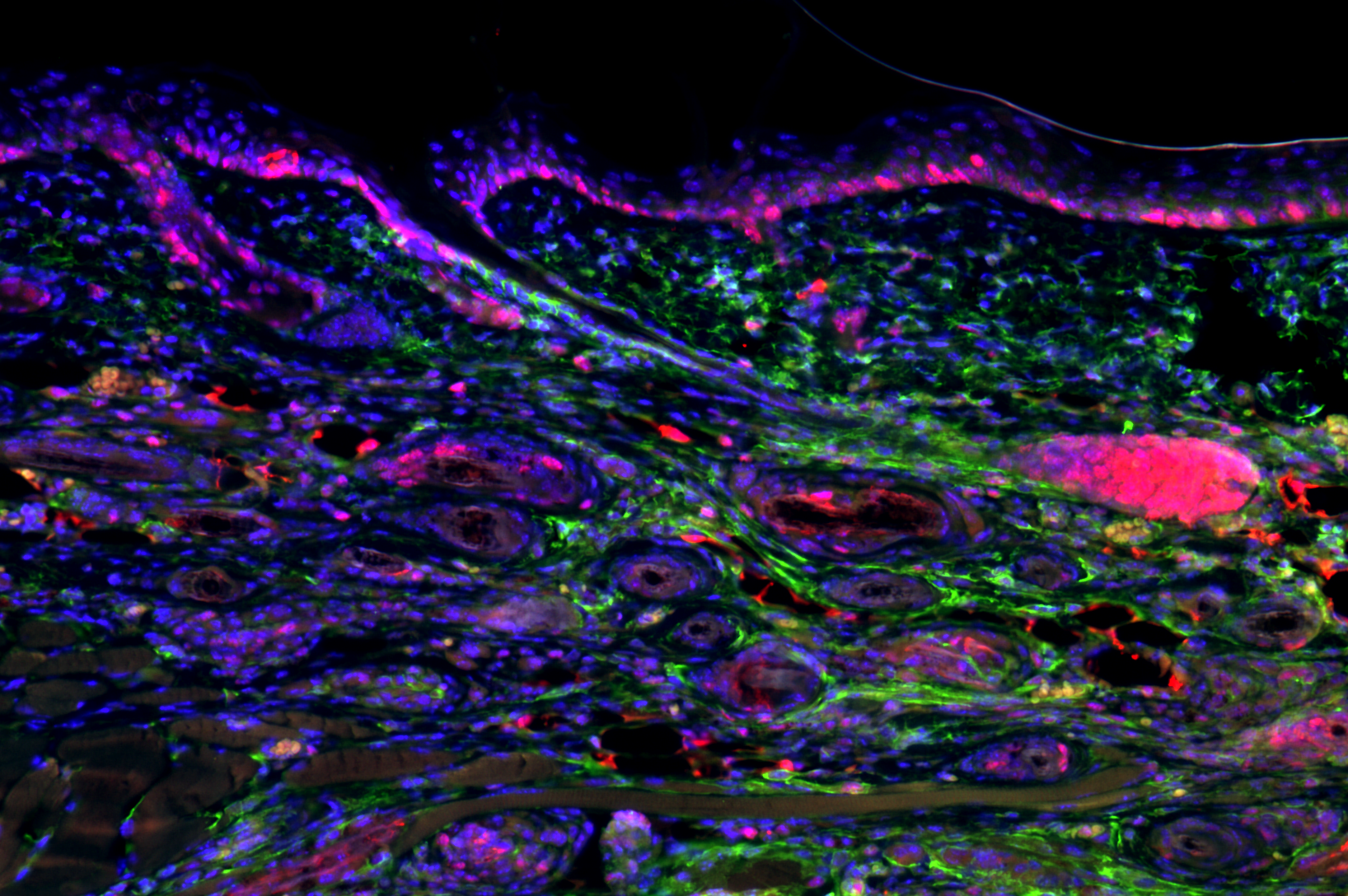

Supplement: Supplementary file 3 — Source data Fig. 2 [file 44321_2024_166_MOESM3_ESM.zip › EMM-2024-20141-V3_Source data for Figure 2/Figure 2D Image data/KO 20x 4.tif]

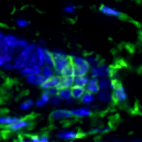

Supplement: Supplementary file 3 — Source data Fig. 2 [file 44321_2024_166_MOESM3_ESM.zip › EMM-2024-20141-V3_Source data for Figure 2/Figure 2D Image data/WT 20x 4 5x5 merged.tif]

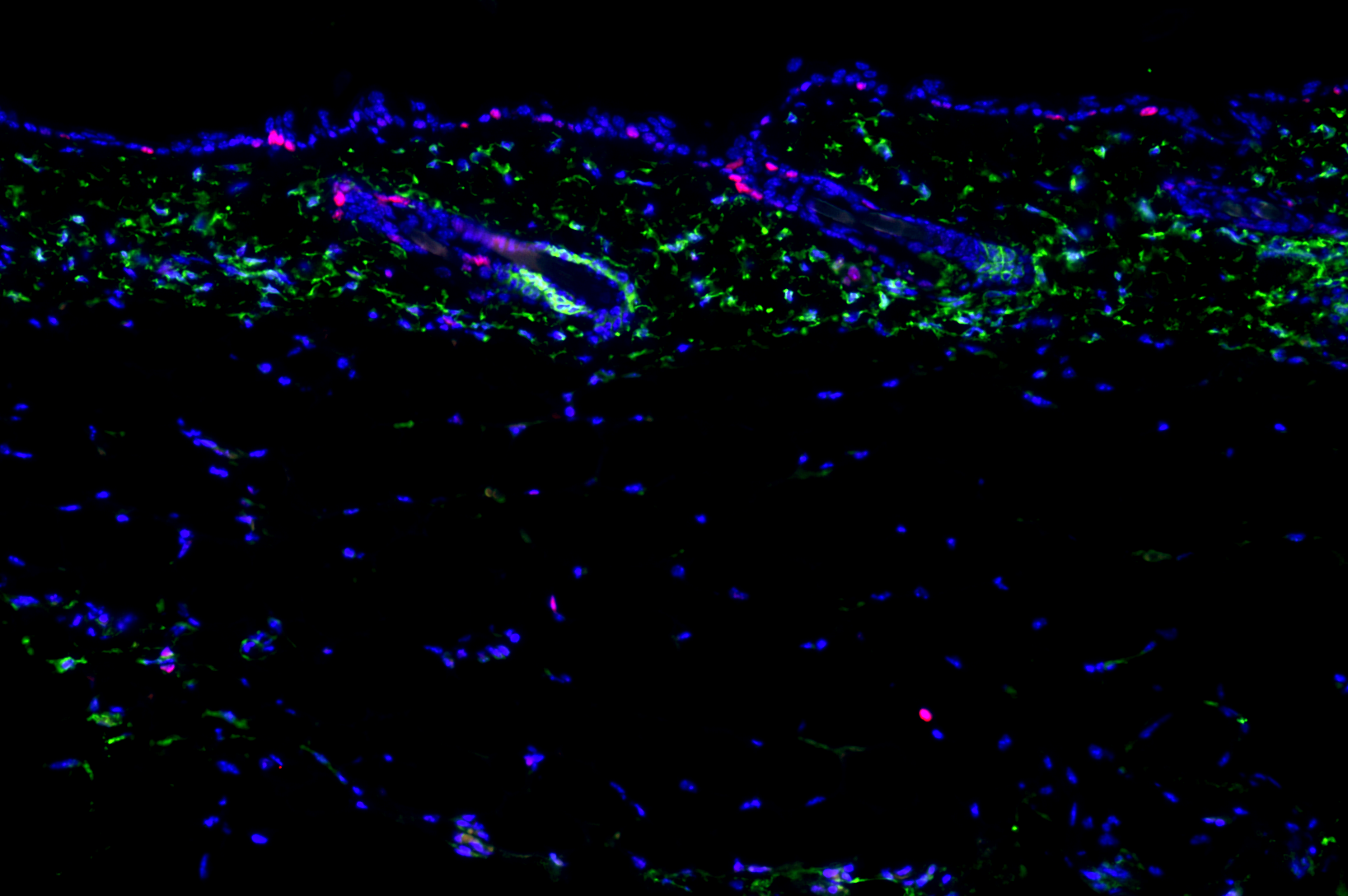

Supplement: Supplementary file 3 — Source data Fig. 2 [file 44321_2024_166_MOESM3_ESM.zip › EMM-2024-20141-V3_Source data for Figure 2/Figure 2D Image data/WT 20x 4.tif]

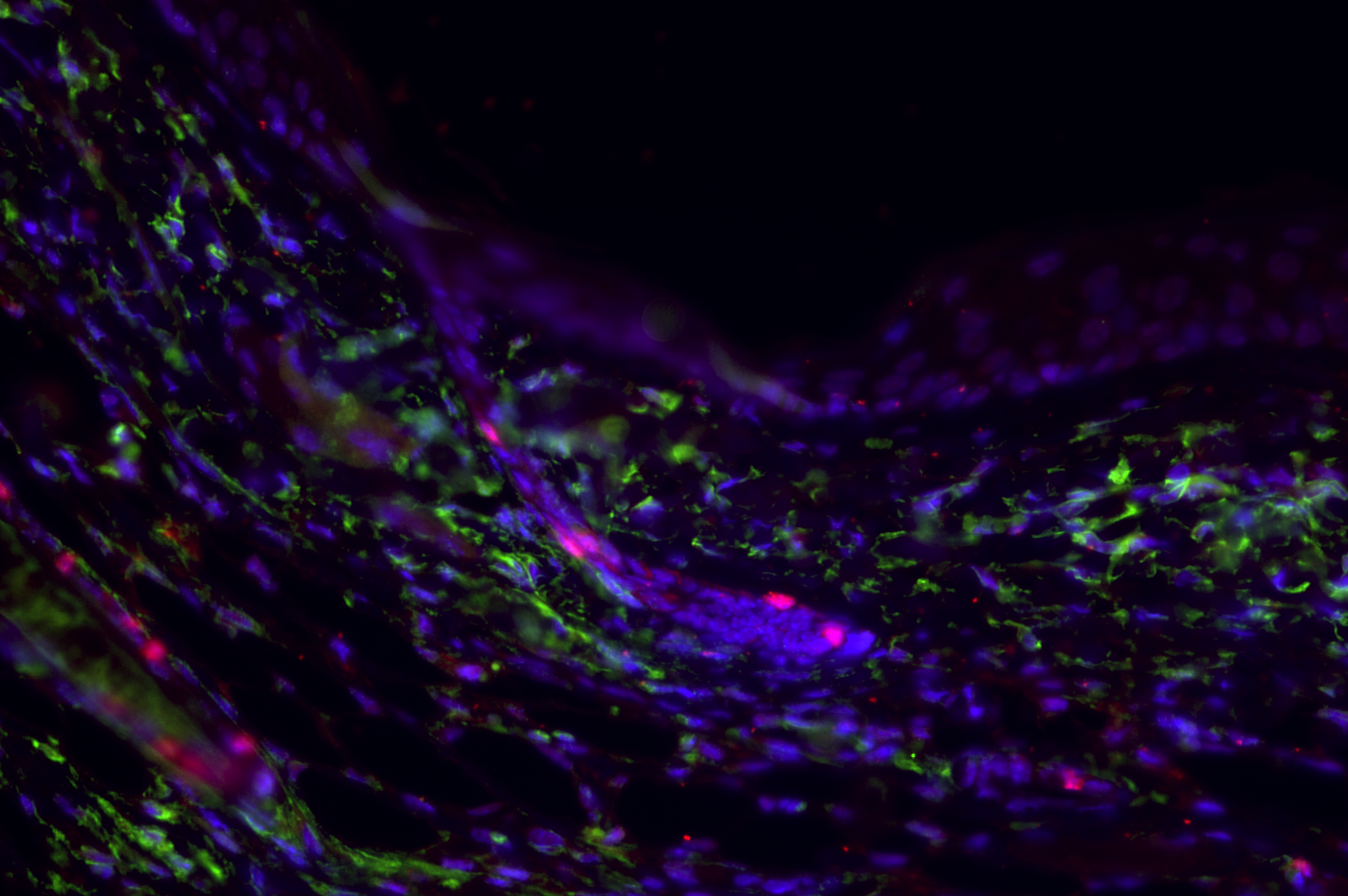

Supplement: Supplementary file 3 — Source data Fig. 2 [file 44321_2024_166_MOESM3_ESM.zip › EMM-2024-20141-V3_Source data for Figure 2/Figure 2F Image data/EGFRdEgr2 40x1_Casp8.tif]

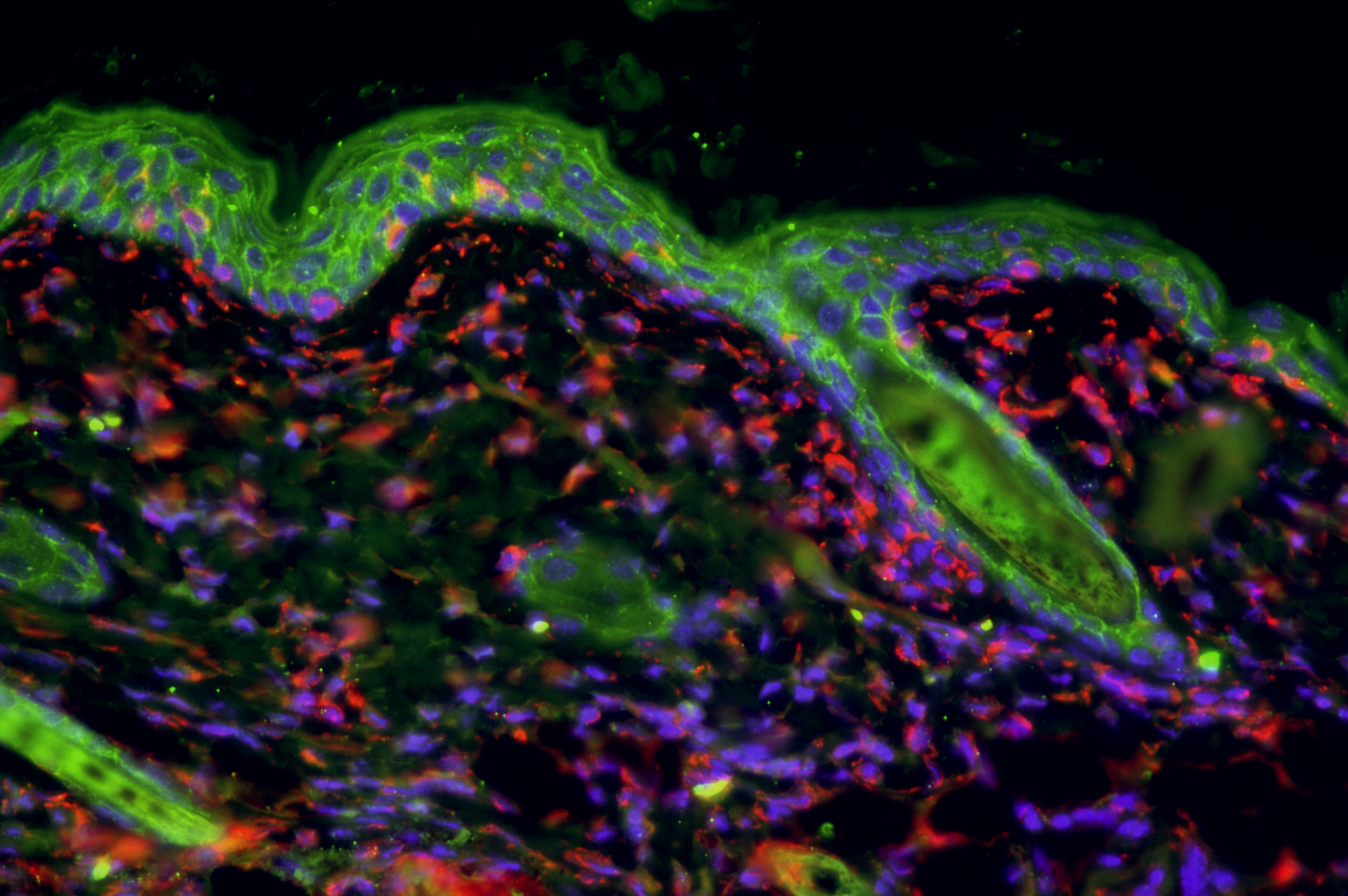

Supplement: Supplementary file 3 — Source data Fig. 2 [file 44321_2024_166_MOESM3_ESM.zip › EMM-2024-20141-V3_Source data for Figure 2/Figure 2F Image data/EGFRdEgr2 40x3_vimentin.tif]

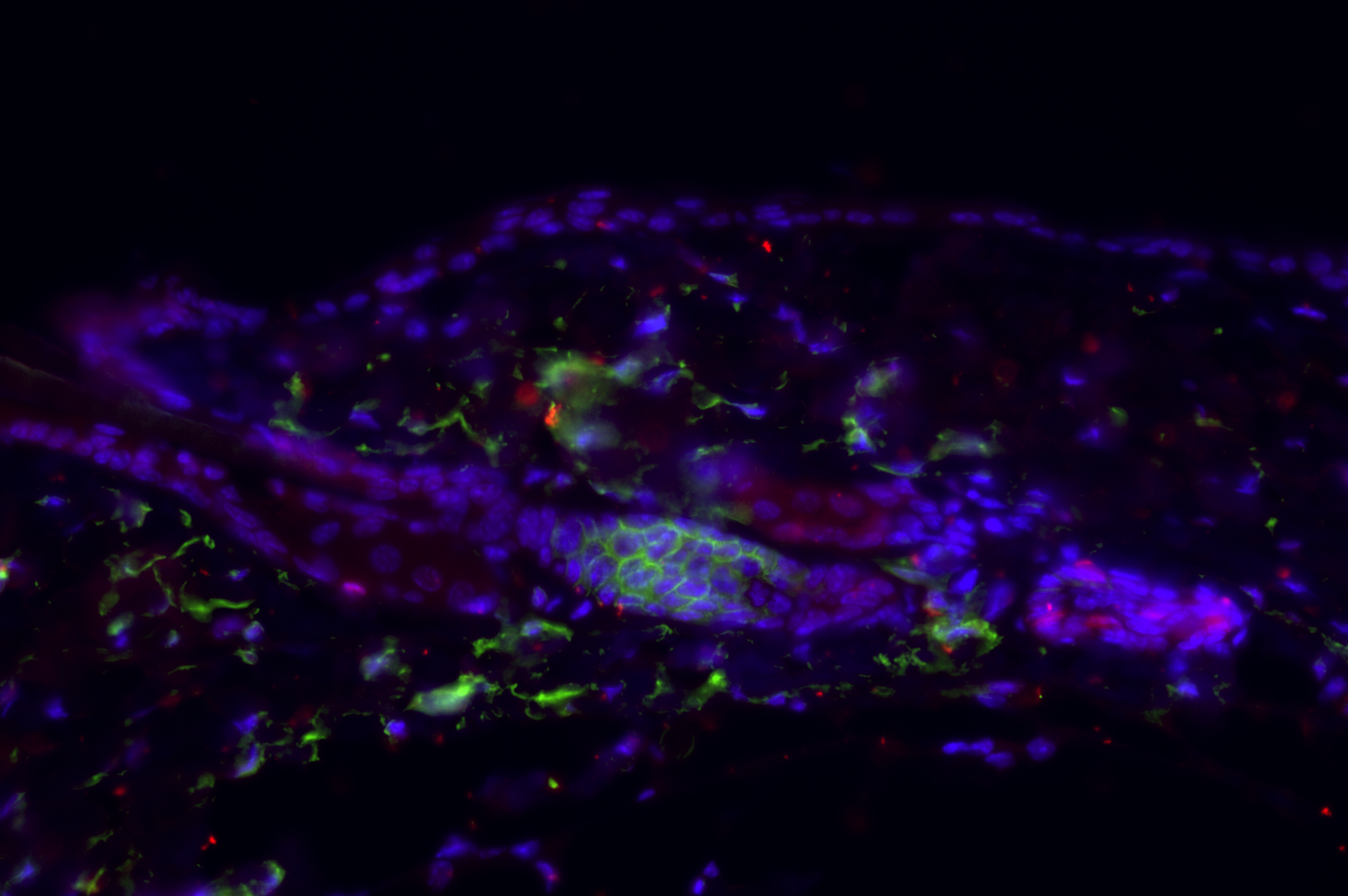

Supplement: Supplementary file 3 — Source data Fig. 2 [file 44321_2024_166_MOESM3_ESM.zip › EMM-2024-20141-V3_Source data for Figure 2/Figure 2F Image data/WT 40x1_Casp8.tif]

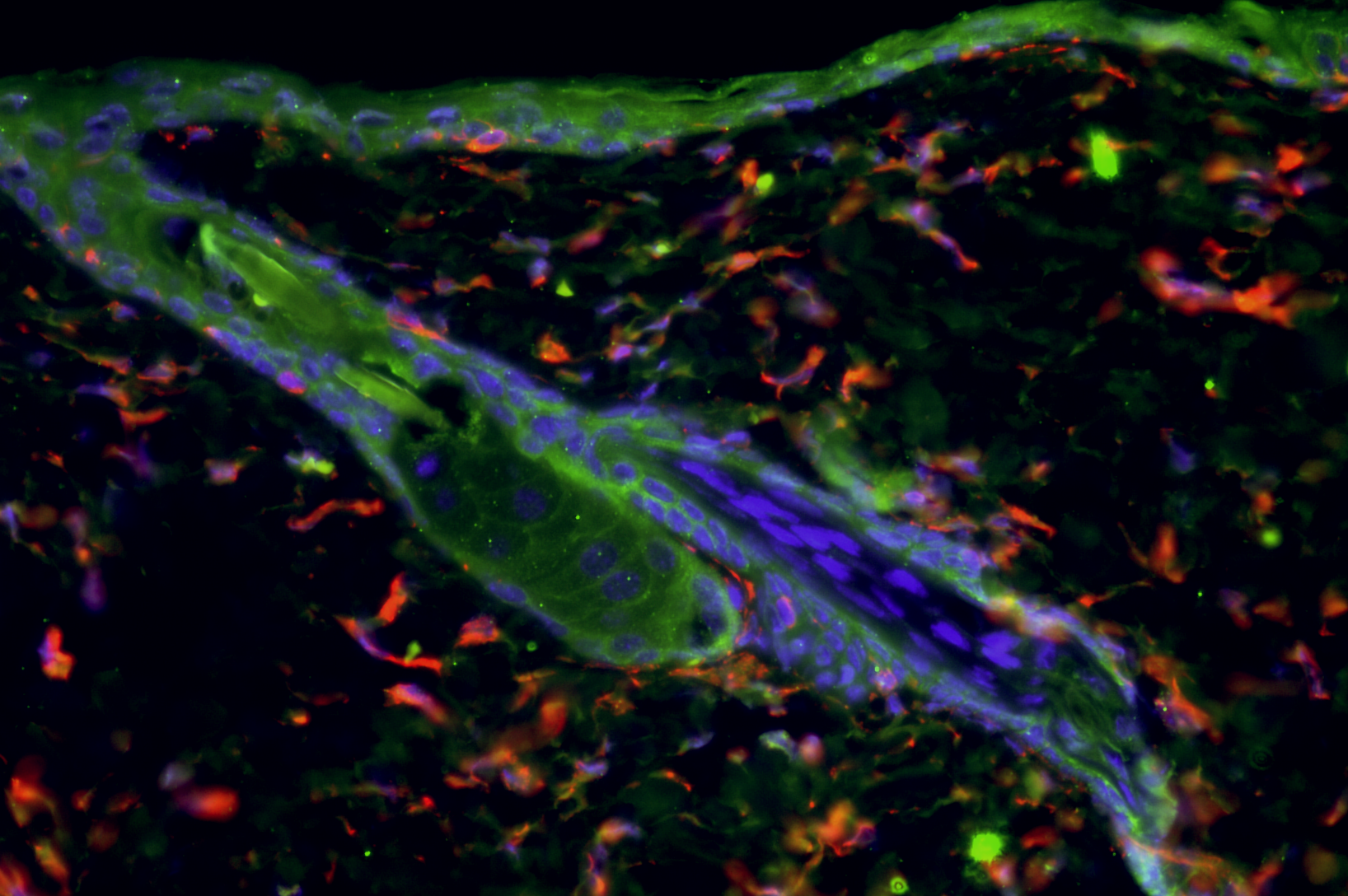

Supplement: Supplementary file 3 — Source data Fig. 2 [file 44321_2024_166_MOESM3_ESM.zip › EMM-2024-20141-V3_Source data for Figure 2/Figure 2F Image data/WT 40x1_vimentin.tif]

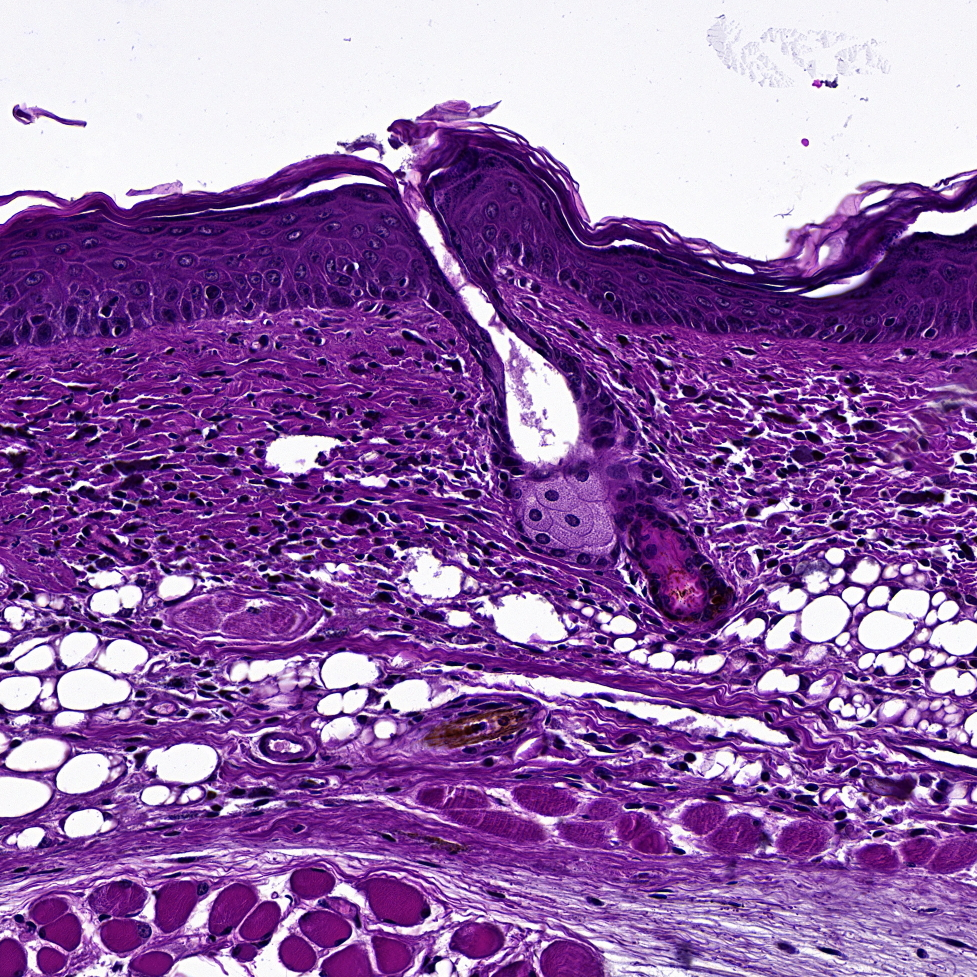

Supplement: Supplementary file 3 — Source data Fig. 2 [file 44321_2024_166_MOESM3_ESM.zip › EMM-2024-20141-V3_Source data for Figure 2/Figure 2G Image data/EGFRdEgr2 Follicular plugging hyperkeratosis.tif]

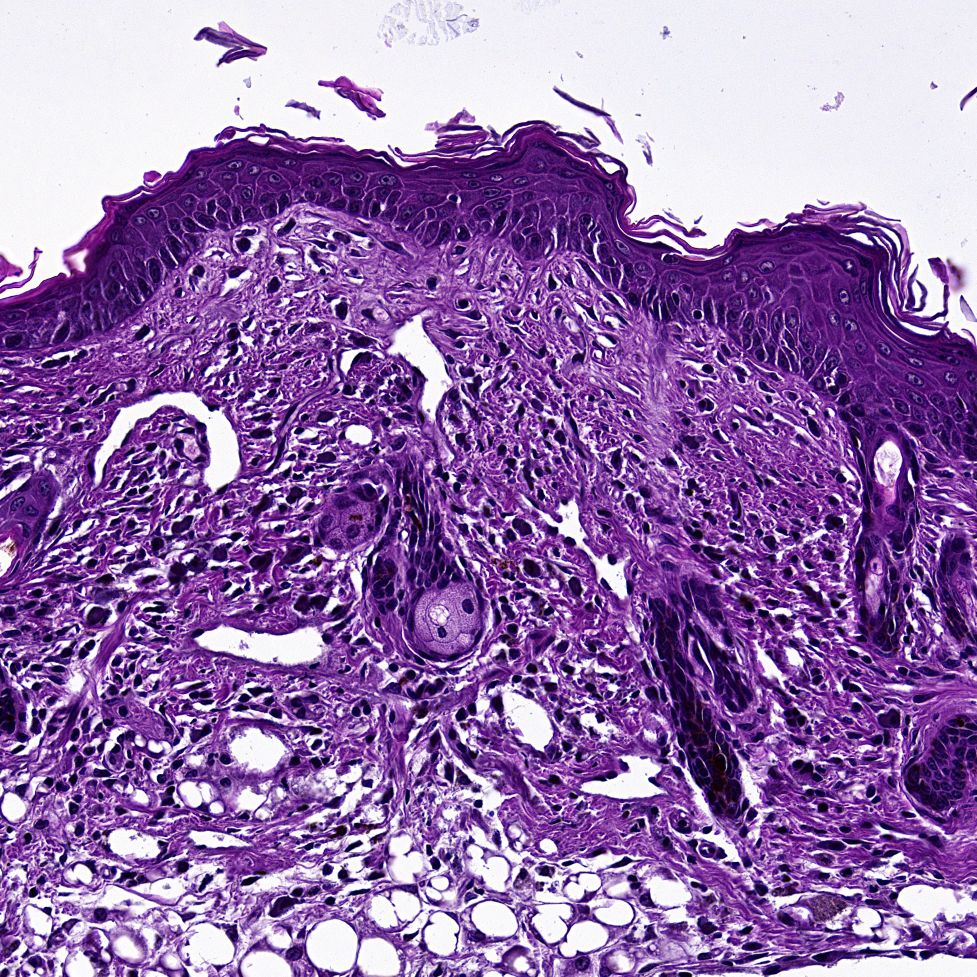

Supplement: Supplementary file 3 — Source data Fig. 2 [file 44321_2024_166_MOESM3_ESM.zip › EMM-2024-20141-V3_Source data for Figure 2/Figure 2G Image data/EGFRdEgr2 Follicular scarring, fibrous connective tissue.tif]

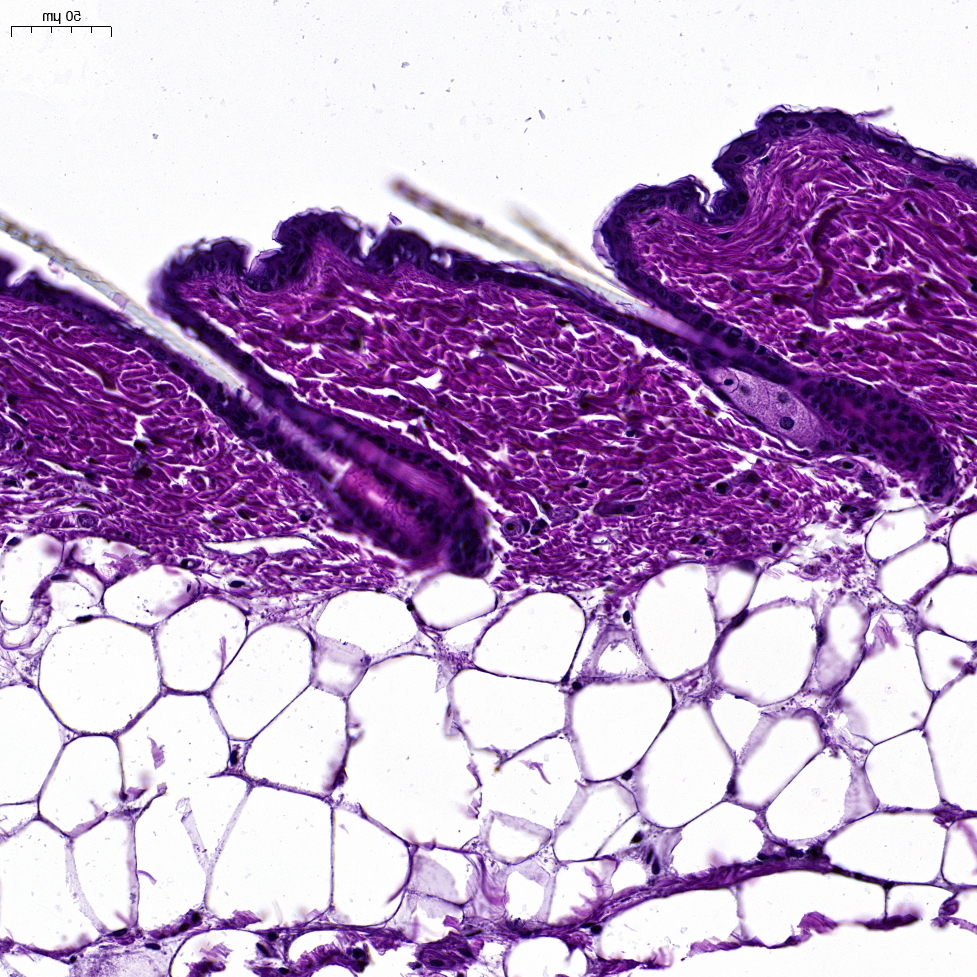

Supplement: Supplementary file 3 — Source data Fig. 2 [file 44321_2024_166_MOESM3_ESM.zip › EMM-2024-20141-V3_Source data for Figure 2/Figure 2G Image data/WT 5M Fig.tif]

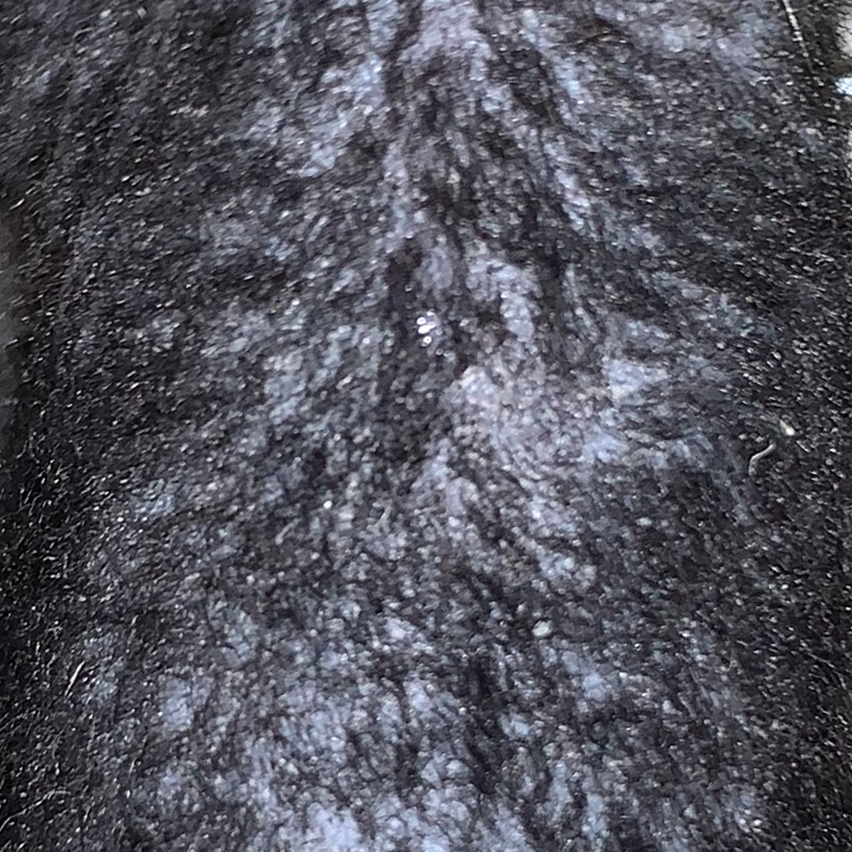

Supplement: Supplementary file 4 — Source data Fig. 3 [file 44321_2024_166_MOESM4_ESM.zip › EMM-2024-20141-V3_Source data for Figure 3/Figure 3F Image data/EGFR STAT1DEgr2.tif]

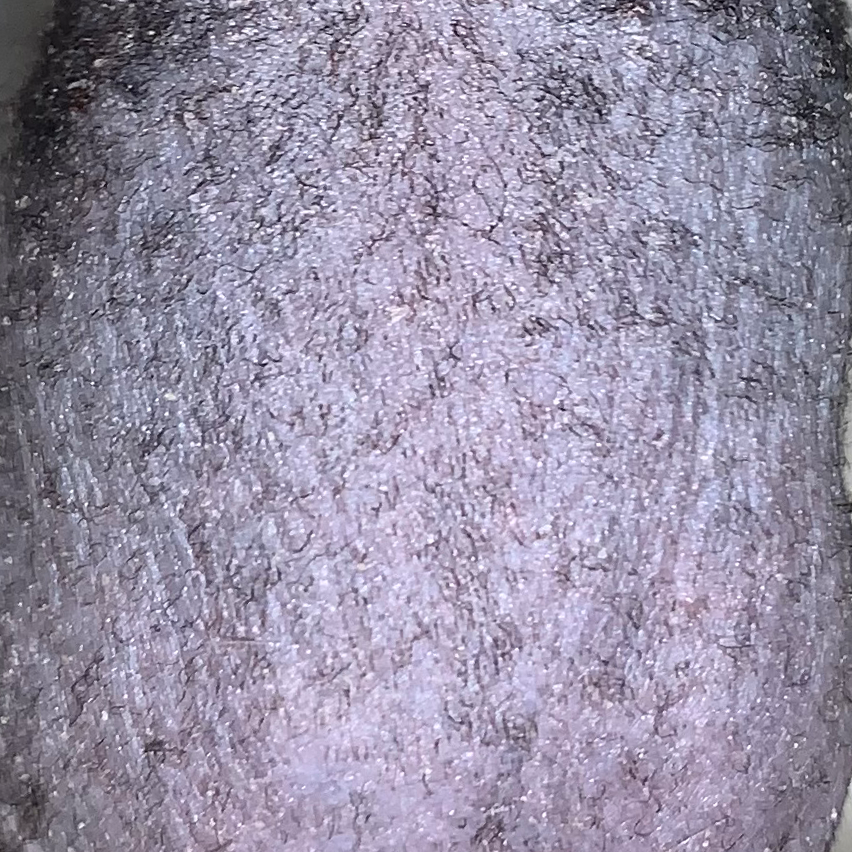

Supplement: Supplementary file 4 — Source data Fig. 3 [file 44321_2024_166_MOESM4_ESM.zip › EMM-2024-20141-V3_Source data for Figure 3/Figure 3F Image data/EGFRDEgr2.tif]

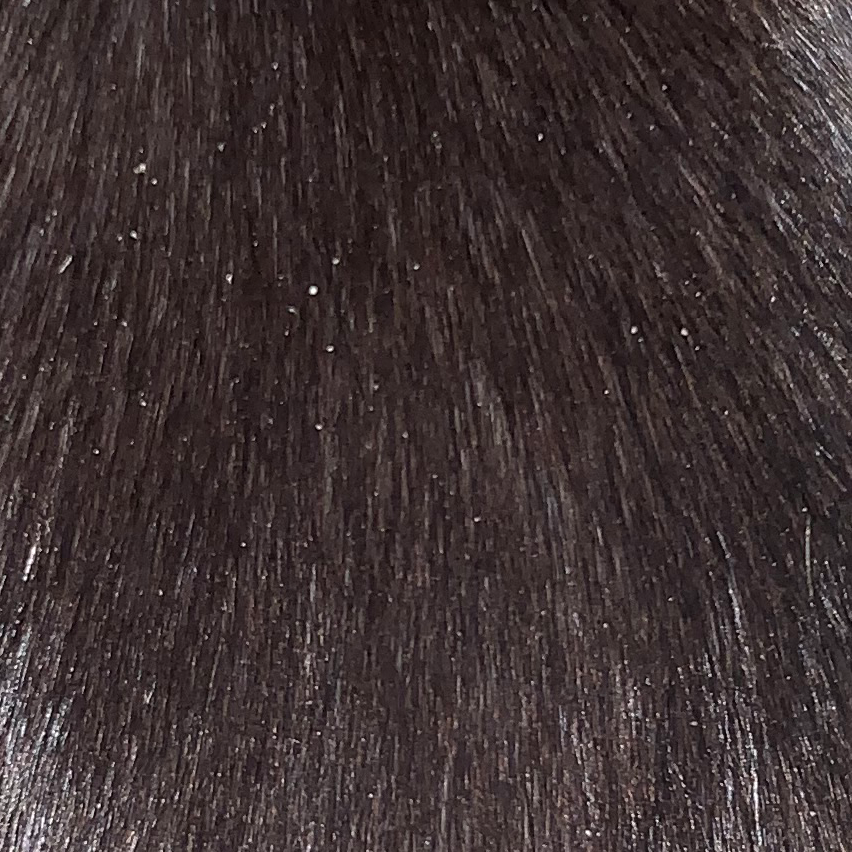

Supplement: Supplementary file 4 — Source data Fig. 3 [file 44321_2024_166_MOESM4_ESM.zip › EMM-2024-20141-V3_Source data for Figure 3/Figure 3F Image data/WT.tif]

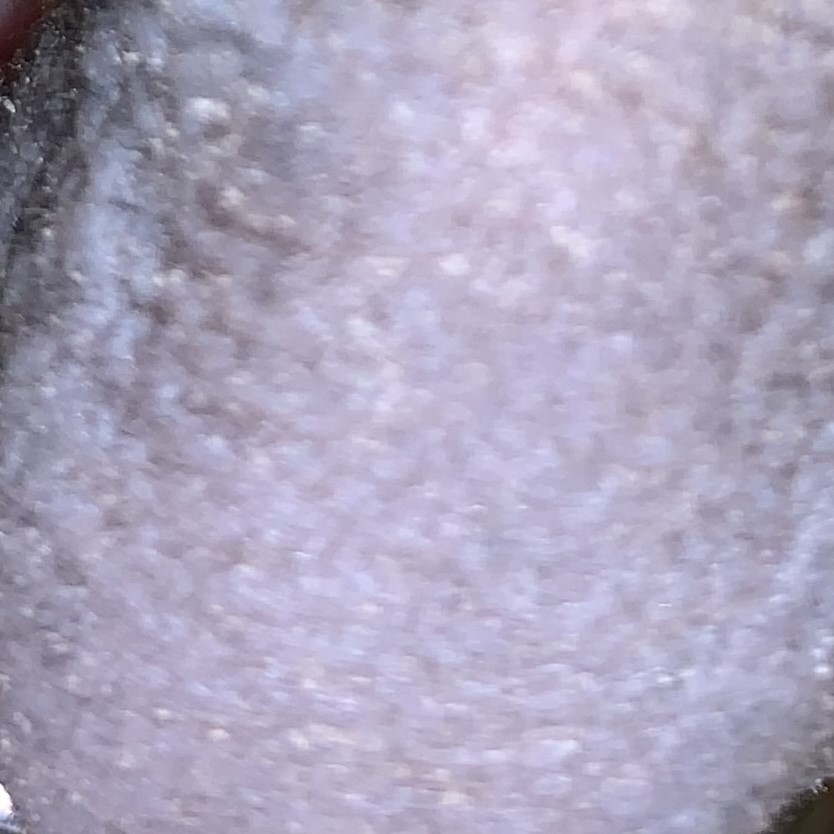

Supplement: Supplementary file 5 — Source data Fig. 5 [file 44321_2024_166_MOESM5_ESM.zip › EMM-2024-20141-V3_Source data for Figure 5/Figure 5B Image data/DMSO d0.jpeg]

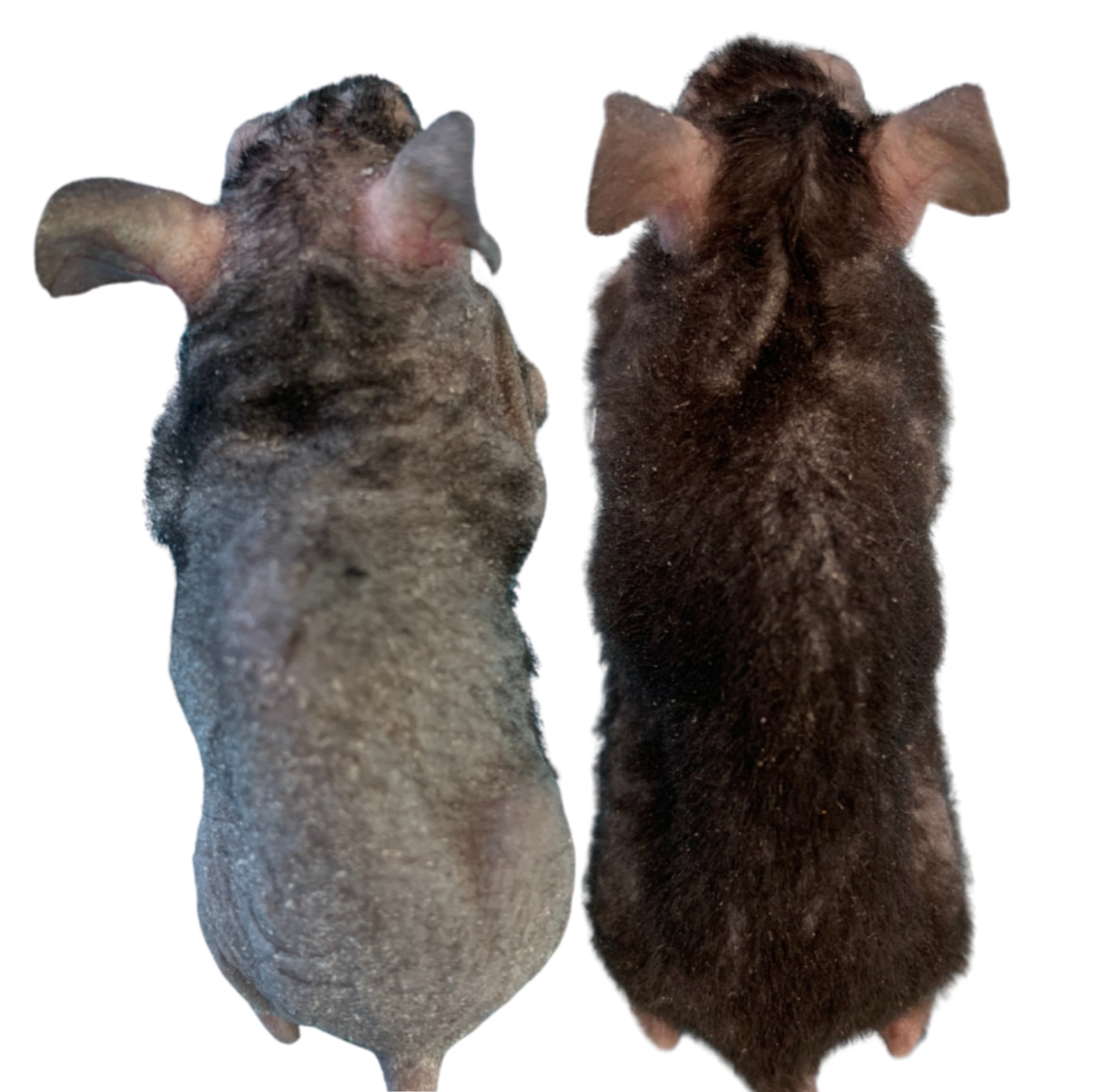

Supplement: Supplementary file 5 — Source data Fig. 5 [file 44321_2024_166_MOESM5_ESM.zip › EMM-2024-20141-V3_Source data for Figure 5/Figure 5B Image data/EGR2 D28 Ruxo mouse.png]

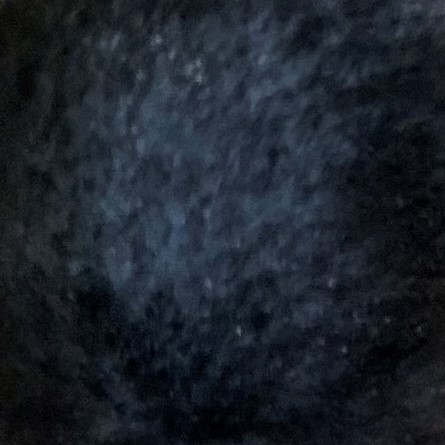

Supplement: Supplementary file 5 — Source data Fig. 5 [file 44321_2024_166_MOESM5_ESM.zip › EMM-2024-20141-V3_Source data for Figure 5/Figure 5B Image data/IMG_4592_Ruxod14 cut.jpg]

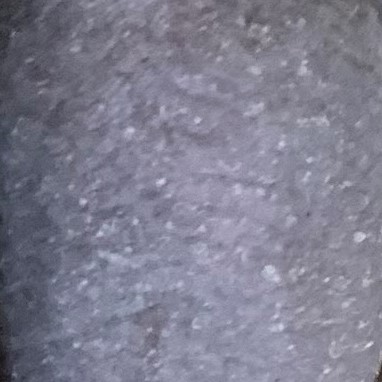

Supplement: Supplementary file 5 — Source data Fig. 5 [file 44321_2024_166_MOESM5_ESM.zip › EMM-2024-20141-V3_Source data for Figure 5/Figure 5B Image data/IMG_4596 DMSOd14 cut.jpg]

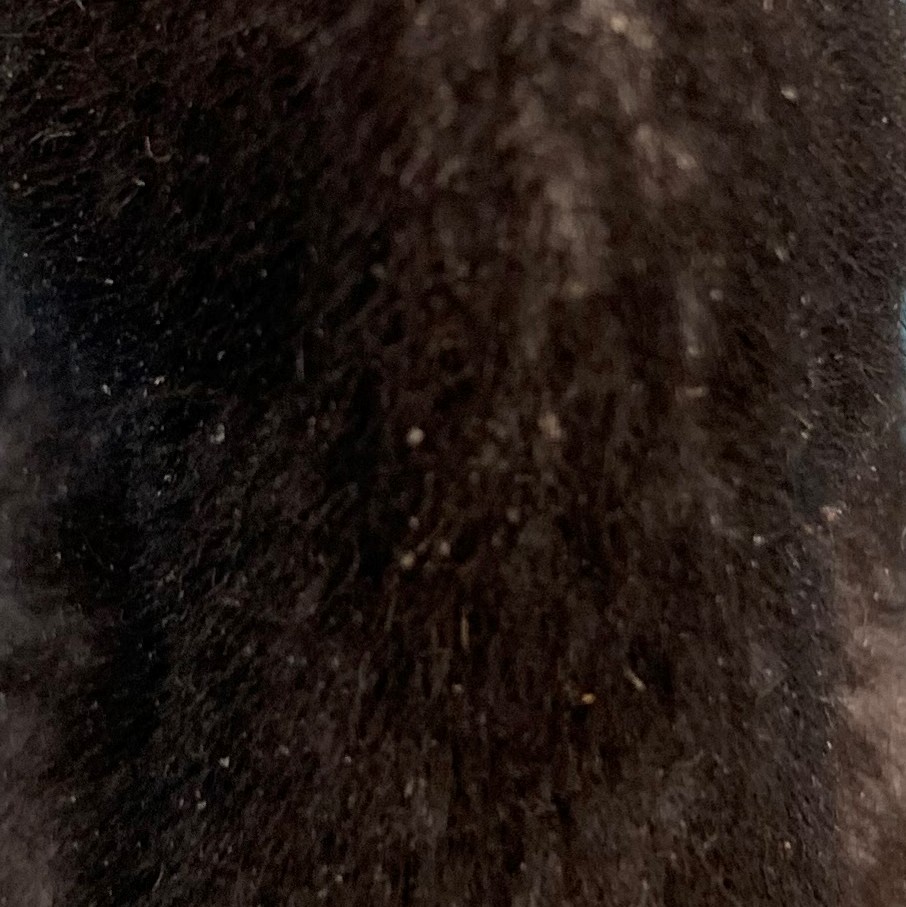

Supplement: Supplementary file 5 — Source data Fig. 5 [file 44321_2024_166_MOESM5_ESM.zip › EMM-2024-20141-V3_Source data for Figure 5/Figure 5B Image data/IMG_4690_Ruxod28 cut.jpg]

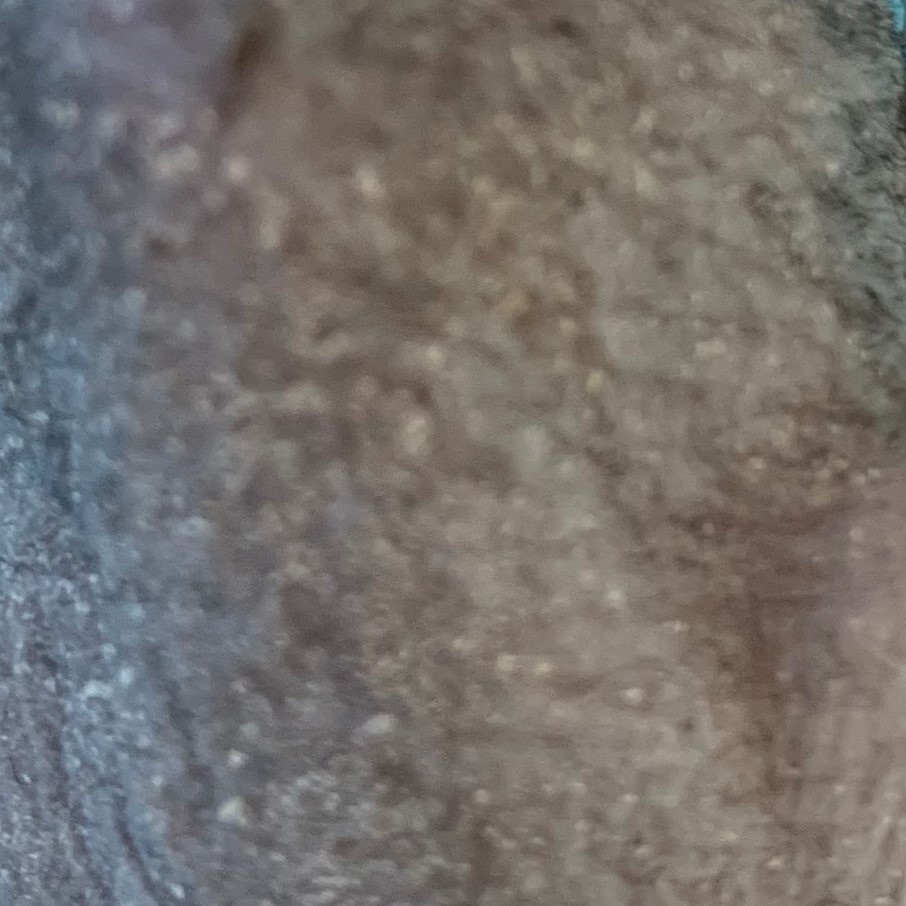

Supplement: Supplementary file 5 — Source data Fig. 5 [file 44321_2024_166_MOESM5_ESM.zip › EMM-2024-20141-V3_Source data for Figure 5/Figure 5B Image data/IMG_4704_DMSOd28 cut.jpg]

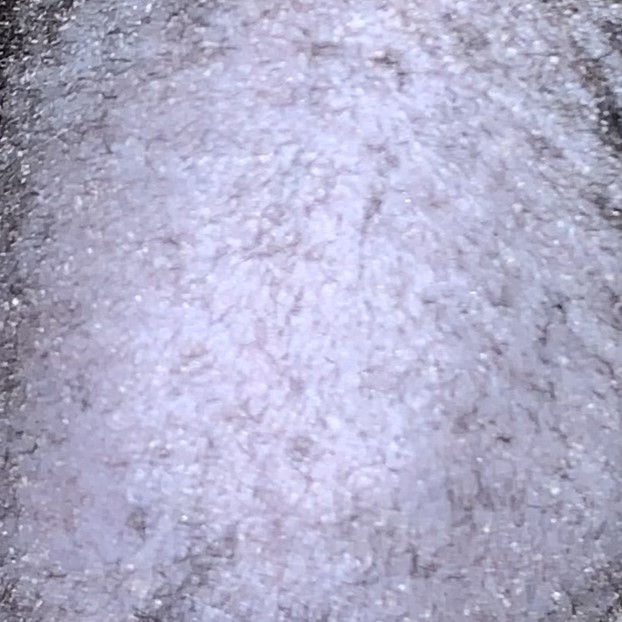

Supplement: Supplementary file 5 — Source data Fig. 5 [file 44321_2024_166_MOESM5_ESM.zip › EMM-2024-20141-V3_Source data for Figure 5/Figure 5B Image data/RUXO d0.jpeg]

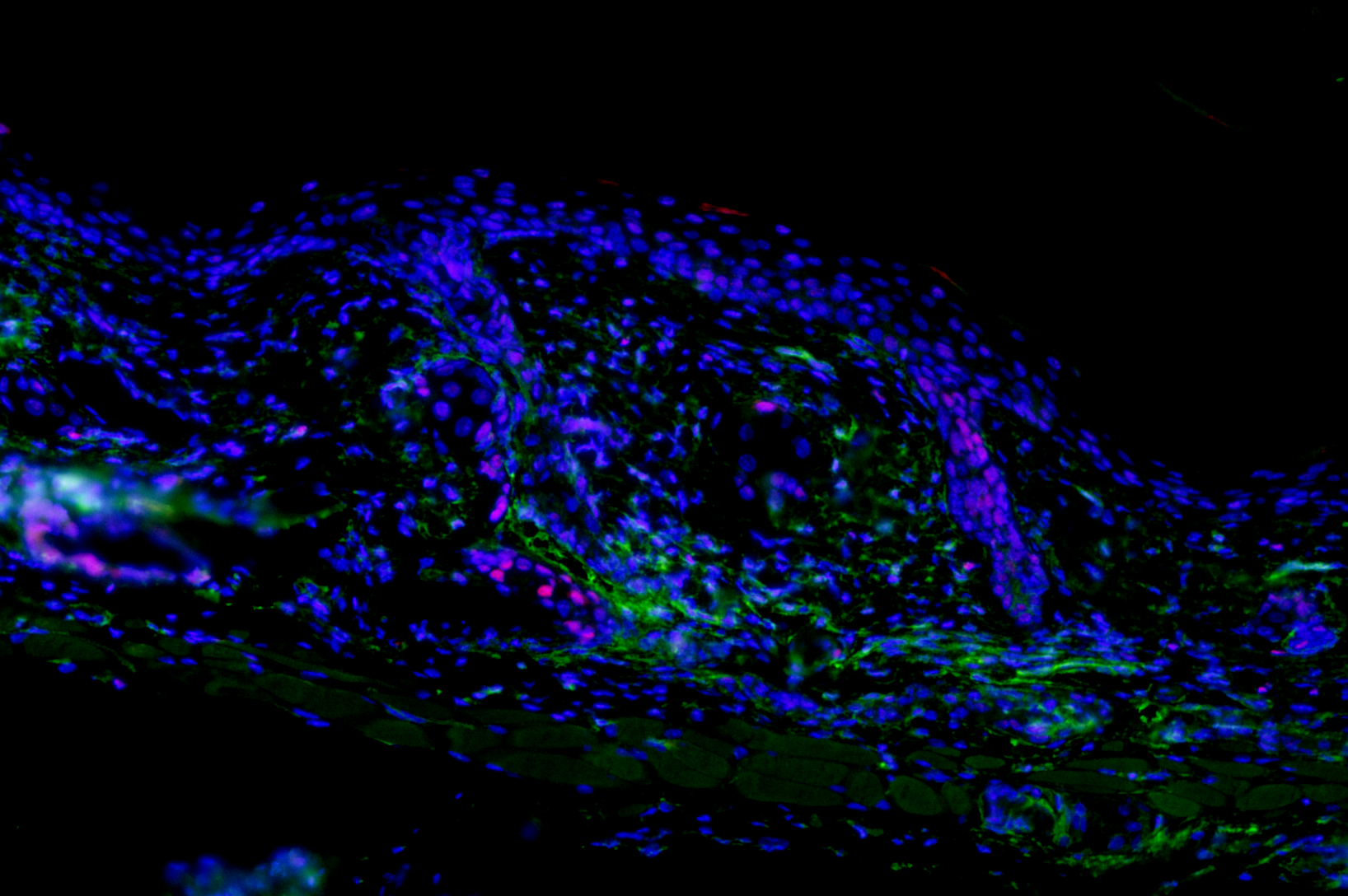

Supplement: Supplementary file 5 — Source data Fig. 5 [file 44321_2024_166_MOESM5_ESM.zip › EMM-2024-20141-V3_Source data for Figure 5/Figure 5D Image data/EGFRDEgr2 Ctrl/20x4 af488 300ms copy.tif]

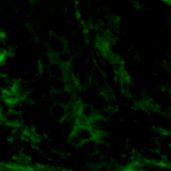

Supplement: Supplementary file 5 — Source data Fig. 5 [file 44321_2024_166_MOESM5_ESM.zip › EMM-2024-20141-V3_Source data for Figure 5/Figure 5D Image data/EGFRDEgr2 Ctrl/20x4 af488 300ms cut green.tif]

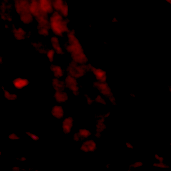

Supplement: Supplementary file 5 — Source data Fig. 5 [file 44321_2024_166_MOESM5_ESM.zip › EMM-2024-20141-V3_Source data for Figure 5/Figure 5D Image data/EGFRDEgr2 Ctrl/20x4 af488 300ms cut red.tif]

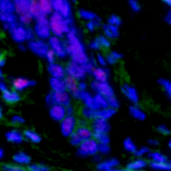

Supplement: Supplementary file 5 — Source data Fig. 5 [file 44321_2024_166_MOESM5_ESM.zip › EMM-2024-20141-V3_Source data for Figure 5/Figure 5D Image data/EGFRDEgr2 Ctrl/20x4 af488 300ms cut.tif]

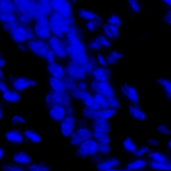

Supplement: Supplementary file 5 — Source data Fig. 5 [file 44321_2024_166_MOESM5_ESM.zip › EMM-2024-20141-V3_Source data for Figure 5/Figure 5D Image data/EGFRDEgr2 Ctrl/20x4 af488 300ms cutblue.tif]

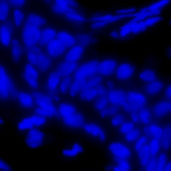

Supplement: Supplementary file 5 — Source data Fig. 5 [file 44321_2024_166_MOESM5_ESM.zip › EMM-2024-20141-V3_Source data for Figure 5/Figure 5D Image data/EGFRDEgr2 Ruxo/20x3 copy cut blue.tif]

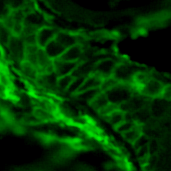

Supplement: Supplementary file 5 — Source data Fig. 5 [file 44321_2024_166_MOESM5_ESM.zip › EMM-2024-20141-V3_Source data for Figure 5/Figure 5D Image data/EGFRDEgr2 Ruxo/20x3 copy cut green.tif]

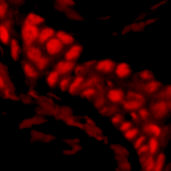

Supplement: Supplementary file 5 — Source data Fig. 5 [file 44321_2024_166_MOESM5_ESM.zip › EMM-2024-20141-V3_Source data for Figure 5/Figure 5D Image data/EGFRDEgr2 Ruxo/20x3 copy cut red.tif]

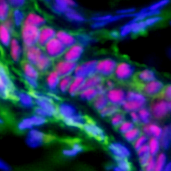

Supplement: Supplementary file 5 — Source data Fig. 5 [file 44321_2024_166_MOESM5_ESM.zip › EMM-2024-20141-V3_Source data for Figure 5/Figure 5D Image data/EGFRDEgr2 Ruxo/20x3 copy cut.tif]

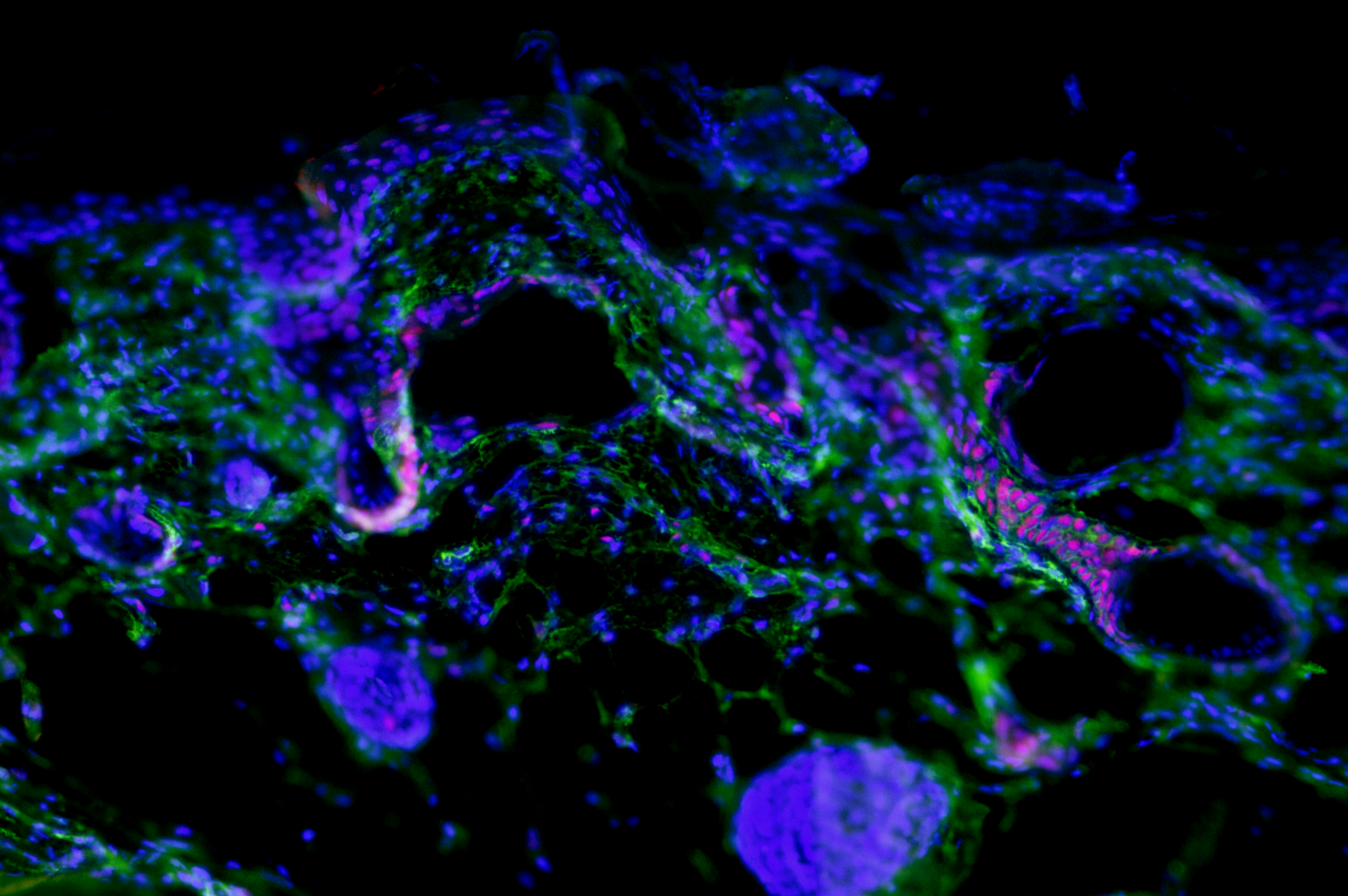

Supplement: Supplementary file 5 — Source data Fig. 5 [file 44321_2024_166_MOESM5_ESM.zip › EMM-2024-20141-V3_Source data for Figure 5/Figure 5D Image data/EGFRDEgr2 Ruxo/20x3 copy.tif]

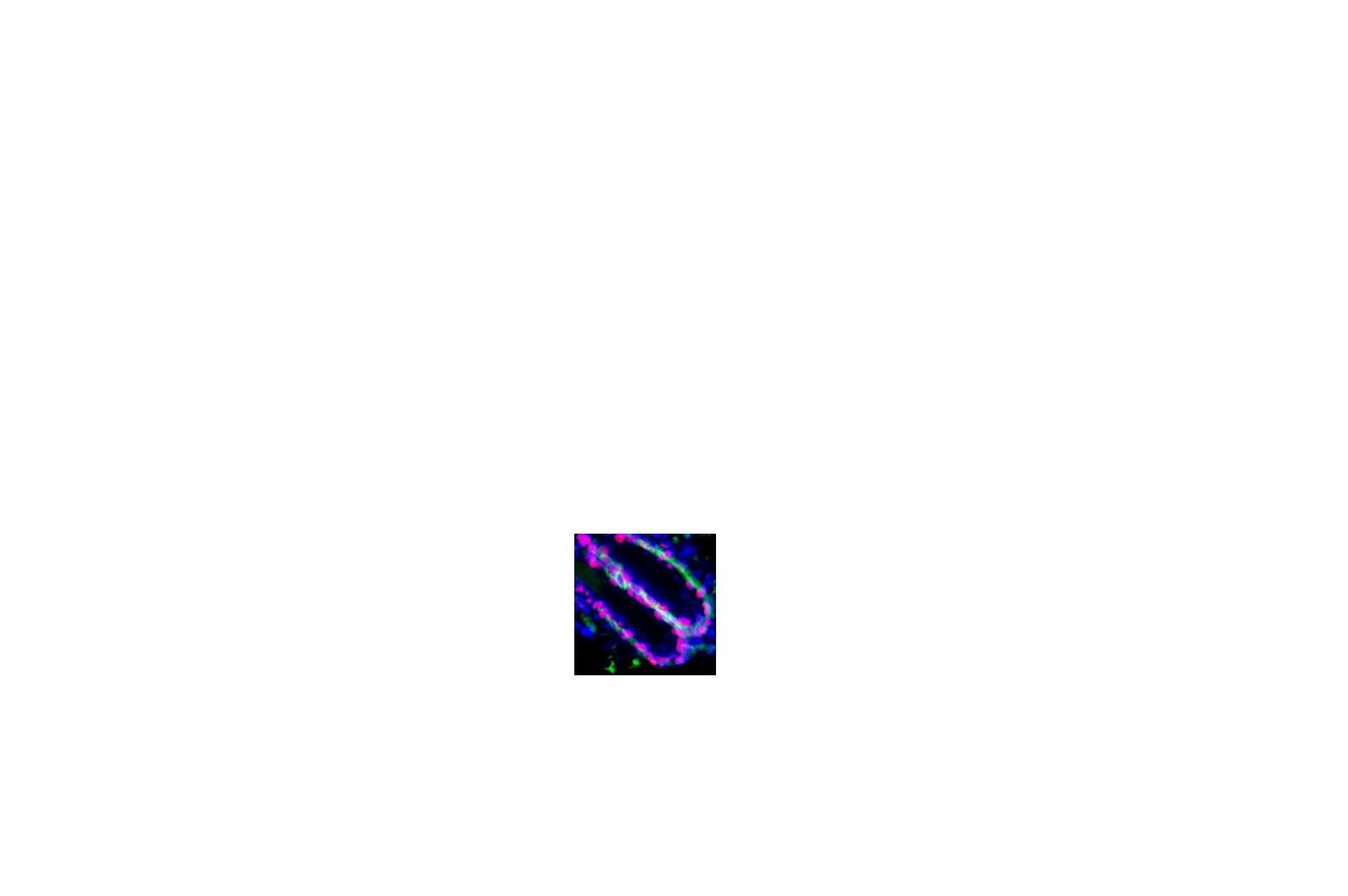

Supplement: Supplementary file 5 — Source data Fig. 5 [file 44321_2024_166_MOESM5_ESM.zip › EMM-2024-20141-V3_Source data for Figure 5/Figure 5D Image data/WT Ctrl/20x1 copy.tif]

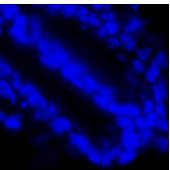

Supplement: Supplementary file 5 — Source data Fig. 5 [file 44321_2024_166_MOESM5_ESM.zip › EMM-2024-20141-V3_Source data for Figure 5/Figure 5D Image data/WT Ctrl/20x1 cut blue.tif]

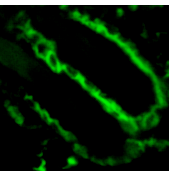

Supplement: Supplementary file 5 — Source data Fig. 5 [file 44321_2024_166_MOESM5_ESM.zip › EMM-2024-20141-V3_Source data for Figure 5/Figure 5D Image data/WT Ctrl/20x1 cut green.tif]

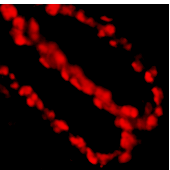

Supplement: Supplementary file 5 — Source data Fig. 5 [file 44321_2024_166_MOESM5_ESM.zip › EMM-2024-20141-V3_Source data for Figure 5/Figure 5D Image data/WT Ctrl/20x1 cut red.tif]

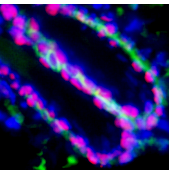

Supplement: Supplementary file 5 — Source data Fig. 5 [file 44321_2024_166_MOESM5_ESM.zip › EMM-2024-20141-V3_Source data for Figure 5/Figure 5D Image data/WT Ctrl/20x1 cut.tif]

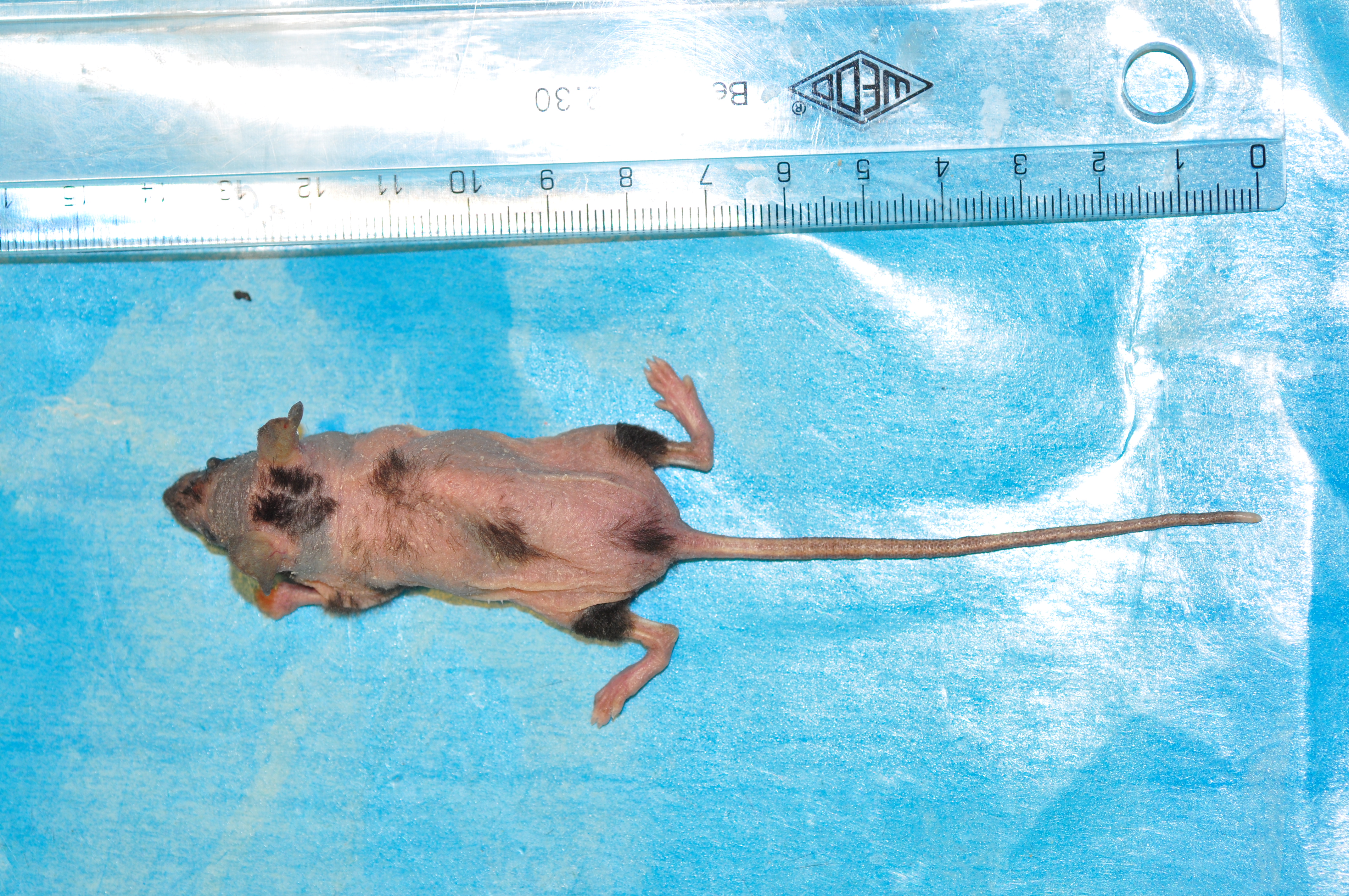

Supplement: Supplementary file 5 — Source data Fig. 5 [file 44321_2024_166_MOESM5_ESM.zip › EMM-2024-20141-V3_Source data for Figure 5/Figure 5K Image data/Fig 5K EGFRDep.JPG]

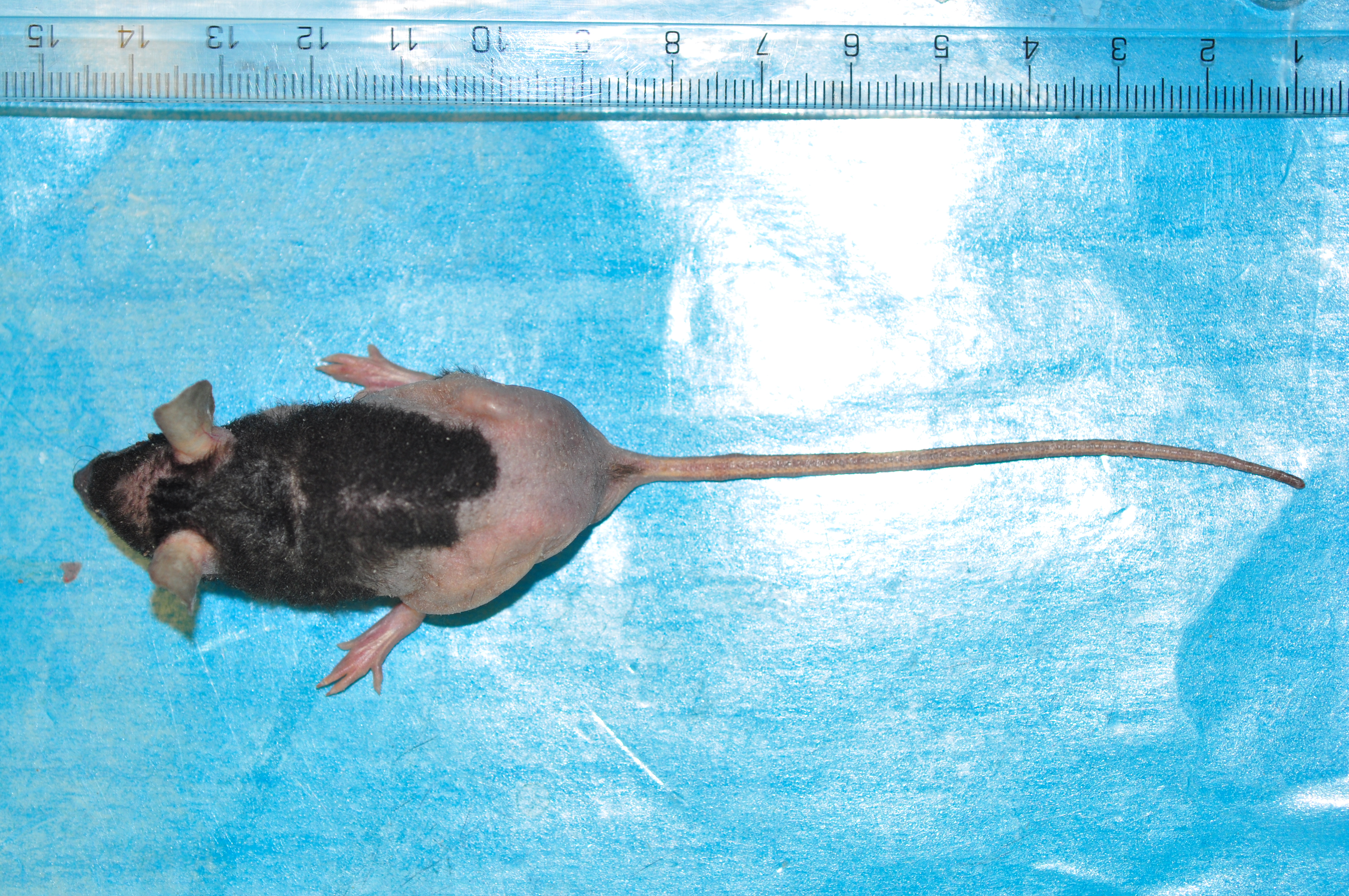

Supplement: Supplementary file 5 — Source data Fig. 5 [file 44321_2024_166_MOESM5_ESM.zip › EMM-2024-20141-V3_Source data for Figure 5/Figure 5K Image data/Fig 5K EGFRSTAT1Dep.JPG]

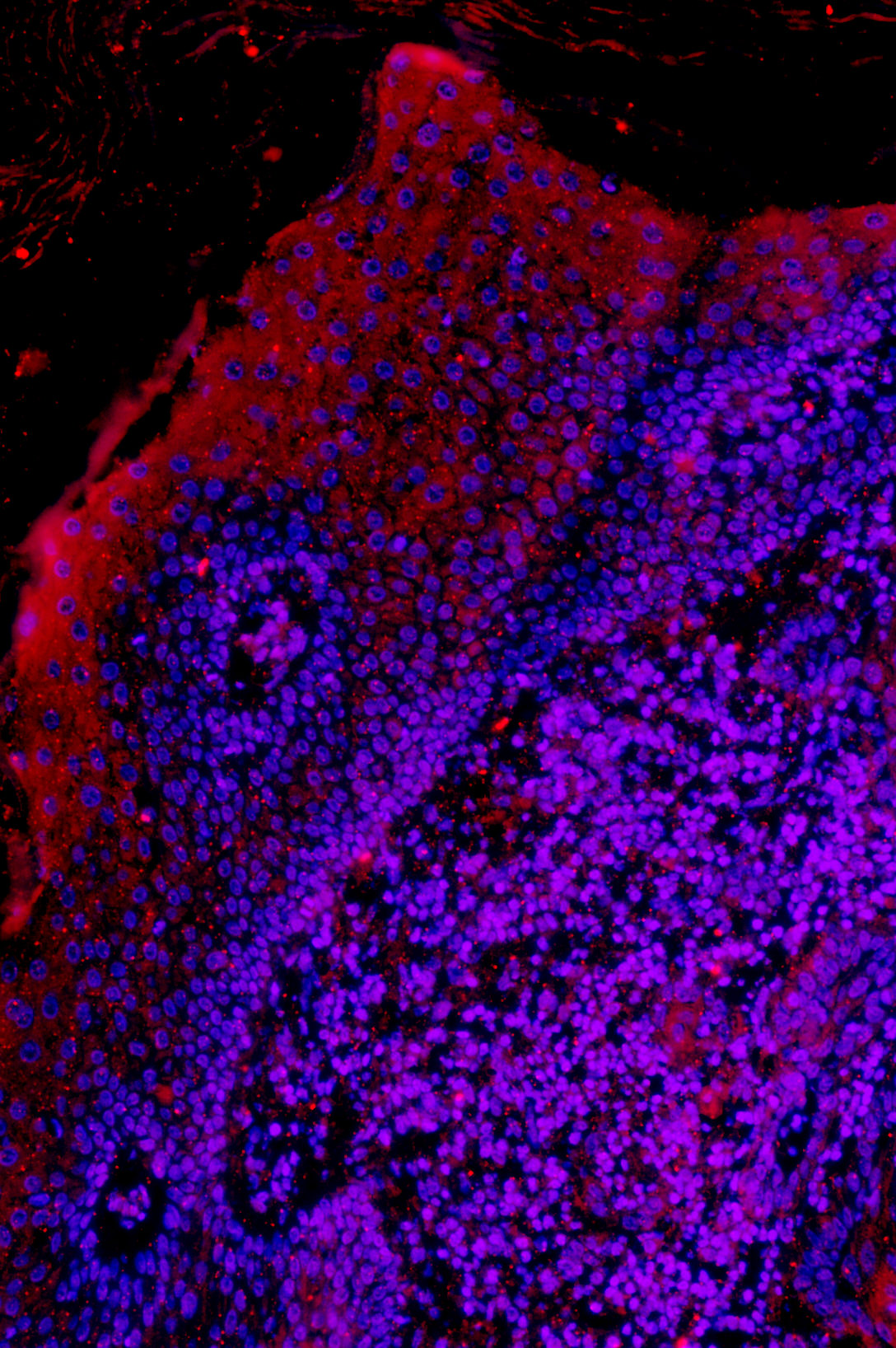

Supplement: Supplementary file 6 — Source data Fig. 6 [file 44321_2024_166_MOESM6_ESM.zip › EMM-2024-20141-V3_Source data for Figure 6/Figure 6A Image data/SCC I Cetuximab.tif]

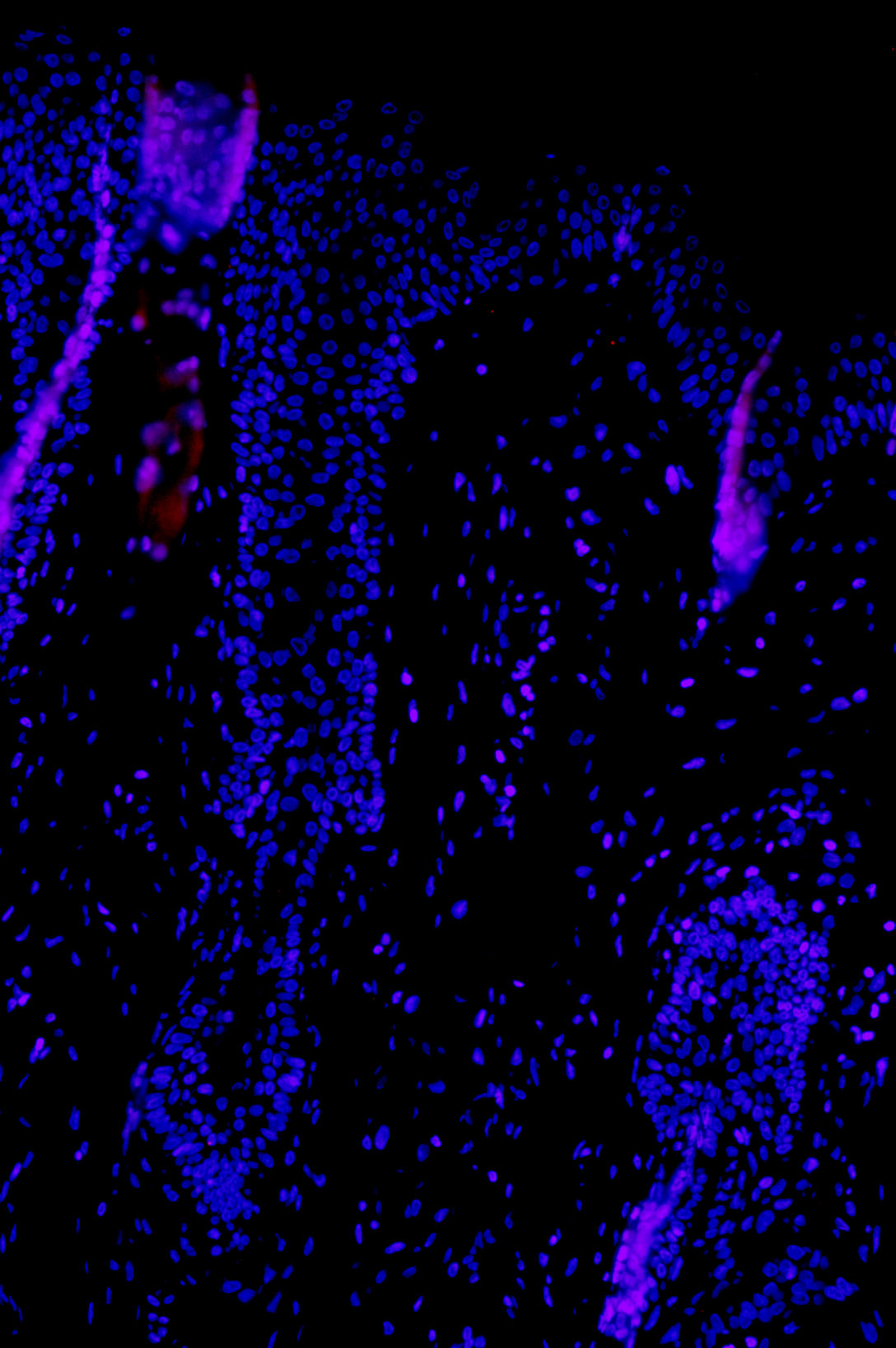

Supplement: Supplementary file 6 — Source data Fig. 6 [file 44321_2024_166_MOESM6_ESM.zip › EMM-2024-20141-V3_Source data for Figure 6/Figure 6A Image data/SCC I Pre-Treatment.tif]

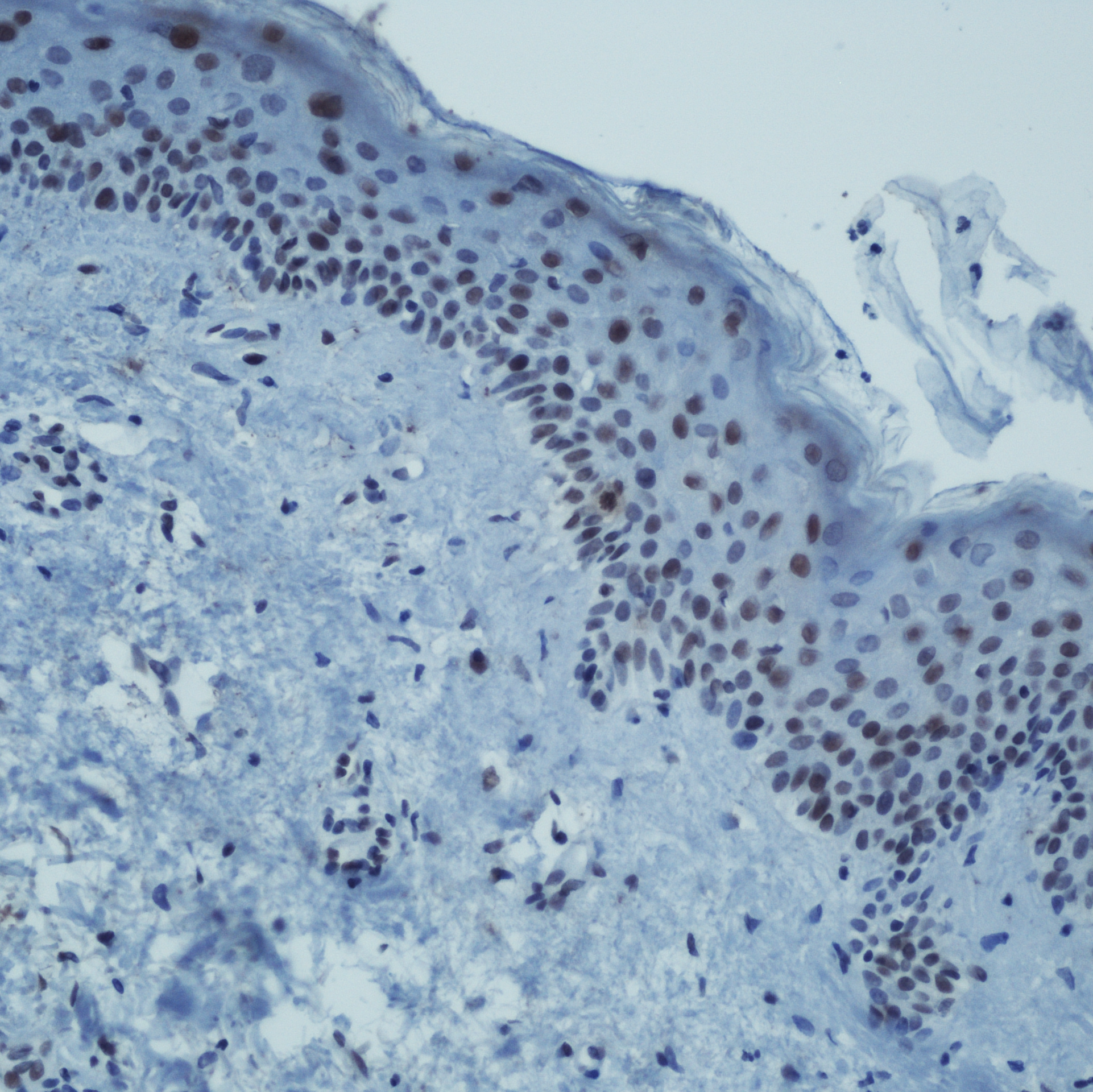

Supplement: Supplementary file 6 — Source data Fig. 6 [file 44321_2024_166_MOESM6_ESM.zip › EMM-2024-20141-V3_Source data for Figure 6/Figure 6B Image data/Cetuxi II 20x.tif]

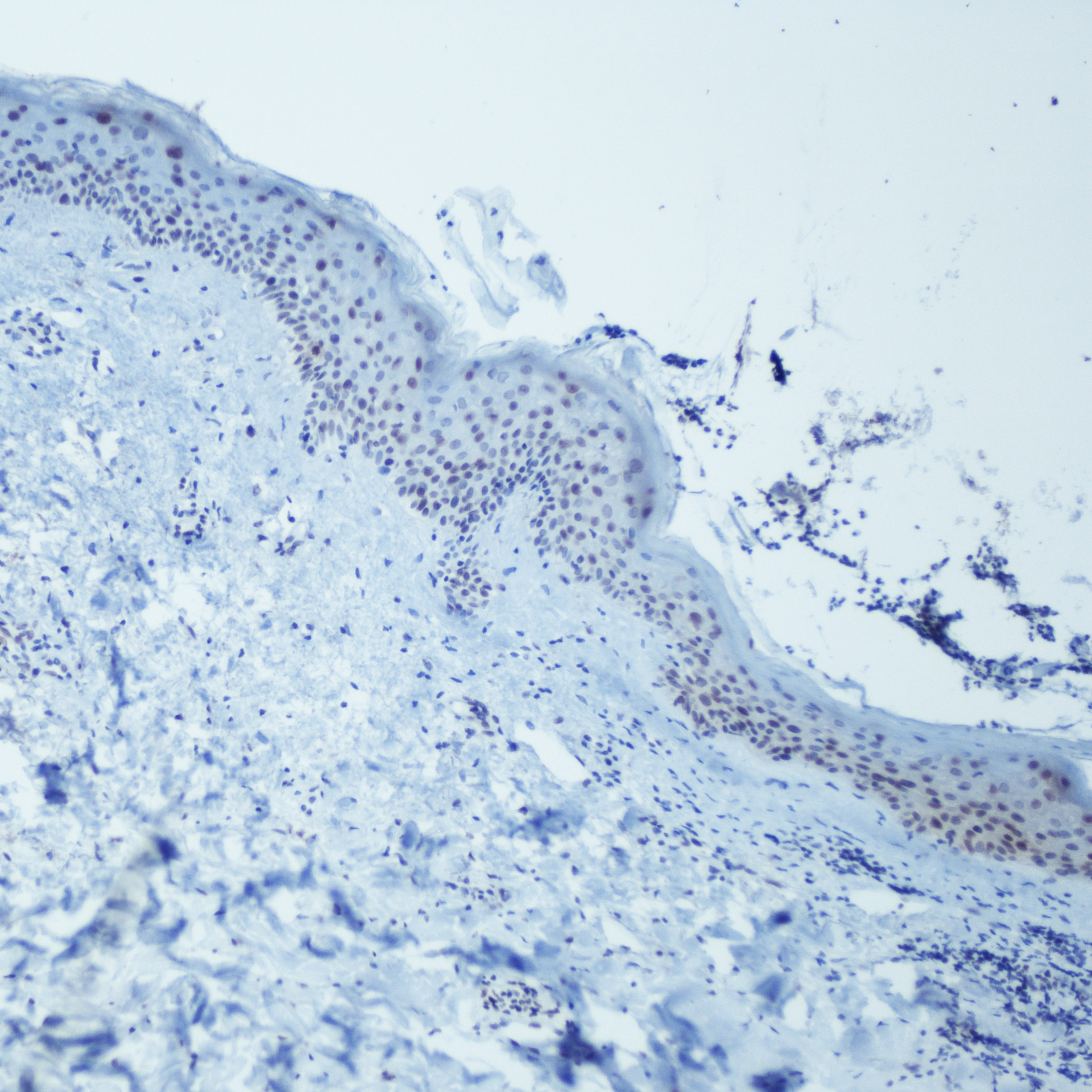

Supplement: Supplementary file 6 — Source data Fig. 6 [file 44321_2024_166_MOESM6_ESM.zip › EMM-2024-20141-V3_Source data for Figure 6/Figure 6B Image data/Cetuxi II.tif]

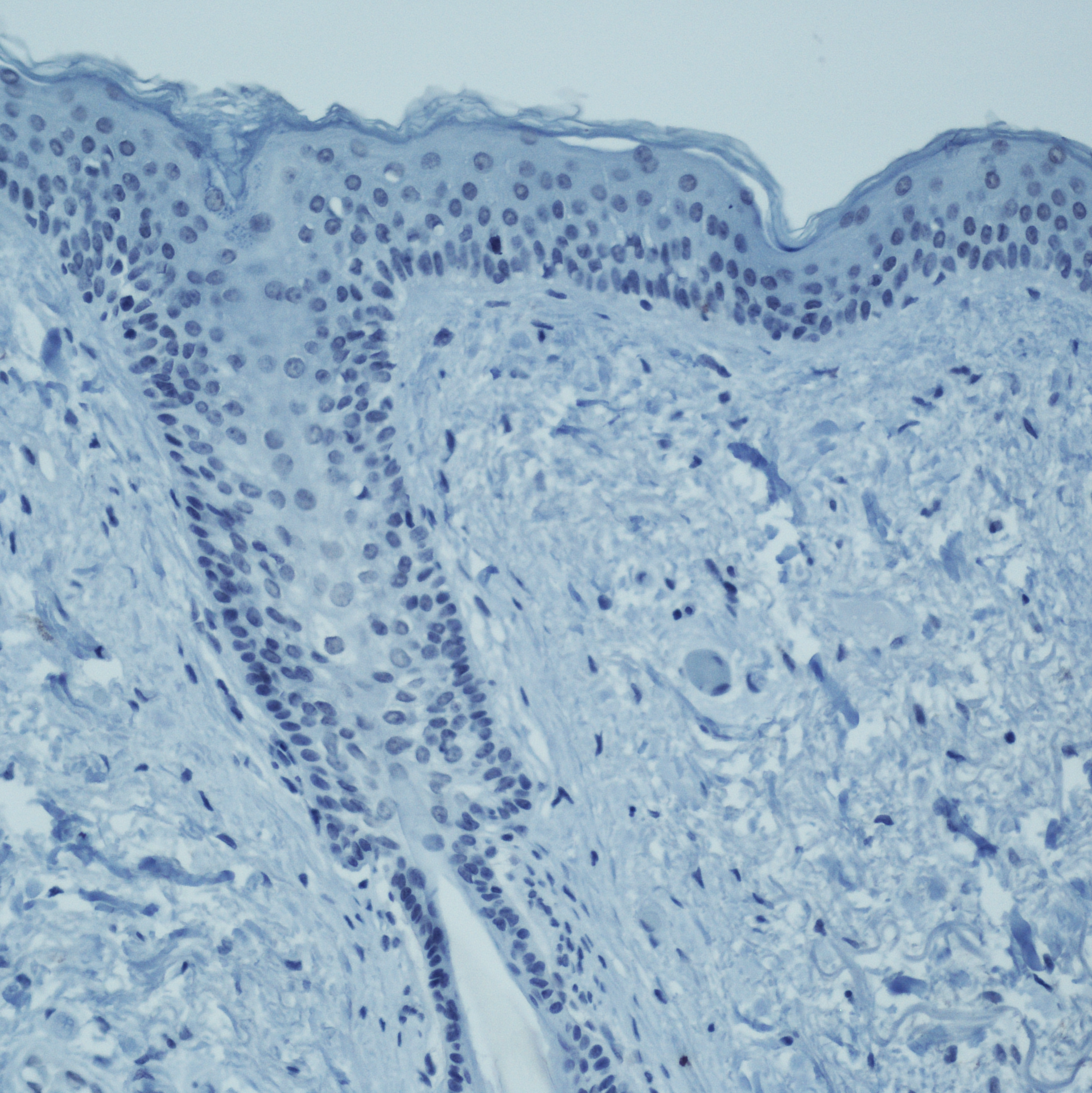

Supplement: Supplementary file 6 — Source data Fig. 6 [file 44321_2024_166_MOESM6_ESM.zip › EMM-2024-20141-V3_Source data for Figure 6/Figure 6B Image data/pre treat I 20x.tif]

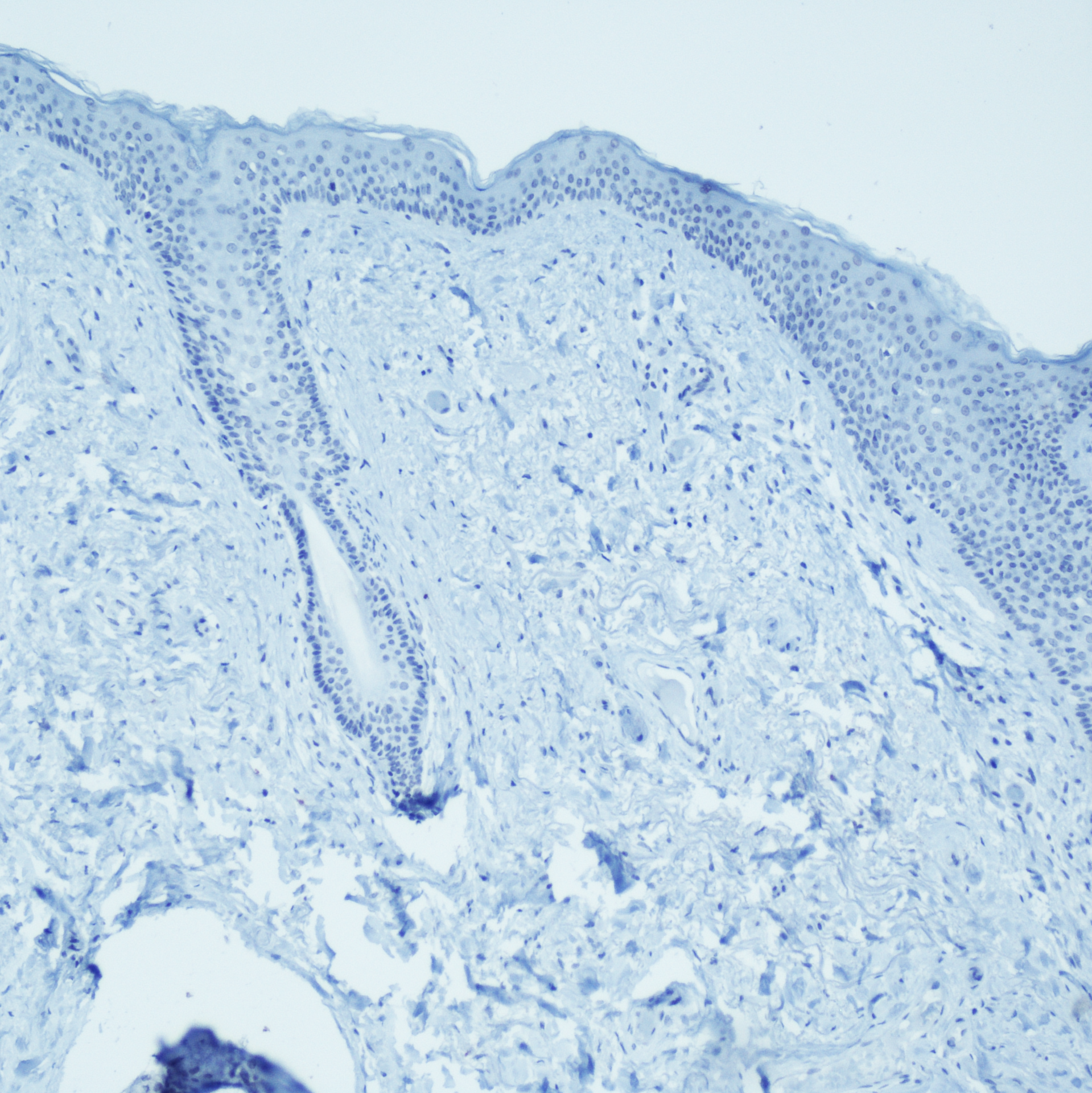

Supplement: Supplementary file 6 — Source data Fig. 6 [file 44321_2024_166_MOESM6_ESM.zip › EMM-2024-20141-V3_Source data for Figure 6/Figure 6B Image data/pre treat I.tif]

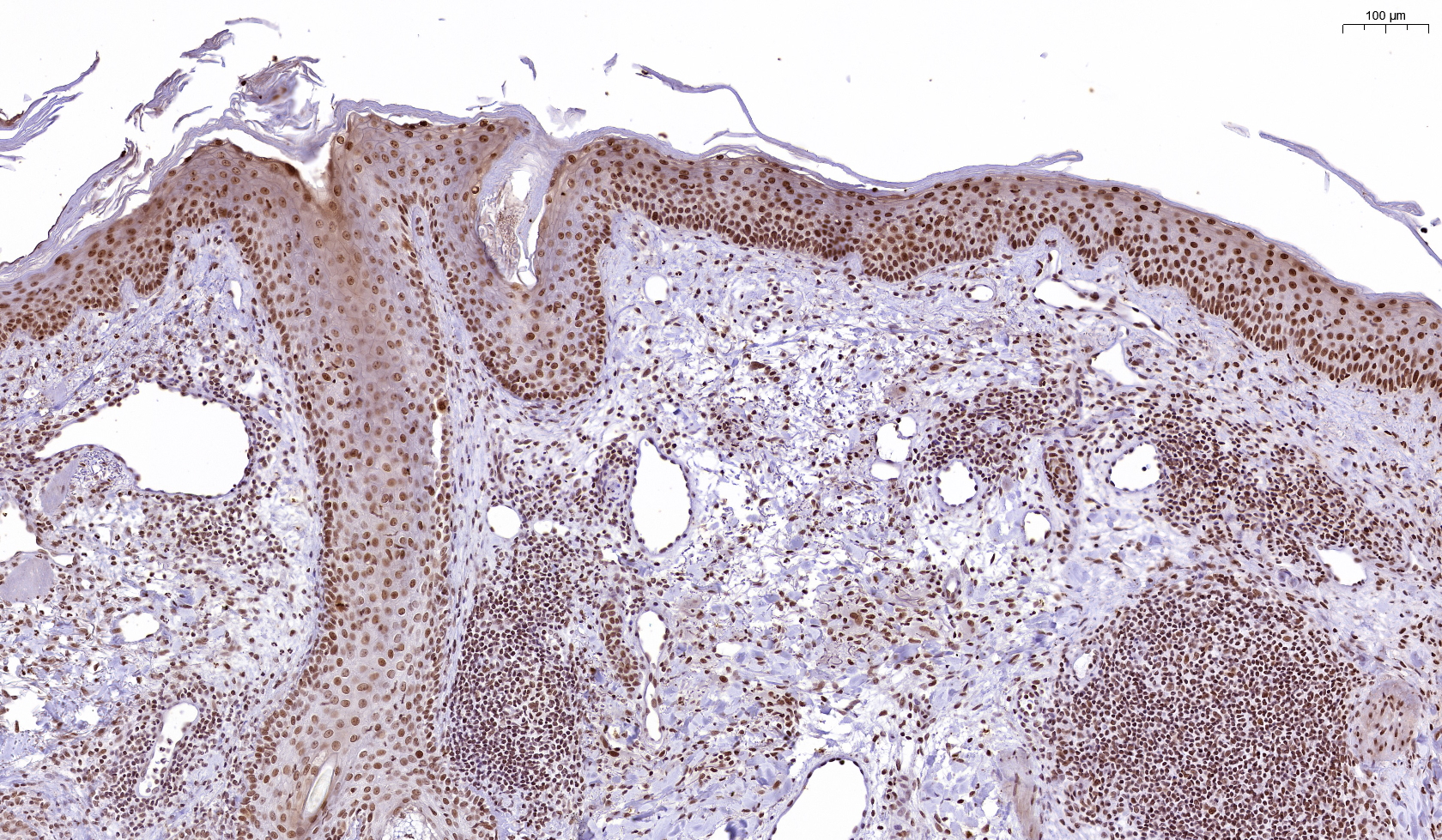

Supplement: Supplementary file 6 — Source data Fig. 6 [file 44321_2024_166_MOESM6_ESM.zip › EMM-2024-20141-V3_Source data for Figure 6/Figure 6D Image data/pSTAT1 FD4_10.0x.jpg]

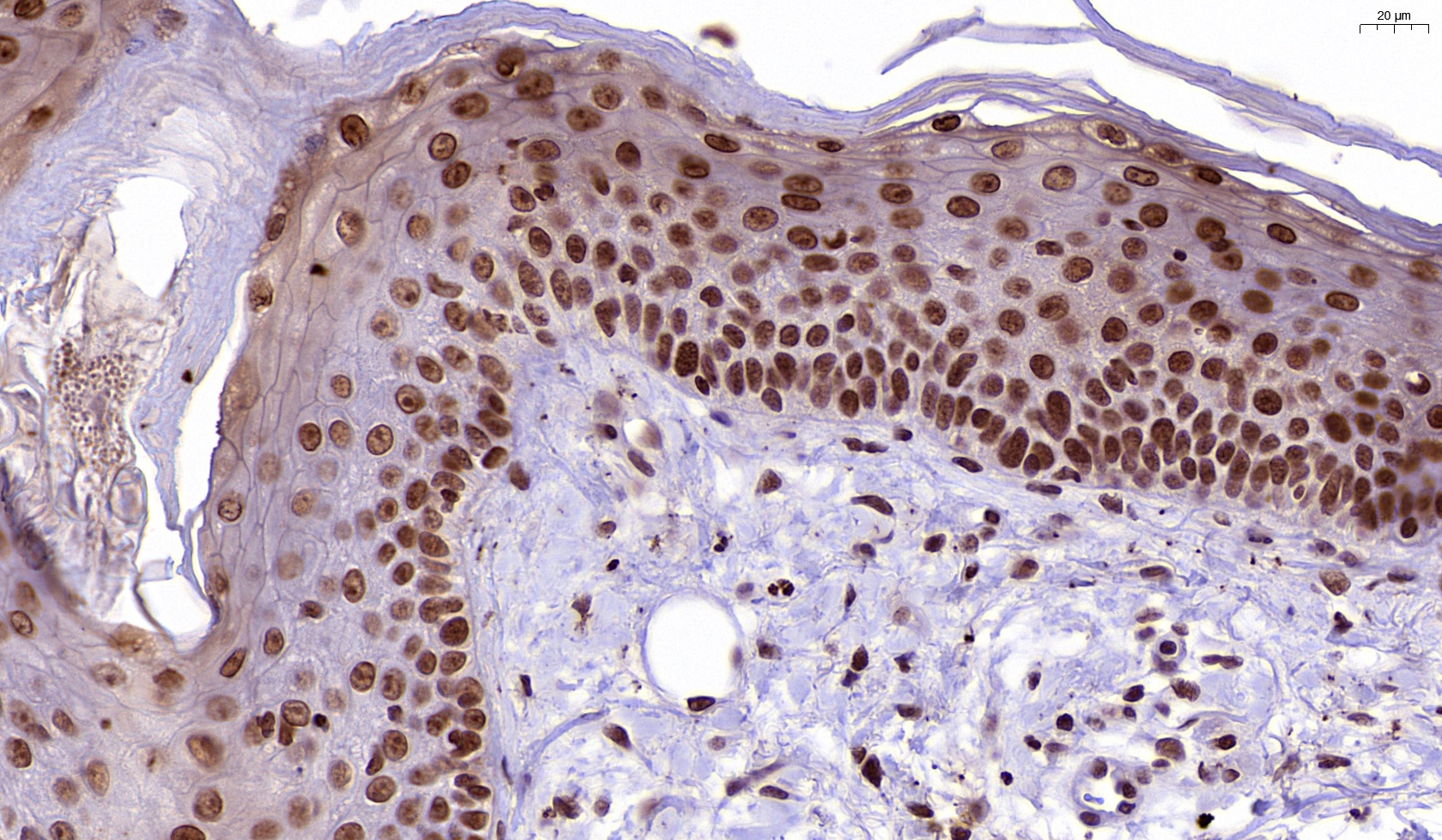

Supplement: Supplementary file 6 — Source data Fig. 6 [file 44321_2024_166_MOESM6_ESM.zip › EMM-2024-20141-V3_Source data for Figure 6/Figure 6D Image data/pSTAT1 FD4_40.0x.jpg]

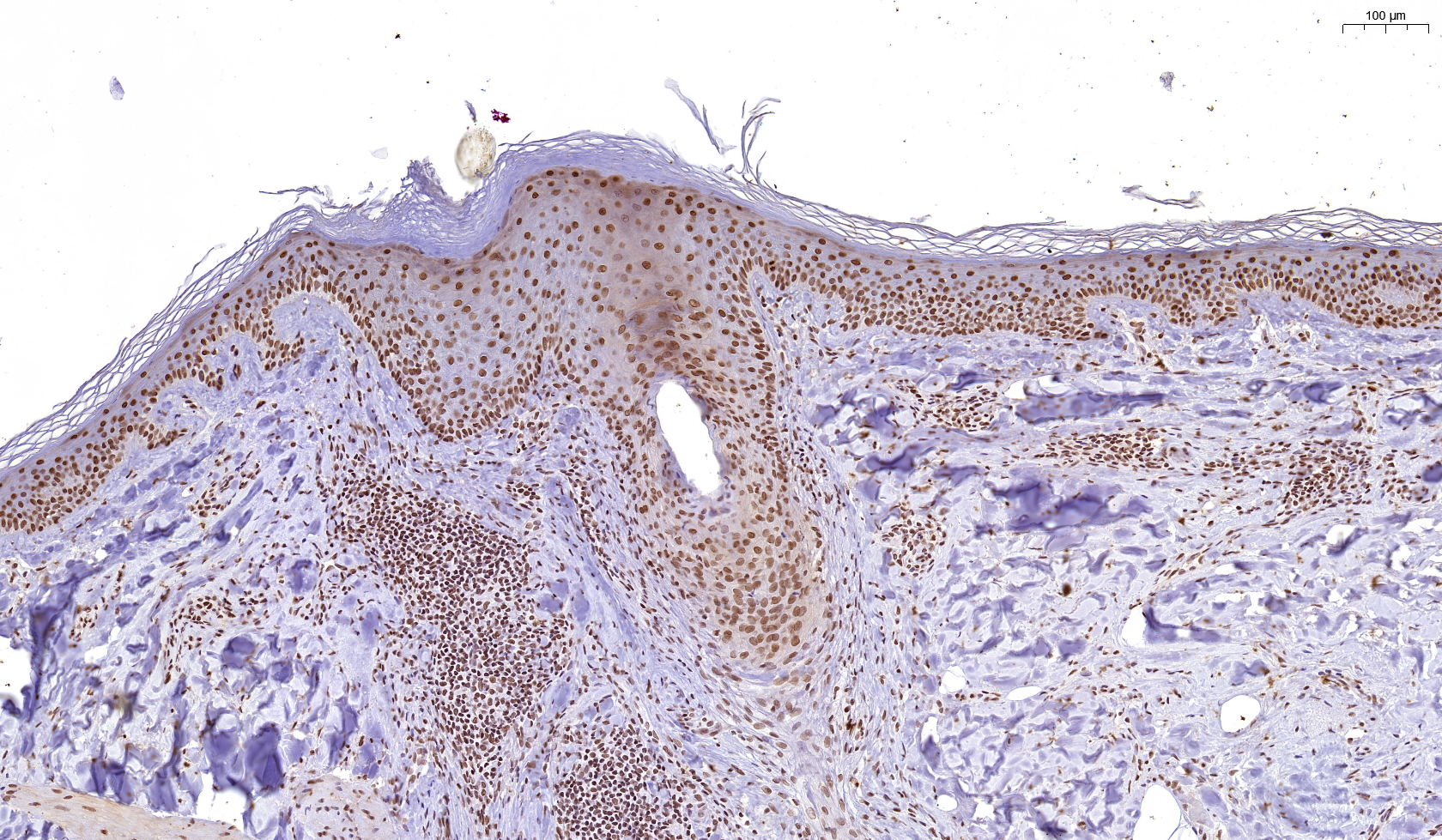

Supplement: Supplementary file 6 — Source data Fig. 6 [file 44321_2024_166_MOESM6_ESM.zip › EMM-2024-20141-V3_Source data for Figure 6/Figure 6D Image data/pSTAT1 FFA4_10.0x.jpg]

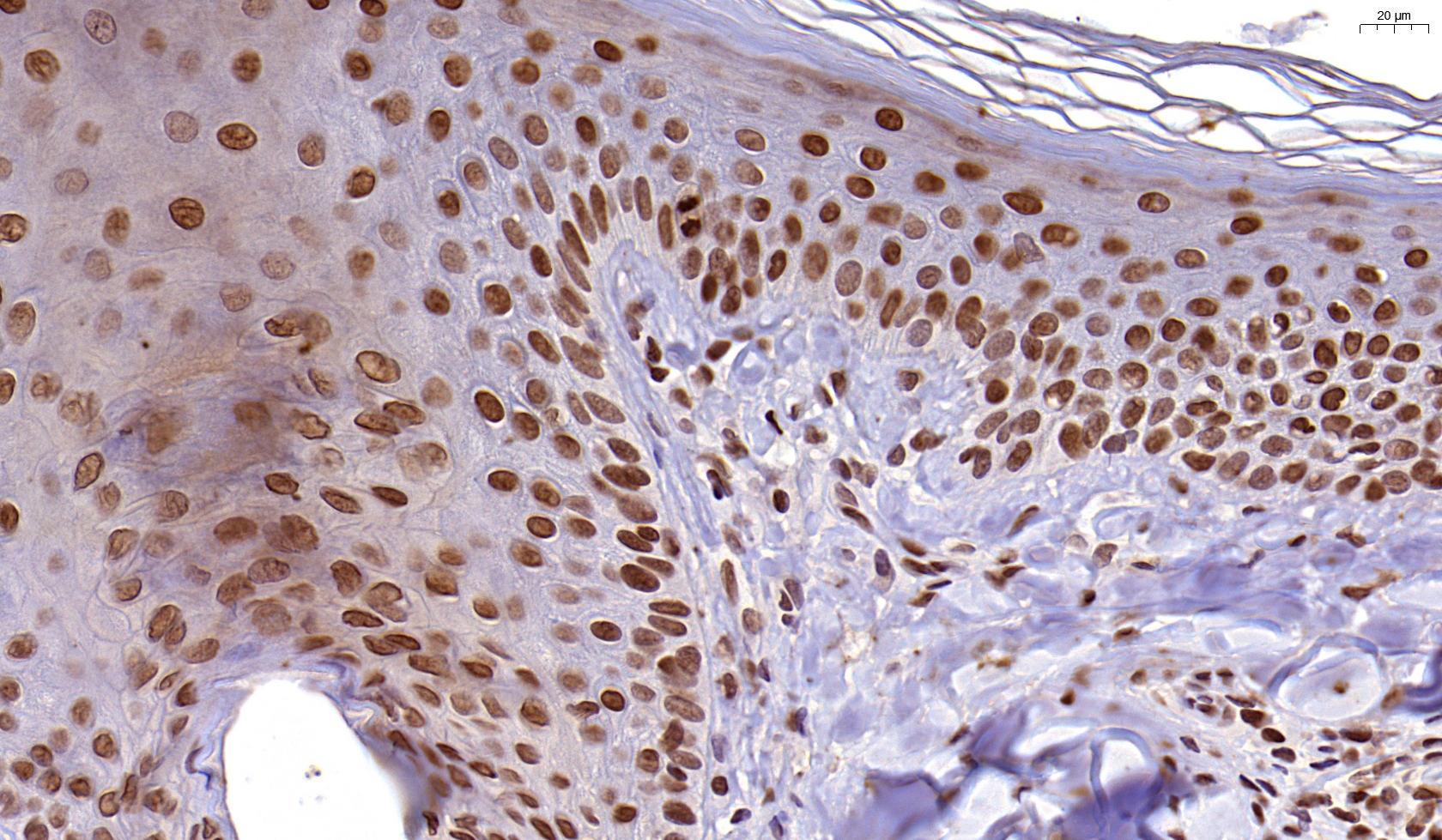

Supplement: Supplementary file 6 — Source data Fig. 6 [file 44321_2024_166_MOESM6_ESM.zip › EMM-2024-20141-V3_Source data for Figure 6/Figure 6D Image data/pSTAT1 FFA4_40.0x.jpg]

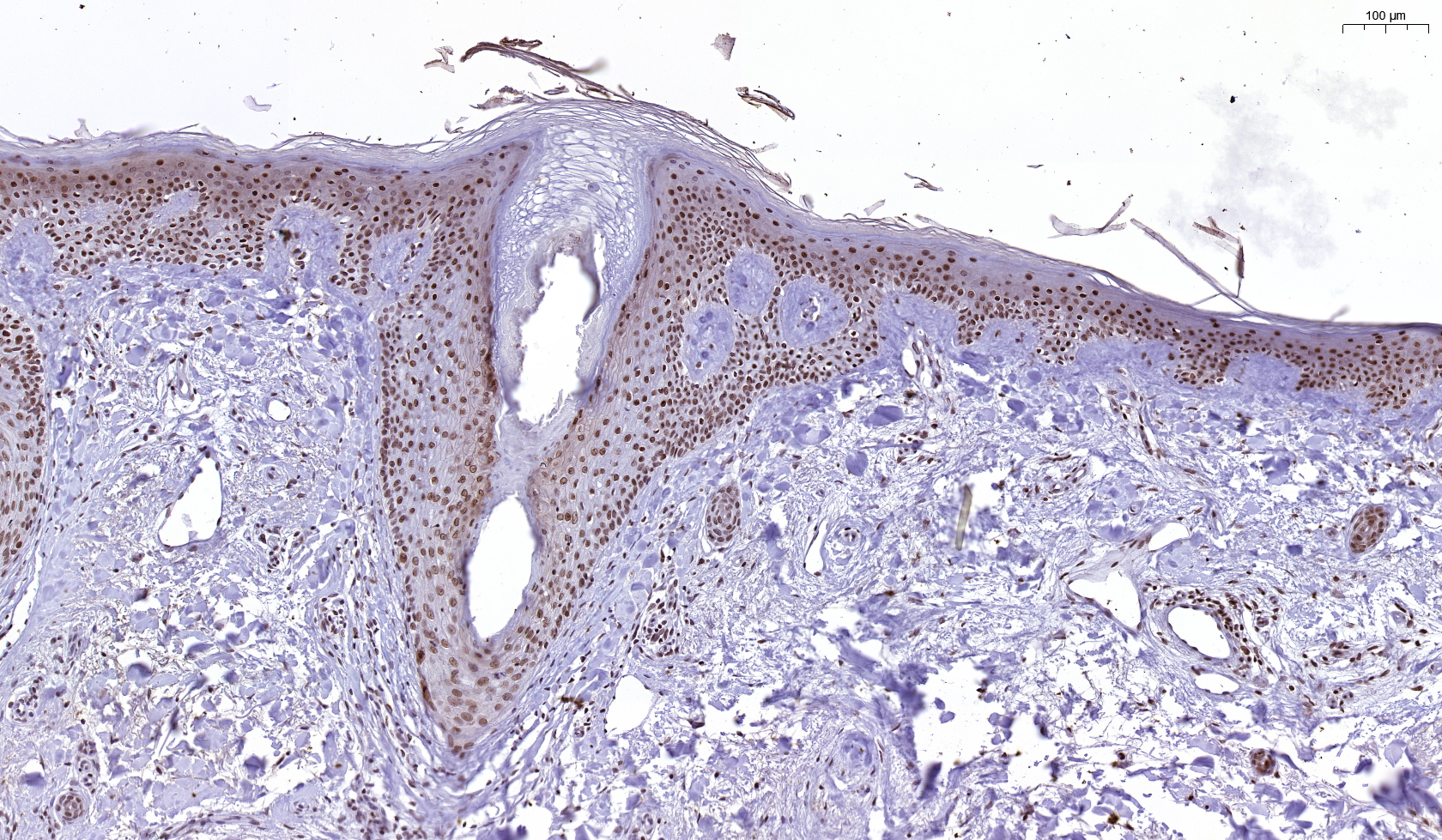

Supplement: Supplementary file 6 — Source data Fig. 6 [file 44321_2024_166_MOESM6_ESM.zip › EMM-2024-20141-V3_Source data for Figure 6/Figure 6D Image data/pSTAT1 LPP9_10.0x.jpg]

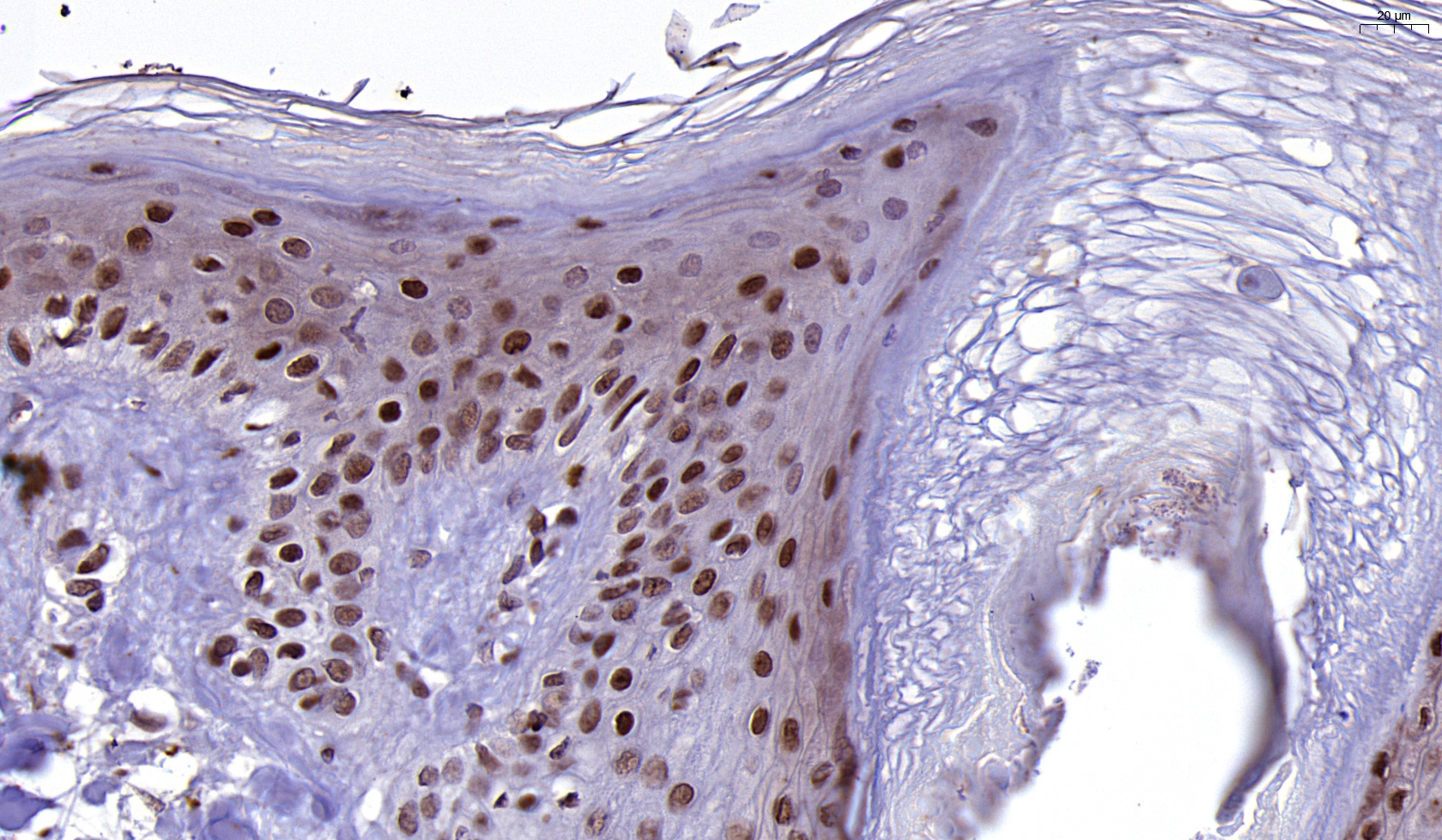

Supplement: Supplementary file 6 — Source data Fig. 6 [file 44321_2024_166_MOESM6_ESM.zip › EMM-2024-20141-V3_Source data for Figure 6/Figure 6D Image data/pSTAT1 LPP9_40.0x.jpg]

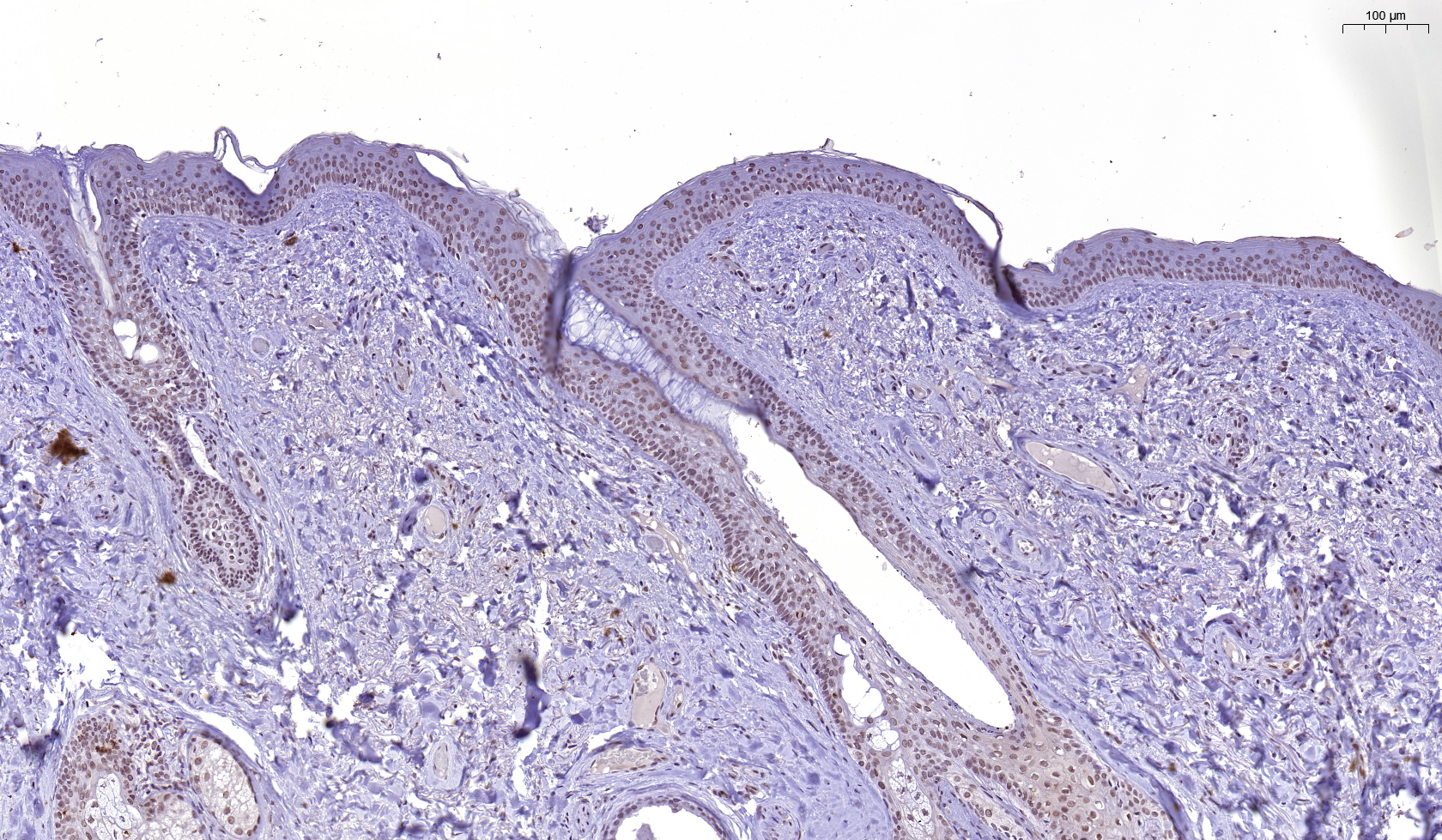

Supplement: Supplementary file 6 — Source data Fig. 6 [file 44321_2024_166_MOESM6_ESM.zip › EMM-2024-20141-V3_Source data for Figure 6/Figure 6D Image data/pSTAT1 Pretreat 1807_10.0x.jpg]

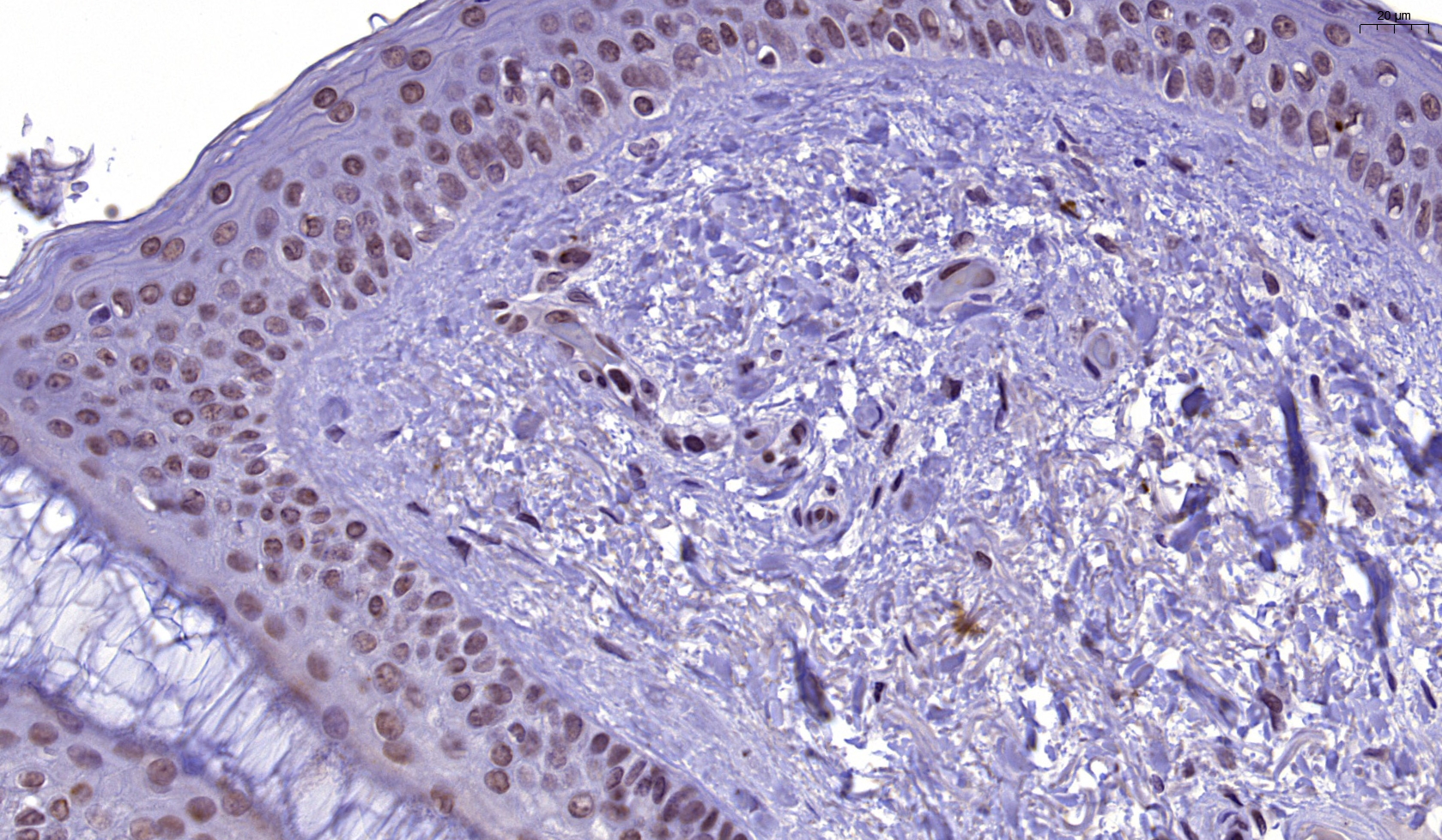

Supplement: Supplementary file 6 — Source data Fig. 6 [file 44321_2024_166_MOESM6_ESM.zip › EMM-2024-20141-V3_Source data for Figure 6/Figure 6D Image data/pSTAT1 Pretreat 1807_40.0x.jpg]
