# Supplementary material for: JAK-STAT1 as therapeutic target for EGFR deficiency-associated inflammation and scarring alopecia
Source: EMBO Mol Med. 2024 Nov 9;16(12):7. doi: 10.1038/s44321-024-00166-3 (PMC11628629; doi:10.1038/s44321-024-00166-3)
Supplement: Supplementary file 7 — Expanded View Figures [file 44321_2024_166_MOESM7_ESM.pdf]

Expanded View Figures

Figure EV1. Hair follicle-specific EGFR deletion induces epidermal immune infiltrate and microbiota-driven inflammatory hair follicle destruction.

(**A**) Representative pictures of the gating strategy of FACS analysis of epidermal single cell suspensions (WT and $EGFR^{\Delta Egr2}$ mice). $CD34^{+}$ $Sca\text{-}I^{-}$ HFSC, $CD34^{-}$ $Sca\text{-}I^{-}$ HF and $Sca\text{-}I^{+}$ IFE among $CD45^{-}$ keratinocytes. $\gamma\delta TCR^{hi}$ dendritic epidermal T cells (DETC), $\gamma\delta TCR^{int}$ γδT cells (γδTC), αβT cells (αβTC), $CD11b^{+}Gr\text{-}1^{+}$ neutrophils, $NK1.1^{+}$ $Nkp46^{+}$ natural killer (NK) cells, $CD11b^{+}Epcam^{+}$ Langerhans cells (LC) among $CD45^{+}$ immune cells. (**B**) FACS analysis of αβT cells (WT vs $EGFR^{\Delta ep}$ $p = 0.0067$, $EGFR^{\Delta ep}$ vs WT Abx $p = 0.001$, $EGFR^{\Delta ep}$ vs $EGFR^{\Delta ep}$ K5-SOS Abx $p = 0.0057$) and γδT cells (WT vs $EGFR^{\Delta ep}$ K5-SOSp <0.0001, $EGFR^{\Delta ep}$ vs $EGFR^{\Delta ep}$ K5-SOS $p = 0.013$, $EGFR^{\Delta ep}$ K5-SOS vs WT Abx $p < 0.0001$, $EGFR^{\Delta ep}$ K5-SOS vs $EGFR^{\Delta ep}$ Abx $p = 0.0026$, $EGFR^{\Delta ep}$ K5-SOS vs $EGFR^{\Delta ep}$ K5-SOS Abx $p = 0.0004$) among $CD45^{+}$ immune cells at 2 M. Each dot represents an independent mouse. Mouse models as indicated in the graph. (**C**) Kaplan–Meier survival plot of $EGFR^{\Delta Egr2}$ mice or WT. (**D**) Quantification of EGFR immunohistochemistry staining by Definiens software. Percentage of EGFR expression in hair follicle ($p = 0.0165$) and epidermis of $EGFR^{\Delta Egr2}$ mice or WT at 3 M. (**E**) Representative pictures of hair follicle length measured from hematoxylin and eosin (H&E) stained skin sections marked in yellow dotted lines of WT or $EGFR^{\Delta Egr2}$. (**F**) Sox9 (red) and CD34 (green) positive stem cells of WT and $EGFR^{\Delta Egr2}$ mice at 2, 3, and 5 months. Quantified by Halo AI software in percentage of the hair follicle ($CD34^{+}$ $p = 0.0227$, $Sox9^{+}$ $p = 0.0055$). (**G**) Timeline P8-P150 showing representative pictures of H&E stainings of WT and $EGFR^{\Delta Egr2}$. Data is presented in ±SEM, *$p < 0.05$, **$p < 0.01$, ***$p < 0.001$, ****$p < 0.0001$ by One-Way ANOVA with Tukey's posthoc correction, $n \geq 3$.

 © The Author(s)

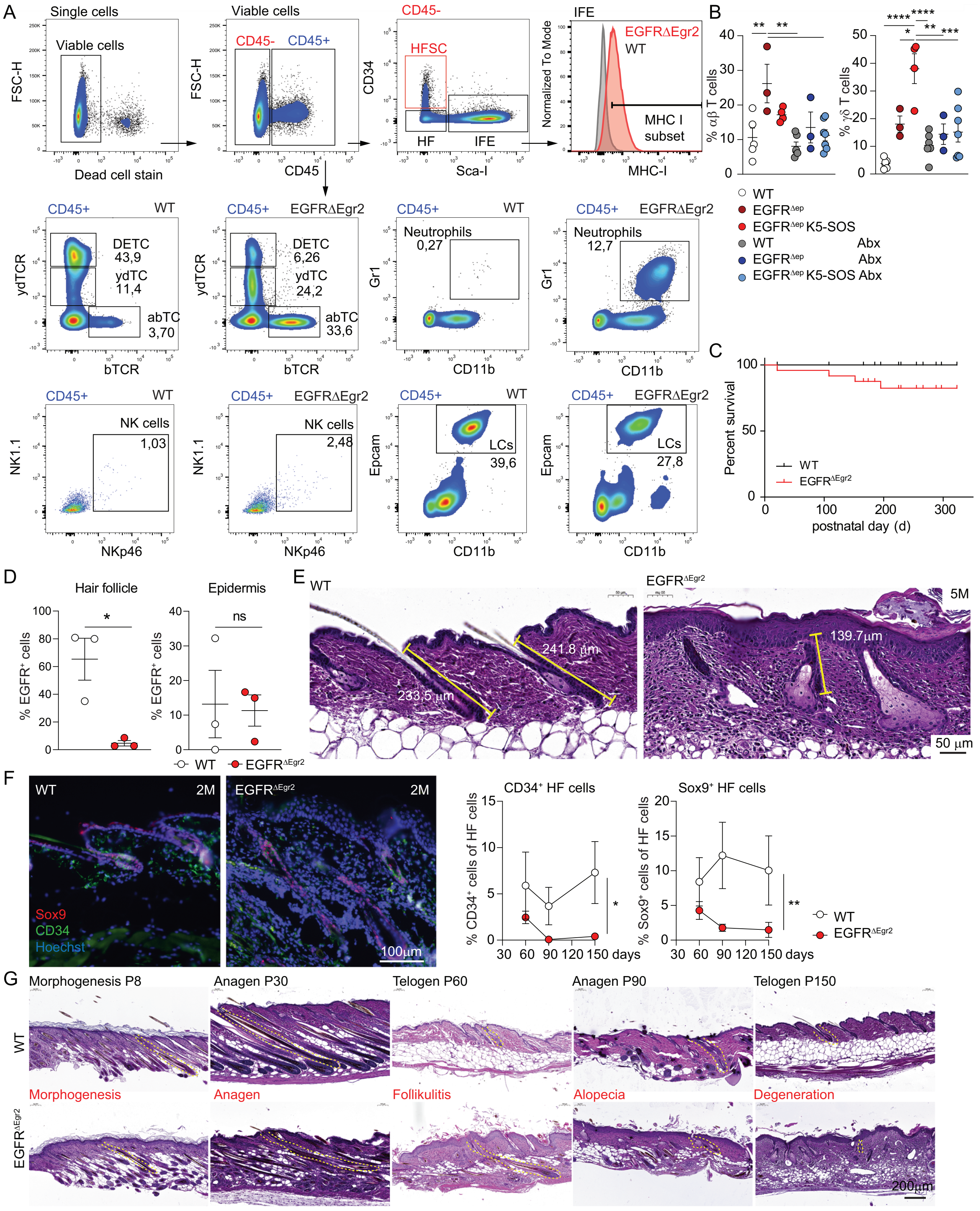
A
Single cells
Viable cells
Dead cell stain
FSC-H
Viable cells
CD45-
CD45+
CD45
CD45-
HFSC
HF
IFE
CD34
Sca-I
IFE
EGFRΔEgr2
WT
MHC I subset
Normalized To Mode
MHC-I
CD45+ WT
DETC 43,9
ydTC 11,4
abTC 3,70
ydTCR
bTCR
CD45+ EGFRΔEgr2
DETC 6,26
ydTC 24,2
abTC 33,6
CD45+ WT
Neutrophils 0,27
Gr1
CD11b
CD45+ EGFRΔEgr2
Neutrophils 12,7
CD45+ WT
NK cells 1,03
NK1.1
NKp46
CD45+ EGFRΔEgr2
NK cells 2,48
CD45+ WT
LCs 39,6
Epcam
CD11b
CD45+ EGFRΔEgr2
LCs 27,8
B
% αβ T cells
% γδ T cells
WT
EGFR^Δep
EGFR^Δep K5-SOS
WT Abx
EGFR^Δep Abx
EGFR^Δep K5-SOS Abx
C
Percent survival
WT
EGFR^ΔEgr2
postnatal day (d)
D
Hair follicle
Epidermis
% EGFR+ cells
ns
WT
EGFR^ΔEgr2
E
WT
EGFR^ΔEgr2
5M
233,5 µm
241,8 µm
139.7µm
50 µm
F
WT 2M
EGFR^ΔEgr2 2M
Sox9
CD34
Hoechst
100µm
CD34+ HF cells
Sox9+ HF cells
% CD34+ cells of HF cells
% Sox9+ cells of HF cells
days
WT
EGFR^ΔEgr2
G
Morphogenesis P8
Anagen P30
Telogen P60
Anagen P90
Telogen P150
WT
EGFR^ΔEgr2
Morphogenesis
Anagen
Follikulitis
Alopecia
Degeneration
200µm

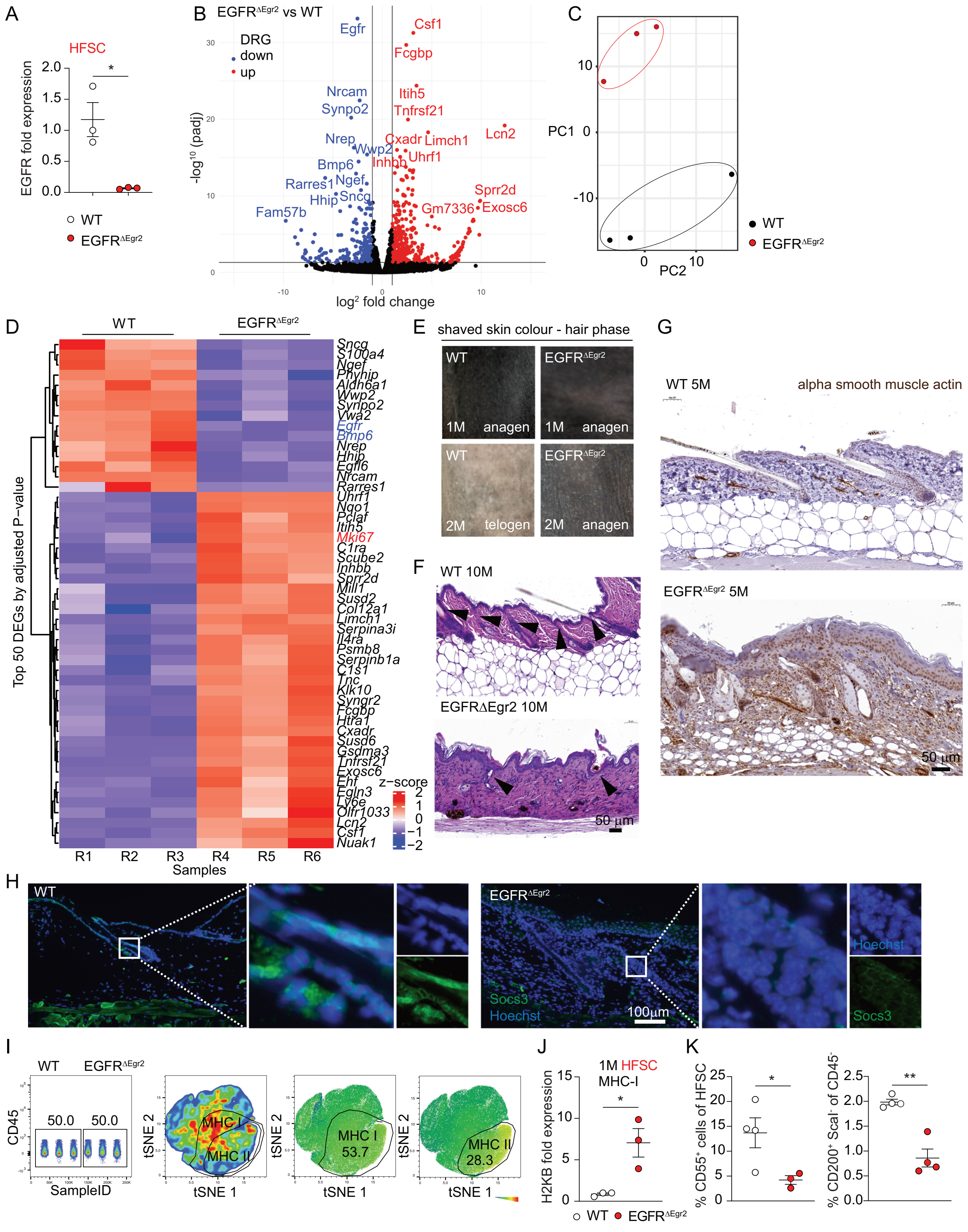
A
HFSC
EGFR fold expression
WT
EGFRΔEgr2
B EGFRΔEgr2 vs WT
DRG
down
up
-log10 (padj)
log2 fold change
Egfr
Csf1
Fcgbp
Nrcam
Synpo2
Itih5
Tnfrsf21
Lcn2
Nrep
Wwp2
Cxadr
Limch1
Inhbb
Uhrf1
Bmp6
Rarres1
Ngef
Hhip
Sncg
Sprr2d
Exosc6
Gm7336
Fam57b
C
PC1
PC2
WT
EGFRΔEgr2
D
WT
EGFRΔEgr2
Top 50 DEGs by adjusted P−value
Samples
R1 R2 R3 R4 R5 R6
z−score
E shaved skin colour - hair phase
WT 1M anagen
EGFRΔEgr2 1M anagen
WT 2M telogen
EGFRΔEgr2 2M anagen
F WT 10M
EGFRΔEgr2 10M
50 µm
G
WT 5M
alpha smooth muscle actin
EGFRΔEgr2 5M
50 µm
H
WT
EGFRΔEgr2
Socs3
Hoechst
100µm
I
WT EGFRΔEgr2
CD45
SampleID
tSNE 1
tSNE 2
MHC I
MHC II
J
1M HFSC
MHC-I
H2KB fold expression
WT EGFRΔEgr2
K
% CD55+ cells of HFSC
% CD200+ Scal- of CD45-

Figure EV2. RNA profiling of FACS sorted EGFR-deficient hair follicle stem cells before scarring hair follicle destruction.

(**A**) Real-time PCR of EGFR expression of sorted $CD34^{+}ScaI^{-}$ HFSCs of WT and $EGFR^{\Delta Egr2}$ mice at 1 M. Data is presented in ±SEM, $p = 0.0156$ by unpaired t-test, $n \geq 3$. (**B**) Volcano plot of differentially expressed genes (blue downregulated, red upregulated) of RNA sequencing analysis of $CD34^{+}$ HFSCs from WT vs $EGFR^{\Delta Egr2}$ mice. Data shown as fold change (log2) and *p*-value (−log10). DESeq2 including local dispersion estimation and Independent Hypothesis Weighting (IHW) (x) to estimate false discovery rates and power maximization were used for statistical analysis (Ignatiadis et al, 2016). (**C**) Principal component (PC) analysis of the RNAseq dataset. (**D**) Heatmap of z-scores of top 50 differentially expressed genes by adjusted *p*-value of the RNAseq dataset. Genes of special interest are marked in red (up) or blue (down). Statistics DESeq2 as in (**B**). (**E**) Pictures of shaved backs of WT or $EGFR^{\Delta Egr2}$ at 1 M and 2 M of age. (**F**) Representative H&E stained skin sections for counting the number of hair follicle units per 1500 µm of WT and $EGFR^{\Delta Egr2}$ skin at 10 M of age. Black arrowheads indicate counted hair follicles. (**G**) Representative alpha smooth muscle actin staining on skin sections of WT and $EGFR^{\Delta Egr2}$ skin at 5 M of age. (**H**) Immunofluorescence staining of SOCS3 in green of WT and $EGFR^{\Delta Egr2}$ mouse skin sections at 3 M. (**I**) tSNE FACS gating strategy of MHC-I and MHC-II expression among $CD45^{-}$ keratinocytes of WT or $EGFR^{\Delta Egr2}$ at 5 M of age. (**J**) Real-time PCR of MHC-I expression (H-2Kb) of sorted $CD34^{+}$ $ScaI^{-}$ HFSCs of WT or $EGFR^{\Delta Egr2}$ mice at 1 M of age ($p = 0.0229$). (**K**) CD55 ($p = 0.0481$) and CD200 ($p = 0.001$) surface expression in HFSCs and HF of WT and $EGFR^{\Delta Egr2}$ mice by FACS analysis. Data is presented in ±SEM, $^{*}p < 0.05$, $^{**}p < 0.01$ by unpaired t-test, $n \geq 3$.

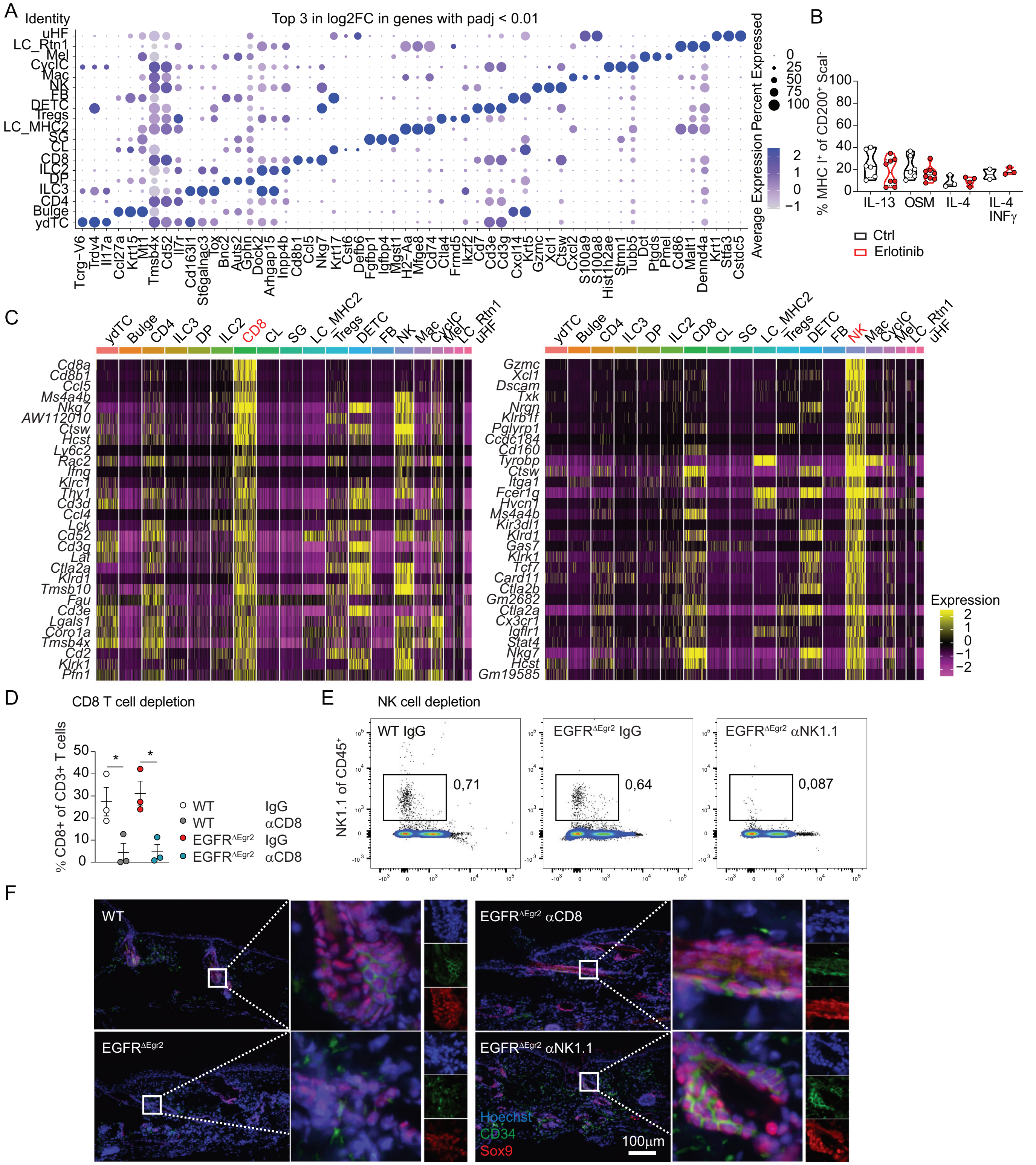
A
Identity
Top 3 in log2FC in genes with padj < 0.01
uHF
LC_Rtn1
Mel
CyclC
Mac
NK
FB
DETC
Tregs
LC_MHC2
SG
CL
CD8
ILC2
DP
ILC3
CD4
Bulge
ydTC
Percent Expressed
0
25
50
75
100
Average Expression
2
1
0
−1
B
% MHC I+ of CD200+ Scal-
IL-13
OSM
IL-4
IL-4 INFγ
Ctrl
Erlotinib
C
Expression
D
CD8 T cell depletion
% CD8+ of CD3+ T cells
WT IgG
WT αCD8
EGFRΔEgr2 IgG
EGFRΔEgr2 αCD8
E
NK cell depletion
NK1.1 of CD45+
WT IgG
0,71
EGFRΔEgr2 IgG
0,64
EGFRΔEgr2 αNK1.1
0,087
F
WT
EGFRΔEgr2
EGFRΔEgr2 αCD8
EGFRΔEgr2 αNK1.1
Hoechst
CD34
Sox9
100μm

Figure EV3. Single-cell analysis identified hair follicle and immune cell populations in EGFR$^{\Delta Egr2}$ mouse epidermal cell suspensions and cytokine impact of HF specific MHC expression.

(**A**) Top 3 differentially expressed genes of every cluster of single-cell RNA sequencing analysis of epidermal CD45^{+} immune cells and CD45^{-} Sca-I^{-} hair follicle cells in EGFR$^{\Delta Egr2}$. Wilcoxon rank sum test and Bonferroni correction for statistical analysis. (**B**) Ex vivo WT skin explants treated with indicated cytokines with or without erlotinib for 48 h. FACS analysis of MHC-I and -II expressions in CD200^{+} HF cells. (**C**) Top 30 differentially expressed genes of CD8 and NK cell cluster. (**D**) Confirmation of CD8 T cell (WT $p = 0.0481$, EGFR$^{\Delta Egr2}$ $p = 0.0246$) and (**E**) NK cell depletion by FACS analysis of EGFR$^{\Delta Egr2}$ mice and their respective controls. (**F**) IF staining of CD34^{+} and Sox9^{+} stem cells in skin sections of EGFR$^{\Delta Egr2}$ treated with the indicated depletion antibodies and the respective controls. Data is presented in ±SEM, *$p < 0.05$ by One-Way ANOVA with Tukey's posthoc correction, $n \geq 3$.

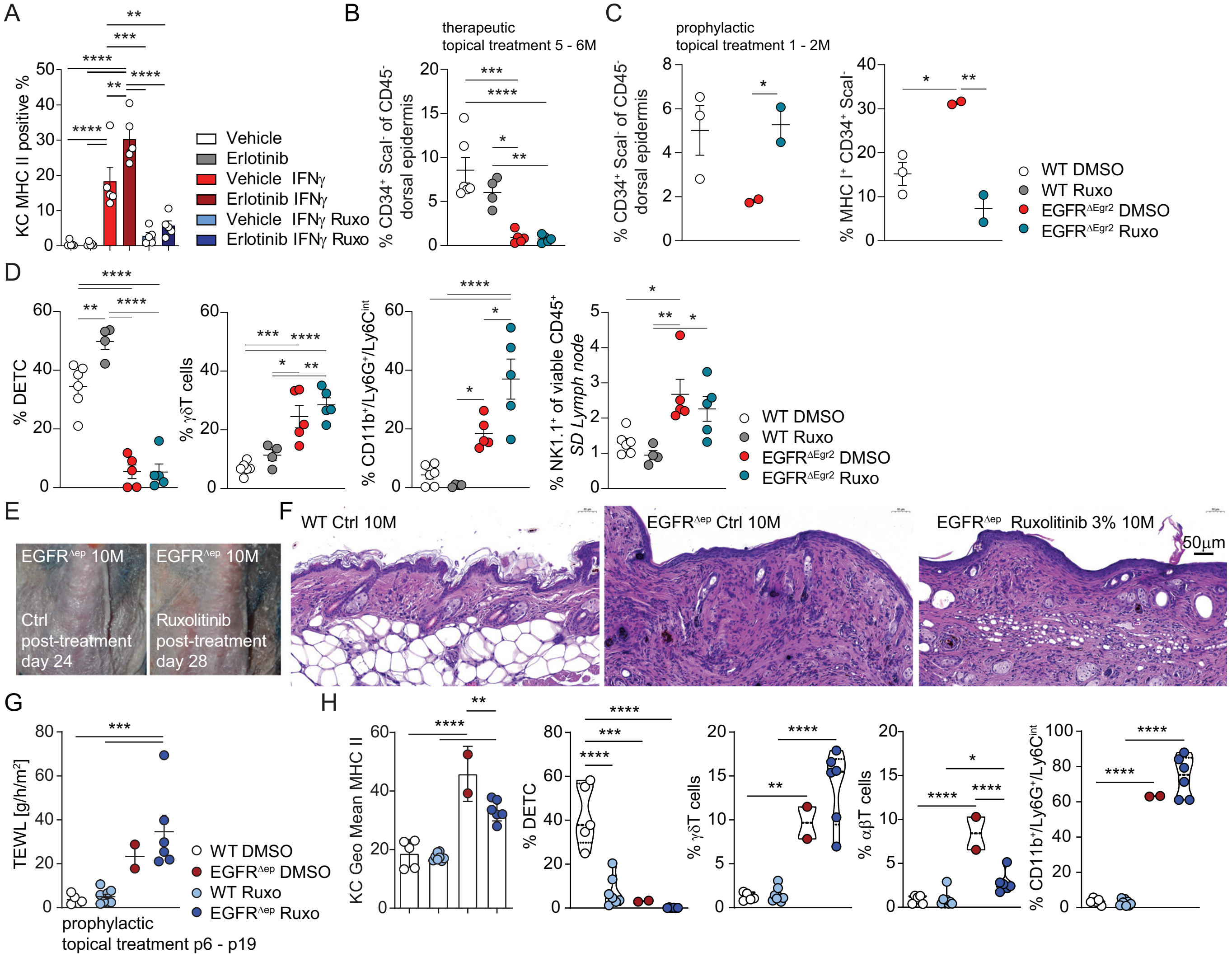

Figure EV4. Therapeutic and prophylactic JAK inhibition ameliorates skin inflammation in EGFR$^{\Delta Egr2}$ and EGFR$^{\Delta ep}$ mice.

(**A**) MHC-II expression of in vitro primary murine KCs of WT mice treated with IFNγ and JAK1/2 inhibitor (ruxolitinib) with or without the EGFR-inhibitor erlotinib (Veh vs Veh IFNγ, Veh vs Erlotinib INFγ, Erlotinib vs Veh IFNγ and Erlotinib vs Erlotinib INFγ $p < 0.0001$, Veh INFγ vs Erlotinib INFγ $p = 0.0085$, Veh INGγ vs Veh INFγ Ruxo $p = 0.0005$, Veh INFγ vs Erlotinib IFNγ Ruxo p = 0.0054, Erlotinib INFγ vs Veh INFγ Ruxo and Erlotinib INFγ vs Erlotinib INFγ Ruxo $p < 0.0001$). (**B**) Summary of FACS analysis of CD34$^+$ hair follicle stem cells from 5 M old WT and EGFR$^{\Delta Egr2}$ mice treated therapeutically with DMSO or ruxolitinib in DMSO for 1 month (WT vs EGFR$^{\Delta Egr2}$ $p = 0.0001$, WT vs EGFR$^{\Delta Egr2}$ Ruxo $p < 0.0001$, EGFR$^{\Delta Egr2}$ vs WT Ruxo $p = 0.0121$, WT Ruxo vs EGFR$^{\Delta Egr2}$ Ruxo $p = 0.0098$). (**C**) FACS analysis of HFSC ($p = 0.0493$) and MHC-I expression (WT vs EGFR$^{\Delta Egr2}$ $p = 0.0224$, EGFR$^{\Delta Egr2}$ vs EGFR$^{\Delta Egr2}$ Ruxo $p = 0.0076$) on EGFR$^{\Delta Egr2}$ and WT mice treated prophylactically with 3% ruxolitinib from 1 to 2 M of age. (**D**) FACS analysis of epidermal CD45$^+$ immune cells of EGFR$^{\Delta Egr2}$ and WT treated therapeutically with 3% Ruxolitinib from 5 M to 6 M (DETC: WT vs WT Ruxo $p = 0.0088$, other $p < 0.0001$, γδT cells: WT vs EGFR$^{\Delta Egr2}$ $p = 0.0004$, WT vs EGFR$^{\Delta Egr2}$ Ruxo $p < 0.0001$, EGFR$^{\Delta Egr2}$ vs WT Ruxo $p = 0.0115$, WT Ruxo vs EGFR$^{\Delta Egr2}$ $p = 0.0012$, CD11b/Ly6G$^+$/Ly6C^{int}: WT vs EGFR$^{\Delta Egr2}$ Ruxo and WT Ruxo vs EGFR$^{\Delta Egr2}$ Ruxo $p < 0.0001$, EGFR$^{\Delta Egr2}$ WT Ruxo $p = 0.0265$, EGFR$^{\Delta Egr2}$ vs EGFR$^{\Delta Egr2}$ Ruxo $p = 0.0131$, NK1.1: WT vs EGFR$^{\Delta Egr2}$ $p = 0.0142$, EGFR$^{\Delta Egr2}$ vs WT Ruxo $p = 0.0056$, WT Ruxo vs EGFR$^{\Delta Egr2}$ Ruxo $p = 0.0381$). (**E, F**) Representative pictures of the skin (**E**) and H&E satinings (**F**) of WT, EGFR$^{\Delta Egr2}$ mice treated topically with DMSO (vehicle ctrl) or 3% ruxolitinib in DMSO daily at the age of 10 M for 4 weeks. (**G, H**) WT and EGFR$^{\Delta ep}$ mice were treated prophylactically with 3% Ruxolitinib or DMSO starting from P6 until P19 and TEWL (WT vs EGFR$^{\Delta Egr2}$ Ruxo $p = 0.0007$, WT Ruxo vs EGFR$^{\Delta Egr2}$ Ruxo $p = 0.0003$) was measured from the back-skin (**G**). FACS analysis of these mice for inflammatory parameters (KC MHC II: EGFR$^{\Delta Egr2}$ vs EGFR$^{\Delta Egr2}$ Ruxo $p = 0.0056$, other p values < 0.0001, DETC: all p values < 0.0001, γδT cells: WT vs EGFR$^{\Delta Egr2}$ $p = 0.0036$, EGFR$^{\Delta Egr2}$ vs WT Ruxo $p = 0.0027$, other $p < 0.0001$, αβT cells: WT vs EGFR$^{\Delta Egr2}$ Ruxo $p = 0.0429$, WT Ruxo vs EGFR$^{\Delta Egr2}$ Ruxo $p = 0.0191$, other $p < 0.0001$, CD11b/Ly6G$^+$/Ly6C^{int}: all p values < 0.0001) as indicated (**H**). Data is presented in ±SEM, $*p < 0.05$, $**p < 0.01$, $***p < 0.00$, $****p < 0.0001$ by one-way ANOVA with Tukey's posthoc correction, $n \geq 3$.

 © The Author(s)

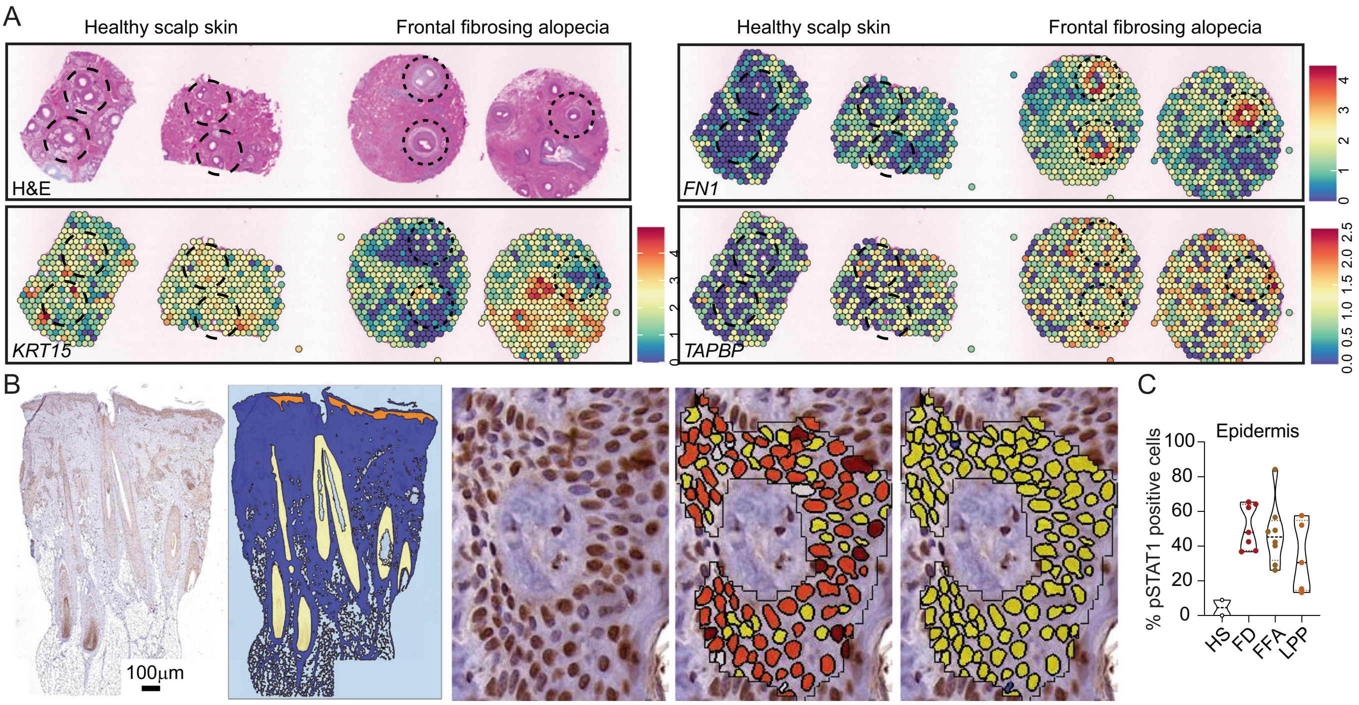

Figure EV5. Key features of scarring alopecia in spatial transcriptomics and quantification of phosphorylated STAT1 in human clinical samples.

(**A**) Spatial transcriptomic feature plots of the indicated genes superimposed on the corresponding H&E images. The H&E image is also available at Fig. 6C. Dataset from Cohen et al (Data ref: Cohen et al, 2024). (**B**) Nuclear staining intensity was quantified using definiens software as indicated. Hair follicles (yellow area) and epidermis (orange area) were separately analyzed. Medium intensity is shown in red. (**C**) Quantification of pSTAT1 staining intensity in the epidermis, $n \geq 2$.

© The Author(s)
